# Leveraging chromatin packing domains to target chemoevasion in vivo

**DOI:** 10.1073/pnas.2425319122

**Published:** 2025-07-22

**Authors:** Jane Frederick, Ranya K. A. Virk, I Chae Ye, Luay M. Almassalha, Greta M. Wodarcyk, David VanDerway, Ruyi Gong, Cody L. Dunton, Tiffany Kuo, Karla I. Medina, Margarita Loxas, Jared T. Ahrendsen, Demirkan B. Gursel, Paola Carrillo Gonzalez, Rikkert J. Nap, Saira John, Vasundhara Agrawal, Nicholas M. Anthony, John Carinato, Wing Shun Li, Rivaan Kakkaramadam, Surbhi Jain, Shohreh Shahabi, Guillermo A. Ameer, Igal G. Szleifer, Vadim Backman

**Affiliations:** ^a^Department of Biomedical Engineering, Northwestern University, Evanston, IL 60208; ^b^Center for Physical Genomics and Engineering, Department of Biomedical Engineering, Northwestern University, Evanston, IL 60208; ^c^Chemistry of Life Processes Institute, Department of Biomedical Engineering, Northwestern University, Evanston, IL 60208; ^d^Department of Pharmaceutical Chemistry, University of California, San Francisco, CA 94158; ^e^Department of Gastroenterology and Hepatology, Feinberg School of Medicine, Northwestern University, Chicago, IL 60611; ^f^Department of Pathology, Feinberg School of Medicine, Northwestern University, Chicago, IL 60611; ^g^Department of Obstetrics and Gynecology, Feinberg School of Medicine, Prentice Women’s Hospital, Northwestern University, Chicago, IL 60611; ^h^Department of Chemistry, Northwestern University, Evanston, IL 60208

**Keywords:** chromatin, cancer, chemotherapy, plasticity, Biophysics

## Abstract

Cancer cells exhibit a remarkable resilience to cytotoxic stress, often adapting through transcriptional changes linked to alterations in chromatin structure. In several types of cancer, these adaptations involve epigenetic modifications and restructuring of topologically associating domains. However, the underlying principles by which chromatin architecture facilitates such adaptability across different cancers remain poorly understood. To investigate the role of chromatin in this process, we developed a physics-based model that connects chromatin organization to cell fate decisions, such as survival following chemotherapy. Our model builds on the observation that chromatin forms packing domains, which influence transcriptional activity through macromolecular crowding. The model accurately predicts chemoevasion in vitro, suggesting that changes in packing domains affect the likelihood of survival. Consistent results across diverse cancer types indicate that the model captures fundamental principles of chromatin-mediated adaptation, independent of the specific cancer or chemotherapy mechanisms involved. Based on these insights, we hypothesized that compounds capable of modulating packing domains, termed Transcriptional Plasticity Regulators (TPRs), could prevent cellular adaptation to chemotherapy. We conducted a proof-of-concept compound screen using live-cell chromatin imaging to identify several TPRs that synergistically enhanced chemotherapy-induced cell death. The most effective TPR significantly improved therapeutic outcomes in a patient-derived xenograft model of ovarian cancer. These findings underscore the central role of chromatin in cellular adaptation to cytotoxic stress and present a framework for enhancing cancer therapies, with broad potential across multiple cancer types.

## Significance

Gaining insight into cancer cell adaptability is critical for optimizing therapeutic strategies. This study introduces a biophysical framework that links chromatin structure to therapy resistance, addressing a critical gap in our understanding of cancer cell behavior. By integrating live-cell chromatin imaging with physics-based modeling, we reveal how chromatin organization influences cell-fate decisions through its effects on transcriptional responsiveness. Our approach, grounded in the first principles of physics, accurately predicts cell survival under cytotoxic stress across diverse cancer types. Importantly, we demonstrate that coadministering chemotherapy with agents that target chromatin packing domains significantly enhances efficacy in vitro and in vivo. This new strategy offers a promising approach to combat treatment resistance, potentially transforming outcomes for cancer patients.

While genetic resistance is a well-established barrier to chemotherapy efficacy (1, 2), mutations take months to evolve (3). In contrast, cancer cells lacking preexisting resistance mutations must make survival decisions within hours of treatment (4), implicating nongenetic mechanisms as key determinants of the immediate therapeutic response.

As chromatin structure governs transcriptional responses to stress, chromatin-mediated transcriptional plasticity has emerged as a key driver of rapid cellular adaptation (5, 6). Alterations in chromatin are known to promote oncogene activation and chemoresistance. For example, disrupting topologically associating domain (TAD) boundaries can enable enhancer hijacking, while large-scale compartmental mixing drives transcriptional reprogramming (7–9). Although epigenetic modulators enhance chemotherapeutic efficacy in hematologic cancers (10, 11), their limited success in more heterogeneous solid tumors (12–14) underscores the need for alternative frameworks to guide therapeutic strategies.

We propose that a polymer-physics-based understanding of chromatin is necessary to better understand its effects on the transcriptional response to chemotherapy. The inherently crowded nature of the nucleus influences transcription through macromolecular crowding (15–17). Therefore, the statistical distribution of the chromatin density should influence gene expression patterns. Previously, we have shown that chromatin packing domains, nanoscale structures smaller than TADs characterized by mass fractal properties, are key regulatory units in transcriptional plasticity (18–20). These domains are densely packed regions of chromatin ranging in radius from 60 to 90 nm and containing 80 to 200 kbp of genomic material (19, 21). Their high surface area-to-volume ratio enables efficient DNA packaging while maintaining accessibility for rapid transcriptional changes, a crucial component of adaptability.

To investigate the role of packing domains in therapy resistance, we developed a Chromatin-Dependent Adaptability (CDA) model that links packing domain properties to transcriptional responses and cell fate decisions. The model predicts that a higher average packing domain scaling ($D_{n}$) increases survival under treatment. We validated this prediction through live cell and superresolution chromatin imaging (22, 23). Our findings motivated a proof-of-concept screen for Transcriptional Plasticity Regulators (TPRs) that modulate packing domains. Notably, the top TPRs enhanced chemotherapy response both in vitro, across multiple cancer cell lines and drug models, and in vivo in a patient-derived xenograft (PDX) model. Altogether, we establish a new framework for targeting chromatin-dependent transcriptional plasticity to improve therapeutic outcomes.

## Results

### A Biophysical Model Linking Chromatin Packing Domains to Chemoevasion.

**Fig. 1.**
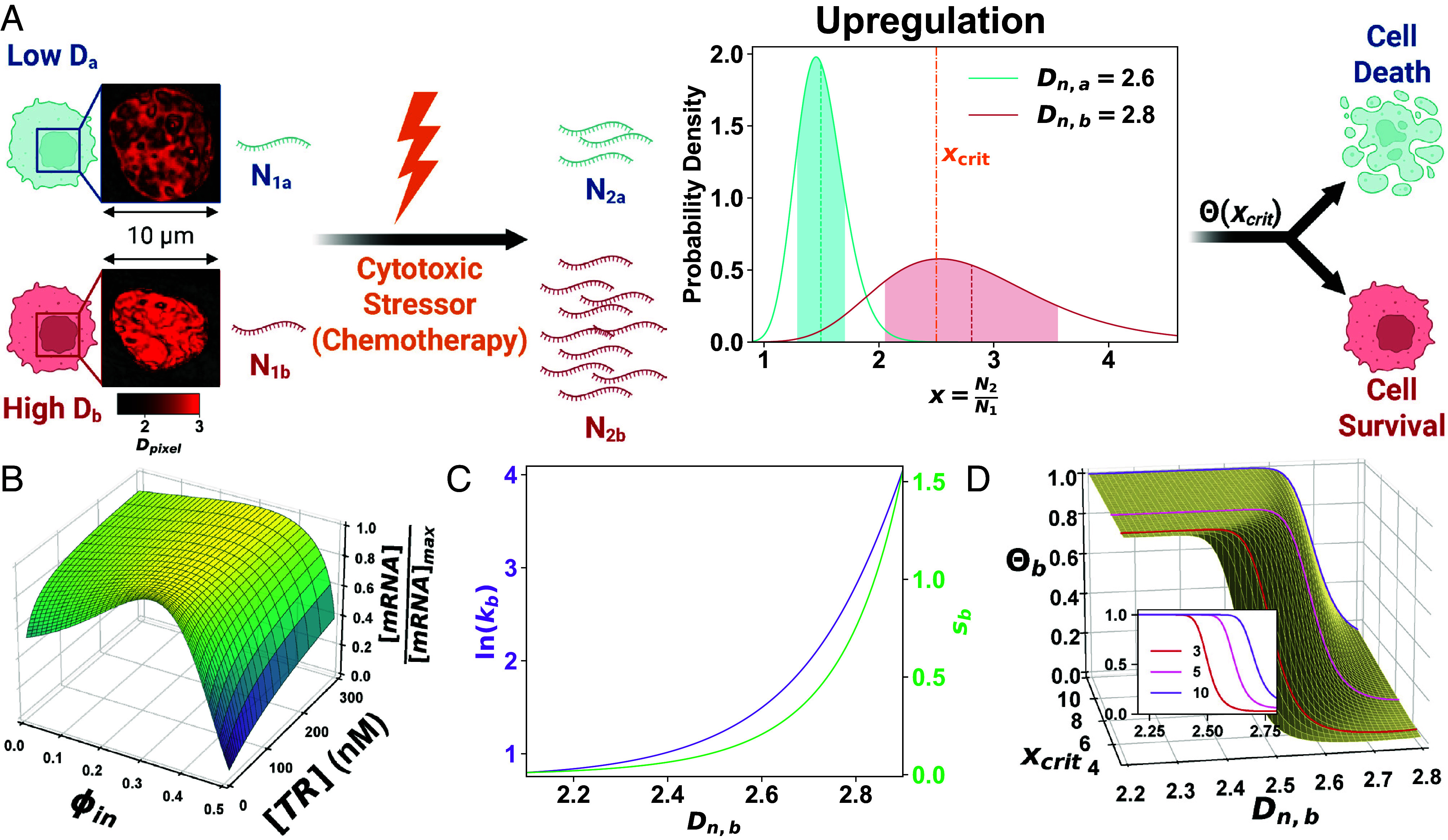
The Chromatin-Dependent Adaptability (CDA) model links chromatin organization to cell survival under cytotoxic stress via transcriptional plasticity. (*A*) Schematic showing differential responses of cells with low ($D_{n}=2.6$, blue) vs. high ($D_{n}=2.8$, red) average nuclear chromatin packing domain scaling to cytotoxic stress. Representative PWS images illustrate corresponding nuclear structure (scale = 10 μM; color = $D_{\text{pixel}}$). PDFs of gene upregulation ($x=N_{2}/N_{1}$) indicate that higher $D_{n}$ increases both mean and variability, surpassing $x_{\text{crit}}$ and enhancing survival probability. (*B*) 3D surface plot of normalized mRNA concentration as a function of transcriptional reactant (TR) concentration and local crowding ($\phi_{\text{in}}$). (*C*) Dependence of transcriptional malleability (k, purple) and heterogeneity (s, green) on $D_{n}$ for cell b; $\ln(E/\bar{E})=-5.5$, $\beta_{a}=10$. (*D*) Cell death probability ($\Theta_{b}$) vs. $D_{n}$ across varying upregulation thresholds ($x_{\text{crit}}$); the *Inset* shows selected curves.

To investigate how pretreatment chromatin organization influences cell survival, we extended our Chromatin Packing Macromolecular Crowding (CPMC) model—which integrates molecular and physical transcriptional regulators—to predict how packing domains modulate gene expression (Fig. 1*A*) (18, 24). We then adapted this framework to estimate the probability of cell death based on chromatin-mediated transcriptional responses to chemotherapy.

#### Packing domains regulate gene transcription through macromolecular crowding.

Macromolecular crowding, primarily driven by chromatin density in the nucleus, alters the kinetics and efficiency of transcription reactions (16). This effect is nonmonotonic: Moderate crowding enhances binding of transcriptional reactants (TRs), while excessive crowding restricts diffusion and inhibits transcription (15, 17).

The distribution of chromatin density within packing domains is expected to modulate the transcriptional activity of embedded genes. Packing domains are densely packed mass-fractal structures that exhibit a power-law relationship between their genomic size ($N_{\text{PD}}$, in base pairs) and spatial radius ($r_{\text{PD}}$), described by $N_{\text{PD}}∼r_{\text{PD}}^{D_{\text{PD}}}$, where $D_{\text{PD}}$ is the scaling exponent that describes DNA packing (19, 21). The crowding fraction within the transcriptional interaction volume of a domain, denoted $\phi_{\text{in}}$, reflects local density within that domain.

Chromatin packing domains contain dense, transcriptionally repressive heterochromatic cores surrounded by more open euchromatic peripheries (20, 23, 25). This density gradient creates spatial variations in crowding that influence protein diffusion and activity. Genes located at the periphery are more likely to occupy favorable transcriptional environments (19), while those in the core are typically silenced. We quantify the proportion of peripheral, transcriptionally accessible chromatin using the exposure ratio (ER), which follows the fractal relationship $\text{ER}\approx {\left(N_{\text{PD}}/A_{v}\right)}^{-1/D_{\text{PD}}}$ (21). $A_{v}$ describes how densely chromatin is arranged within a domain, with a value of 1 indicating ideal fractal packing. Smaller domains with higher $D_{\text{PD}}$ tend to exhibit greater ER. Collectively, these structural features determine the probability that a gene resides in a transcriptionally permissive environment.

Our transcription model (15, 17) reveals that these packing domains support two distinct modes of transcriptional adaptation to stress. Front-loading (or transcriptional priming) refers to sustained high gene expression driven by consistently favorable molecular conditions, such as increased levels of TRs (i.e., RNA polymerase II and transcription factors). These genes with high initial TR concentrations exhibit minimal sensitivity to local crowding $\phi_{\text{in}}$ (Fig. 1*B*). In contrast, transcriptional plasticity describes the rapid activation of genes that are initially underexpressed with low TR availability, which are more sensitive to crowding (Fig. 1*B*). Thus, high $D_{\text{PD}}$ domains with more permissive crowding are predicted to accelerate the upregulation of these genes.

#### The CPMC model predicts transcriptional responses from packing domain structure.

The CPMC model provides a high-level framework for predicting how chromatin packing domains regulate transcriptional dynamics on a genome-wide scale (18, 24). Because direct measurement of packing domain structures for all genes is not experimentally feasible, we use a probabilistic approach. We define the nuclear average packing scaling parameter, $D_{n}$, as $D_{n}\approx \left\langle D_{\text{PD}}\right\rangle \cdot \text{VF}$, where VF is the volume fraction of the nucleus occupied by packing domains.

To isolate the structural influence of packing domains from molecular determinants, we group genes by shared molecular characteristics $\vec{m}$ (e.g., TR concentration). For each group, the ensemble expression rate E is computed as $E=\bar{\epsilon }\cdot \text{ER}$ (18, 24). $\bar{\epsilon }$ is the average expression rate of these genes with similar molecular features $\vec{m}$:

[1] $$\bar{\epsilon }=\int \epsilon \left(\vec{m},\phi_{\text{in}}\right)f\left(\phi_{\text{in}}\right),d\phi_{\text{in}}$$

Here, $\epsilon (\vec{m},\phi_{\text{in}})$ denotes the expression rate under given molecular and crowding conditions. $f(\phi_{\text{in}})$ is the crowding distribution within packing domains and acts as the weighting function in the integral. $\bar{\epsilon }$ can also be approximated via Taylor series expansion (*SI Appendix*, Fig. S1) (18, 24).

To evaluate the transcriptional consequences of packing domains, we calculate the sensitivity (Se) of gene expression E to the initial properties of domains. Specifically, the sensitivity of expression to $D_{n}$ is defined as $Se\left(D_{n}\right)=\frac{\partial \ln(E)}{\partial \ln(D_{n})}|E=E_{i},D_{n}=D_{n,i}$, where i=1,2 corresponds to the pre- and posttreatment states. An analytical approximation of the sensitivity directly demonstrates the influence of $D_{n}$ on Se (*SI Appendix*). Among all structural and molecular parameters evaluated, expression is most sensitive to $D_{n}$ (*SI Appendix*, Fig. S2). Therefore, we focus on $Se(D_{n})$ throughout this work.

To illustrate the predictive capacity of the model, we consider two cells, a and b, such that $D_{n,b}\;>\;D_{n,a}$. Without loss of generality, this comparison can refer to either individual cells or distinct populations. Let $Se_{i}$ denote the sensitivity before (i=1) and after (i=2) chemotherapy treatment, and let $\beta =E_{2}/E_{1}$ represent the change in expression rate between the two states. Then, if $\beta_{a}$ is known, $\beta_{b}$ is given by:

[2] $$\beta_{b}=\beta_{a}\exp\left[\int_{D_{n,a}}^{D_{n,b}}\frac{Se_{2}\left(D_{n}^{\prime }\right)-Se_{1}\left(D_{n}^{\prime }\right)}{D_{n}^{\prime }},dD_{n}^{\prime }\right]$$

Thus, the CPMC model provides a quantitative framework linking chromatin packing domains to gene expression changes under stress.

#### The CDA model links transcriptional response to cell fate decisions.

In cancer cells lacking front-loaded gene expression, survival under chemotherapy requires transcriptional activation of key genes within a critical time frame. For example, the tumor suppressor p53 must reach a threshold level to trigger apoptosis (26), and its rapid accumulation is observed in cells undergoing apoptosis after chemotherapy (27). Motivated by this, we developed a framework in which the probability of cell death after chemotherapy depends on the probability distribution function (PDF) of chromatin-mediated transcriptional upregulation. Although we focus here on activation, downregulation can be modeled using a similar formalism.

We define the transcript ratio $x=N_{2}/N_{1}$ as the fold change in mRNA transcripts before ($N_{1}$) and after ($N_{2}$) treatment. Assuming gene expression follows a log-normal distribution (28), the distribution of x is approximated by:

[3] $$\text{PDF}\left(x\right)\approx \frac{1}{s\sqrt{2\pi }x}\exp\left(-\frac{\ln{\left(x/k\right)}^{2}}{2s^{2}}\right)$$

Here, k represents the typical fold-change in expression (transcriptional malleability), and s reflects variability in that change (transcriptional heterogeneity). When s≪1, the mean expression change is approximately ln(*k*), and s can be estimated by the coefficient of variation (COV) of the expression changes. In this framework, malleability (k) captures the expected capacity of a cell for transcriptional adaptation, while heterogeneity (s) quantifies the variability of this response across the population.

Our CPMC model yields the change in expression rate $\beta =E_{2}/E_{1}$ following chemotherapy. To estimate the evolution of transcript levels over time, we use a first-order exponential adaptation model:

[4] $$k\left(t\right)=\frac{N_{2}\left(t\right)}{N_{1}}=1+\left(\beta -1\right)\left(1-e^{-t/\tau }\right)$$

Here, $\tau =\frac{1}{\ln2}\tau_{1/2}$ is the mRNA elimination time constant, with $\tau_{1/2}=10$ h as the mRNA half-life (29).

Transcriptional heterogeneity is given by:

[5] $$s\left(t\right)=\text{COV}\left[\frac{N_{2}}{N_{1}}\right]=\beta \frac{1-e^{-t/\tau }}{k(t)}\sqrt{{\text{COV}}_{1}^{2}+{\text{COV}}_{2}^{2}}$$

The coefficients of variation COV depend on $\phi_{\text{in}}$ and $D_{n}$ (*SI Appendix*). Thus, heterogeneity is dynamically regulated by initial chromatin organization and malleability k.

We substitute the expression change from Eq. **2** into Eqs. **4** and **5** to analytically predict the probability of transcriptional adaptation to chemotherapy as a function of $D_{n}$. The CDA model links this transcriptional response to cell fate by assuming that survival requires reaching a critical expression threshold, $x_{\text{crit}}$, within a defined time window, $t_{\text{crit}}$. Higher $D_{n}$ values are associated with greater transcriptional malleability (k) and heterogeneity (s) (Fig. 1*C*), increasing the chance of reaching this threshold and reducing the likelihood of death.

To formalize this intuition, the probability of cell death, Θ, is modeled using the cumulative distribution function (CDF) of the transcript threshold $x_{\text{crit}}$. This distribution has a sigmoidal shape that can be approximated by a Hill equation (*SI Appendix*). For two cells, a and b, with distinct chromatin packing values $D_{n,a}$ and $D_{n,b}$, the death probability for cell b is

[6] $$\Theta \left(x_{\text{crit}}\right)\approx \frac{1}{1+{\left(k_{a}\gamma_{k}/x_{\text{crit}}\right)}^{\frac{h_{a}}{\gamma_{h}}}}$$

Here, $h=\frac{3}{\sqrt{\pi }s}$ is the inverse COV or heterogeneity. The term $\gamma_{k}=k_{b}/k_{a}$ represents the relative malleability between cells and $\gamma_{h}=h_{a}/h_{b}=s_{b}/s_{a}$ quantifies their relative heterogeneity.

The relationship between $D_{n,b}$ and death probability Θ is primarily governed by the upregulation threshold $x_{\text{crit}}$ and the expression level in cell a ($\beta_{a}$). The CDA model predicts that increasing $x_{\text{crit}}$ increases the likelihood of death in high-$D_{n}$ cells (Fig. 1*D*), while greater β in a low-$D_{n}$ cell a reduces the advantage conferred by high $D_{n}$ in cell b (*SI Appendix*, Fig. S3*A*). Other variables, such as baseline expression ($\text{ln}(E/\bar{E})$, determined by molecular factors $\vec{m}$) and the time window $t_{\text{crit}}$, have relatively minor effects (*SI Appendix*, Fig. S3 *B* and *C*). Constants and other parameters used for the model can be found in *SI Appendix*, Tables S1 and S2.

In summary, the CDA model establishes a physics-based framework linking chromatin structure to cellular adaptability through transcriptional regulation. When $D_{n,b}\;>\;D_{n,a}$, the model predicts greater transcriptional malleability and heterogeneity, shifting the log-normal distribution of gene activation upward and reducing the likelihood of cell death (Fig. 1*C*–*E*). We next experimentally test these predictions across diverse cancer cell lines by measuring temporal changes in $D_{n}$ and their impact on survival after chemotherapy.

### Pretreatment Packing Domain Organization Influences Cancer Cell Survival Across Cell Types and Cytotoxic Mechanisms.

To test the CDA model prediction that prestress chromatin structure influences cell death, we used a live-cell imaging modality that tracks chromatin dynamics over time, linking initial chromatin states to survival outcomes. We employed Partial Wave Spectroscopic (PWS) microscopy, a label-free optical technique that captures wavelength-dependent variations in backscattered interference spectra (22). These spectral variations reflect nanoscale differences in mass density and provide insight into chromatin structure (30). PWS is sensitive to length scales from ∼20 to 300nm (31), enabling detection of chromatin features ranging from nucleosomes (∼10nm) to higher-order packing domains (∼160nm diameter). The measured spectral signal corresponds to the spatial autocorrelation of chromatin mass density, and its shape defines the nuclear chromatin organization parameter $D_{n}$ (32). This allows real-time monitoring of population-level $D_{n}$ through time-course imaging, producing pixel-wise maps of packing domains, where each pixel’s $D_{\text{pixel}}$ value reflects the average of domains within that pixel. To quantify drug effects, we averaged $D_{\text{pixel}}$ values across individual nuclei to determine $D_{n}$ and analyzed population-wide distributions (*SI Appendix*). Since PWS imaging is performed at the cell–glass interface, only viable, adherent cells were captured as apoptotic cells rapidly detach (33). Therefore, observed changes in $D_{n}$ distributions reflect surviving cells after cytotoxic treatment.

We evaluated changes in chromatin organization within cancer cell populations after chemotherapy treatment by imaging at biologically relevant time points. Previous studies have shown that a two-hour pulse of platinum-based drugs can induce chemoresistance within two days (34). In HCT116 colon cancer cells, oxaliplatin can initiate apoptosis within six hours, leading to a significant increase in membrane-permeable dead cells by 24 h (35). Based on these kinetics, we imaged cell populations at six time points: before treatment (0 h) and at 2, 6, 12, 24, and 48 h posttreatment.

**Fig. 2.**
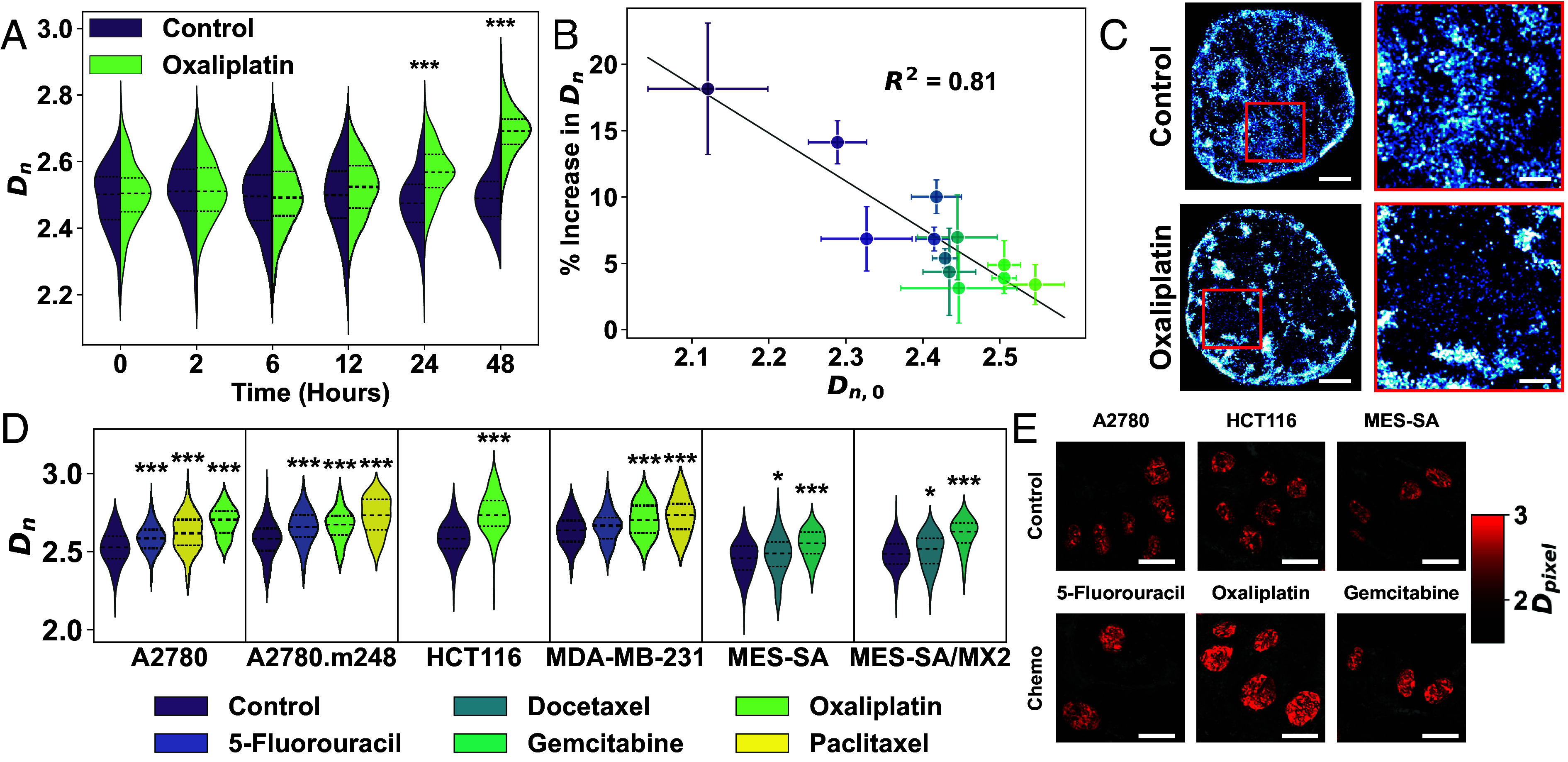
Cells that survive chemotherapy exhibit altered chromatin structure. (*A*) Split violin plots of $D_{n}$ in HCT116 cells over 48 h of 15 µM oxaliplatin. n=70 to 150 cells/condition. (*B*) Change in average $D_{n}$± SE of the mean (SEM) per HCT116 cell cluster after 48 h treatment, relative to baseline ($D_{n,0}$± SEM). Initial clusters: 2 to 5 cells; final: 1 to 12. (*C*) Representative SMLM images (EdU-labeled HCT116 cells): control (*Top*) and oxaliplatin-treated (*Bottom*). Pseudocolor shows localization density (white = high). Red boxes mark zoomed regions (*Right*). [Scale bars: 2 µm (full), 0.5 µm (zoom).] (*D*) Violin plots of $D_{n}$ in surviving cells across treatments and cancer lines: A2780, A2780.m248, MDA-MB-231 (vehicle, 5-FU, paclitaxel, oxaliplatin); HCT116 (vehicle, oxaliplatin); MES-SA, MES-SA/MX2 (vehicle, docetaxel, gemcitabine). (*E*) Representative PWS images after 48 h treatment: A2780 (5-FU, 0.5 µM), HCT116 (oxaliplatin, 15 µM), MES-SA (gemcitabine, 50nM). Brighter red = higher $D_{\text{pixel}}$. Scale: 10 µm.Significance: ^∗^*P* < 0.05, ^∗∗∗^*P* < 0.001 (unpaired two-tailed *t* test vs. control).

The CDA model predicts that cells with elevated $D_{n}$ are more likely to survive chemotherapy. To test this, we monitored population-level changes in $D_{n}$ over time, reasoning that high-$D_{n}$ cells would preferentially persist. Chemotherapy induced a time-dependent increase in $D_{n}$, which became statistically significant at 24 h posttreatment (P<0.001; Fig. 2*A*), coinciding with markers of apoptosis (*SI Appendix*, Fig. S4). This shift emerged by 12 h and continued through 48 h, with mean increases of ∼4% at 24 h and ∼8% at 48 h (P<0.001). In contrast, untreated controls showed no significant change in $D_{n}$ over the same period.

Although population-level measurements showed average increases in $D_{n}$, they did not reveal how cells with specific initial chromatin states change over time. Because chromatin organization is more similar among related cells (19), we tracked small clusters of 2 to 5 HCT116 cells to assess how baseline $D_{n}$ influences chemotherapy response. Cluster-level analysis showed a strong inverse correlation ($R^{2}\;=\;0.81$) between initial $D_{n}$ ($D_{n,0}$) and posttreatment increase (Fig. 2*B*). Clusters with lower $D_{n,0}$ increased more, while those with higher $D_{n,0}$ changed little. Notably, 9 clusters with $D_{n}\;>\;2.4$ survived, compared to just 3 with $D_{n}\;<\;2.4$. These results underscore the influence of initial chromatin structure on treatment outcomes.

PWS imaging showed elevated $D_{n}$ in oxaliplatin-treated cells, but this bulk measurement cannot resolve whether changes in packing domain size underlie the shift. Because PWS lacks the resolution to visualize individual domains, we labeled newly synthesized DNA with EdU and used Single Molecule Localization Microscopy (SMLM) to image packing domains in control and treated nuclei (20). Domains were identified using Density-Based Spatial Clustering of Applications with Noise (DBSCAN) on reconstructed images. We applied thresholds from prior ChromSTEM analysis (21) to exclude clusters smaller than 40 nm or larger than 250 nm that are not likely to be domains. SMLM images of oxaliplatin-treated cells revealed a distinct chromatin organization with more small domains (Fig. 2*C* and *SI Appendix*, Fig. S5). These findings suggest that surviving cells have altered chromatin organization, potentially contributing to their response to cytotoxic stress.

We next tested whether chemotherapy increases $D_{n}$ in other cancer types and with non-platinum-based agents. Six cancer cell lines were treated with agents from three major chemotherapy classes: DNA intercalators (oxaliplatin), microtubule inhibitors (paclitaxel, docetaxel), and nucleotide analogs (5-fluorouracil, gemcitabine), using clinically relevant doses (*SI Appendix*, Table S3). Ovarian (A2780, A2780.m248) and breast (MDA-MB-231) cancer cells were treated with paclitaxel, oxaliplatin, or 5-fluorouracil (5-FU) (36–38); colon cancer cells (HCT116) received oxaliplatin; and uterine leiomyosarcoma cells (MES-SA, MES-SA/MX2) were treated with docetaxel or gemcitabine (39–41). In most drug-cell line combinations, chemotherapy significantly increased $D_{n}$ compared to controls (P<0.001; Fig. 2 *D* and *E*). Notably, the magnitude of $D_{n}$ elevation corresponded with known treatment efficacy. Gemcitabine, effective in soft tissue sarcoma, induced large $D_{n}$ increases (P<0.001), whereas the less effective docetaxel produced smaller changes (42). Likewise, 5-FU, which has limited efficacy as monotherapy (43) but is used in combination regimens (36, 38), induced the smallest $D_{n}$ shifts in ovarian and breast cancer cells. Together, these findings suggest that $D_{n}$ increases are a generalizable response to chemotherapy and correlate with drug efficacy across diverse cancer types.

Given the observed link between increased $D_{n}$ and chemotherapy efficacy, we hypothesized that $D_{n}$ may stay elevated in cells with stable drug resistance. To test this, we examined two resistance models: one involving a tumor suppressor point mutation and another with inherent drug resistance. In the first model, A2780 cells with TP53 mutations—common in high-grade serous ovarian cancer—showed higher $D_{n}$ in two subclones vs. wild-type cells (*SI Appendix*, Fig. S6 *A* and *C*). We also related these findings to TCGA survival data from patients with the same TP53 mutations (44), revealing a link between elevated $D_{n}$ and lower overall survival (*SI Appendix*, Fig. S6*B*). In the second model, a drug-resistant MES-SA subclone showed increased $D_{n}$ compared to its drug-sensitive counterpart (*SI Appendix*, Fig. S6 *D* and *E*). Together, these results show consistent $D_{n}$ elevation in resistant cells across models and cancer types, supporting the broader relevance of the CDA framework.

### Chromatin Dynamics Predict Chemotherapeutic Response in Cancer Cells.

**Fig. 3.**
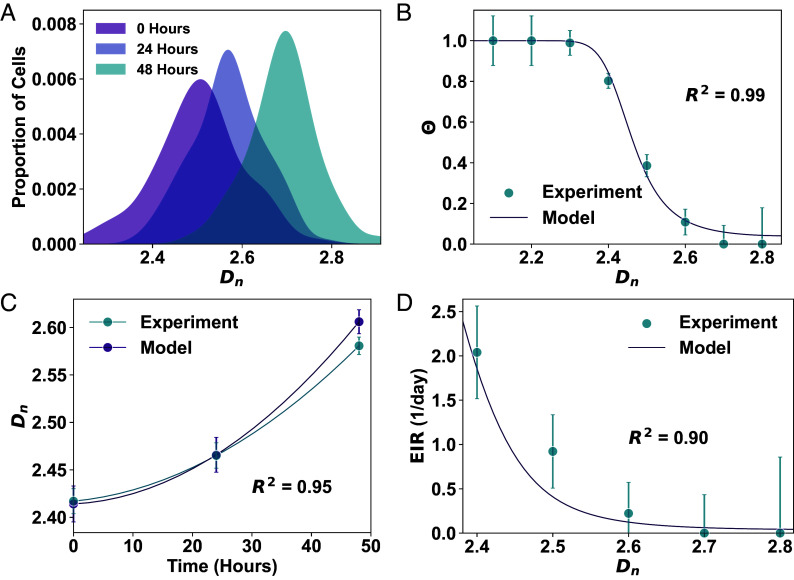
Experimental validation of model predictions linking chromatin structure to chemotherapy response. (*A*) PDFs of $D_{n}$ in HCT116 cell clusters at 0 (purple), 24 (blue), and 48 (teal) hours after 15 µM oxaliplatin treatment, showing a progressive shift toward higher $D_{n}$. (*B*) Cell death probability (Θ) vs. $D_{n}$, with experimental values (blue points ± SEM) from tracked HCT116 clusters during 48 h treatment. The solid purple line shows CDA model prediction (Eq. **6**; $x_{\text{crit}}\approx 3$, $\beta_{a}\approx 7$, $\text{ln}(E/\bar{E})\approx 2.5$; MSE = 0.001). (*C*) Comparison of model-predicted (purple) and experimentally observed (blue) increases in mean $D_{n}$± SEM in HCT116 clusters over 48 h oxaliplatin treatment. (*D*) EIR over time, quantifying cumulative cell death from oxaliplatin, plotted against $D_{n}$. Experimental (blue ± SEM) and model-predicted (purple line) values shown.

To evaluate whether the CDA model quantitatively predicts the relationship between chromatin structure and cell death, we used HCT116 cluster data from Fig. 2*B* to calculate experimental $\Theta (D_{n})$ values. Unlike population-level measurements, cluster tracking provided matched $D_{n}$ values at 0, 24, and 48 h for the same dividing clusters, allowing us to assess how initial chromatin states influence outcomes. First, we verified that the $D_{n}$ distribution shifted toward higher values in surviving clusters (Fig. 3*A*). We then focused on actively cycling clusters, where $D_{n}$ is highly correlated due to inheritance of chromatin architecture (19), and excluded quiescent cells. After correcting for division-related drift, we determined experimental values of Θ by comparing the 24- and 48-h distributions to predicted distributions from the 0-h baseline (*SI Appendix*). We then fit Eq. **6** to the experiment $\Theta (D_{n})$ using three free parameters ($x_{\text{crit}}$, $\beta_{a}$, $\ln(E/\bar{E})$). Note that this approach assumes uniform expression-related parameters throughout the cell population, which represents a limitation of our method. Despite its simplicity, the model accurately captured the relationship between $D_{n}$ and cell death with low root mean square error (RMSE = 0.035; Fig. 3*B*). Parameter estimation revealed that genes relevant for cell survival require considerable upregulation ($\beta_{a}\approx 7$) to meet a moderately high threshold ($x_{\text{crit}}\approx 3$). The fit to experimental data was primarily influenced by the ratio $\beta_{a}/x_{\text{crit}}$, rather than by molecular factors such as TR concentration (*SI Appendix*, Fig. S7). Overall, this suggests that HCT116 cells must substantially upregulate key survival genes to reach the activation threshold required for viability. The model’s limited sensitivity to baseline expression further implies that survival depends more on a gene’s capacity for rapid upregulation than on its initial expression level.

Having shown that the CDA model accurately predicts cell death probabilities, we next asked whether it could also capture the observed increases in $D_{n}$ over time during chemotherapy. We hypothesized that population-level $D_{n}$ reflects the cumulative survival of individual cells across successive divisions. Letting $n_{\tau }$ represent the number of cell divisions since treatment and $\tau_{2}\approx 18$ h (HCT116 doubling time), we modeled survival at each interval as $1-\Theta (D_{n})$. The average $D_{n}$ at time $t_{n_{\tau }}$ was computed by weighting each $D_{n}$ by its survival probability over $n_{\tau }$ divisions:

[7] $$\left\langle D_{n}\left(t_{n_{\tau }}\right)\right\rangle =\frac{\int D_{n}\cdot \text{PDF}\left(D_{n}\right){\left[1-\Theta \left(D_{n}\right)\right]}^{n_{\tau }},dD_{n}}{\int \text{PDF}\left(D_{n}\right){\left[1-\Theta \left(D_{n}\right)\right]}^{n_{\tau }},dD_{n}}$$

The model predicted a time-dependent increase in $D_{n}$, consistent with experimental observations (RMSE = 0.015; Fig. 3*C*).

To quantify how initial chromatin structure affects overall treatment efficacy, we introduced the effective inhibition rate (EIR)—a metric that summarizes the cumulative impact of chemotherapy on cell survival over time. We defined EIR by first modeling the survival probability at time $t_{n_{\tau }}$ as

[8] $$P_{n_{\tau }}=\int \text{PDF}\left(D_{n}\right)[1-\Theta \left(D_{n}\right){]}^{n_{\tau }}dD_{n}$$

This expression defines EIR via an exponential decay function:

[9] $$\text{EIR}\left(t_{n_{\tau }}\right)=\frac{1}{t_{n_{\tau }}}\ln\frac{1}{{\left(1-\Theta \left(D_{n}\right)\right)}^{n_{\tau }}}$$

EIR thus serves as a decay constant quantifying the rate at which chemotherapy eliminates cells over time. Model predictions and experimental data both revealed that EIR decreases exponentially with increasing $D_{n}$ (RMSE = 0.25 per day; Fig. 3*D*), indicating that even modest increases in chromatin packing substantially reduce treatment efficacy. These findings highlight the therapeutic potential of targeting chromatin organization to enhance chemotherapeutic response.

### Modulating Chromatin Structure Sensitizes Cancer Cells to Chemotherapy.

Building on our finding that higher $D_{n}$ is associated with greater chemotherapy resistance, we investigated whether chromatin-modifying agents could enhance treatment efficacy by reducing $D_{n}$. We define TPRs as compounds that lower $D_{n}$, thereby limiting the transcriptional adaptability of cancer cells. An effective TPR should selectively reduce $D_{n}$ in cancer cells with minimal effects on noncancerous cells, improving therapeutic outcomes while minimizing off-target toxicity.

**Fig. 4.**
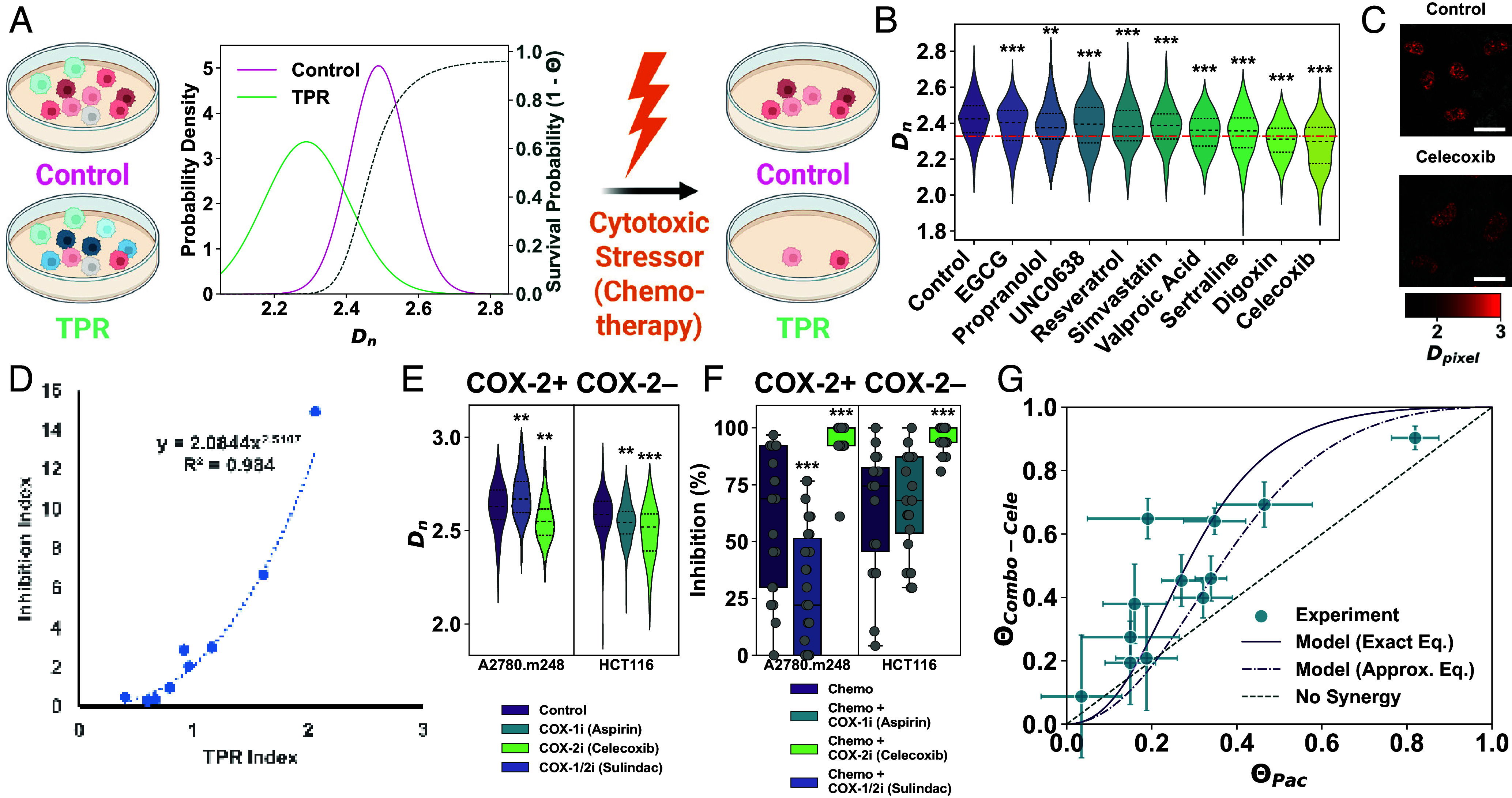
TPRs modulate chromatin structure to enhance chemotherapeutic efficacy in vitro. (*A*) Schematic of differential survival in cancer populations with distinct PDF($D_{n}$) under chemotherapy. TPR-treated cells (green) show reduced $D_{n}$ and survival probability compared to control (magenta); the dashed gray line denotes 1−Θ. (*B*) Proof-of-concept TPR screen in A2780 cells showing $D_{n}$ distributions across conditions. The dashed red line indicates critical threshold $D_{n,\text{crit}}=2.33$ (Θ>0.99). (*C*) Representative PWS images of control and celecoxib-treated A2780 cells. Pseudocolor reflects $D_{\text{pixel}}$ (higher = brighter red). (Scale bars: 10 µm.) (*D*) TPR index (chromatin change) vs. inhibition index (chemotherapy efficacy), showing exponential relationship (fit: $y=2.0844e^{2.0107x}$; $R^{2}\;=\;0.98$). (*E*) Violin plots of $D_{n}$ in COX-2+ (A2780.m248) and COX-2- (HCT116) cells treated with aspirin (COX-1i), celecoxib (COX-2i), or sulindac (COX-1/2i); 100 to 200 cells per condition. (*F*) Box plots of percent inhibition in A2780.m248 (paclitaxel) and HCT116 (oxaliplatin) cells with and without COX inhibitors; each box: 20 to 30 populations. (*G*) Cell death comparison in A2780 cells: paclitaxel alone ($\Theta_{\text{Pac}}$) vs. paclitaxel + celecoxib, normalized by celecoxib alone ($\Theta_{\text{Combo-Cele}}$). Experimental data (blue ± SEM); solid/dashed purple lines show exact (Eq. **12**) and approximate (Eq. **13**) CDA predictions. Dashed gray line: no synergy expected. (*B*, *E*, and *F*) Significance: ^∗∗^*P* < 0.01, ^∗∗∗^*P* < 0.001 (unpaired two-tailed *t* test with unequal variance vs. control).

Our model predicts that isogenic cell populations with higher $D_{n}$ exhibit greater survival under identical cytotoxic stress, making overall population survival dependent on the fraction of high-$D_{n}$ cells (Fig. 4*A*). To identify effective TPR compounds, we conducted a targeted, proof-of-concept screen for agents that reduce $D_{n}$ within one hour. This rapid timeframe suggests a direct physical impact on nuclear structure, rather than slower, gene expression-dependent mechanisms. Short exposures also limit the window for adaptive responses during early chemotherapy treatment. To prioritize candidates, we used the CDA model to determine a critical $D_{n}$ threshold associated with near-complete cell death (Θ≈0.99), identifying $D_{n,\text{crit}}=2.33$ as the benchmark for effective TPR activity in A2780 cells.

Our selection of TPR candidates was guided by current insights into the mechanisms of chromatin packing domain formation and stabilization, which are governed by three key principles (20). First, domains form in low-density nuclear regions via loop-forming processes like cohesin-mediated extrusion and transcription. Second, higher chromatin density enriches small proteins like histone methyltransferases in domain cores, promoting methylation and structural stability. Third, as cores condense, a crowding gradient emerges, making peripheral regions more transcriptionally active through enhanced accessibility and crowding-driven interactions. Given the role of histone methylation in core stability, we reasoned that epigenetic inhibitors—those reducing methylation or increasing acetylation—could weaken domain integrity and disrupt chromatin-mediated resistance. In parallel, we considered nuclear ion composition as another contributor to domain stability. Charge neutralization of DNA, mainly driven by multivalent cations, enhances chromatin compaction (45, 46) and correlates with increased heterochromatin levels (47). Thus, compounds that alter the nuclear ionic environment may disrupt electrostatic interactions and weaken domain cores. Based on these mechanisms, our screen focused on two compound classes: epigenetic inhibitors and ion modulators. Altogether, we tested 50 compounds and highlight 9 that significantly reduced $D_{n}$ in Fig. 4*B*. A list of these TPR candidates and their effects on chromatin structure is provided in Table S4.

Epigenetic inhibitors significantly reduced average $D_{n}$ in A2780 cells (Fig. 4*B*; P<0.001 for all compounds except propranolol), but the reductions were modest and did not reach the critical threshold ($D_{n,\text{crit}}$) linked to near-complete cell death. We hypothesized that altering ionic balance might more effectively disrupt packing domains. To test this, we examined celecoxib—a nonsteroidal anti-inflammatory drug (NSAID) known to modulate Na^+^, K^+^, and Ca^2+^ channels (48)—and digoxin, a Na^+^/K^+^-ATPase inhibitor. Both agents significantly reduced $D_{n}$ below the critical threshold (P<0.001; Fig. 4 *B* and *C*). When combined with chemotherapy, celecoxib and digoxin markedly increased apoptosis (P<0.001) but had minimal effects alone (P>0.05; *SI Appendix*, Fig. S8*A*), indicating synergy.

To assess broader applicability, we selected TPRs that induced either strong or modest $D_{n}$ reductions in A2780 cells and tested whether they produced similar effects across other cancer lines. Short (30-min) treatments revealed modest, cell line-specific responses (*SI Appendix*, Fig. S9*A*). For example, VPA was more effective in A2780.m248 than A2780, while digoxin reduced $D_{n}$ in A2780, A2780.m248, and MDA-MB-231, but was less effective in MES-SA and MES-SA/MX2 compared to celecoxib. To test whether these effects were cancer-specific, we also examined noncancerous osteoblasts derived from hMSCs. Celecoxib was the only candidate compound that did not significantly alter $D_{n}$ in these cells (P>0.05; *SI Appendix*, Fig. S8*B*).

We also evaluated whether the most promising TPR candidates enhanced cancer cell death when combined with chemotherapy. Cell viability was quantified as percent inhibition, with 0% indicating no effect and 100% representing complete cell death. Across cancer types, TPR cotreatment increased chemotherapy-induced cell death, with celecoxib and digoxin showing the strongest effects (P<0.01; *SI Appendix*, Fig. S9*B*).

To quantify the relationship between chromatin modulation and treatment efficacy, we developed two indices based on the CDA model that link changes in $D_{n}$ to chemotherapy-induced cell death (*SI Appendix*). The TPR Index compares the relative effects of two TPRs on packing domains:

[10] $$\text{TPR Index}=\frac{\left(\frac{D_{n,\text{TPR1}}+\sigma_{D_{n,\text{TPR1}}}}{D_{n,\text{Ctrl}}+\sigma_{D_{n,\text{Ctrl}}}}\right)}{\left(\frac{D_{n,\text{TPR2}}+\sigma_{D_{n,\text{TPR2}}}}{D_{n,\text{Ctrl}}+\sigma_{D_{n,\text{Ctrl}}}}\right)}$$

Here, $\sigma_{D_{n}}$ is the SD of PDF$\left(D_{n}\right)$, and measurements from two TPR-treated groups and a control are required. The Inhibition Index quantifies the relative enhancement in chemotherapy efficacy conferred by each TPR:

[11] $$\text{Inhibition Index}=\frac{\ln\left(\frac{1-I_{\text{Chemo}}}{1-I_{\text{Chemo+TPR1}}}\right)}{\ln\left(\frac{1-I_{\text{Chemo}}}{1-I_{\text{Chemo+TPR2}}}\right)},$$

where I represents percent inhibition, comparing chemotherapy alone to combination treatment with either TPR. We found an exponential relationship between the TPR Index and Inhibition Index ($R^{2}\;=\;0.98$), showing that greater $D_{n}$ reduction strongly correlates with increased chemotherapy-induced death (Fig. 4*D*). These results support a synergistic mechanism in which TPRs improve chemotherapy beyond the effects of either agent alone by modulating chromatin structure.

Given its selective chromatin modulation in cancer cells and ability to enhance chemotherapy, we selected celecoxib for further investigation. Celecoxib inhibits cyclooxygenase-2 (COX-2), an enzyme frequently overexpressed in cancer that promotes a pro-tumorigenic microenvironment and chemoresistance. To determine whether its synergy with chemotherapy is COX-dependent, we first tested celecoxib in COX-2-deficient HCT116 colorectal cancer cells (49). Notably, celecoxib still decreased $D_{n}$ and synergized with oxaliplatin, indicating that its effects are independent of COX-2 activity (Fig. 4 *E* and *F*). Second, we evaluated two other COX inhibitors that, unlike celecoxib, do not significantly decrease $D_{n}$. Sulindac, a nonselective NSAID that inhibits both COX-1 and COX-2, increased $D_{n}$ and reduced paclitaxel-induced cell death (Fig. 4 *E* and *F*), suggesting a distinct, COX-independent desensitization mechanism. Aspirin, a COX-1-selective NSAID, modestly decreased $D_{n}$ but did not enhance chemotherapy-induced cytotoxicity (Fig. 4 *E* and *F*), further supporting that celecoxib’s effects are unlikely to be mediated solely through COX inhibition.

To directly test whether celecoxib’s effects are mediated through chromatin modulation, we used MgCl_2_ to stabilize packing domains. MgCl_2_ promotes heterochromatin formation (47) and increases $D_{n}$, facilitating the emergence of ovarian cancer stem cells (50). Consistent with these findings, MgCl_2_ pretreatment significantly increased $D_{n}$ (P<0.001; *SI Appendix*, Fig. S10*A*) and reduced the cytotoxic effects of celecoxib plus chemotherapy by 30% (P<0.05; *SI Appendix*, Fig. S10*B*). Together, these results indicate that the ability of celecoxib to enhance chemotherapy efficacy primarily depends on its modulation of packing domains rather than its canonical anti-inflammatory activity.

Next, we quantitatively compared cell death outcomes between combination therapy and chemotherapy alone. To do so, we adapted Eq. **6** from the CDA model to estimate the death probability of TPR cotreated cells ($\Theta_{b}$) based on the probability under chemotherapy alone ($\Theta_{a}$):

[12] $$\Theta_{b}\left(\Theta_{a}\right)=\frac{1}{1+\gamma_{k}^{h_{b}}{\left(\frac{1}{\Theta_{a}}-1\right)}^{\frac{1}{\gamma_{h}}}}$$

By varying $\Theta_{a}$ (for high-$D_{n}$ cells treated with chemotherapy alone) from 0 to 1, we predicted $\Theta_{b}$ for TPR-treated low-$D_{n}$ populations receiving the same chemotherapy.

To test these predictions, we treated A2780 ovarian cancer cells with paclitaxel alone or combined with celecoxib across a range of doses to modulate cytotoxic stress. This approach allowed us to evaluate whether reduced transcriptional plasticity (via lower $D_{n}$) enhances cell death under increasing stress. We quantified cell death using the inhibition rate (IR), derived from percent inhibition (I), and expressed death probability as Θ=1−exp(−IR·t) (data points in Fig. 4*G*; see *SI Appendix*). Using the mean $D_{n}$ in untreated and celecoxib-treated cells (Fig. 4*B*), model fitting estimated an upregulation of $\beta_{a}\approx 3$ (RMSE = 0.16). Among the free parameters, $\beta_{a}$ had the strongest effect on model fit, while baseline expression had minimal impact (*SI Appendix*, Fig. S11). Consistent with CDA predictions, celecoxib-treated cells (lower $D_{n}$) exhibited higher death rates across paclitaxel doses than controls (higher $D_{n}$; Fig. 4*G*). The combination treatment produced a sigmoidal dose–response, underscoring the enhanced efficacy of TPR cotreatment over chemotherapy alone.

To more explicitly represent how chromatin packing properties influence cell death probability, we derived an approximate analytical expression for $\Theta_{b}\left(\Theta_{a}\right)$. This simplification aimed to capture the effects of chromatin structure using a compact form that highlights key dependencies. We made several assumptions: steady-state conditions (t≫τ, where τ is the mRNA decay rate constant), large gene upregulation ($\beta_{a}\gg 1$), weak dependence of $Se_{E,D_{n}}$ on $D_{n}$, and small changes in $D_{n}$ ($\Delta D_{n}=D_{n,b}-D_{n,a}\ll D_{n,a}$). Under these conditions, we obtained:

[13] $${\tilde{\Theta }}_{b}\left(\Theta_{a}\right)={\left[1+{\left(1+Z\right)}^{Q\left(\sqrt{\beta_{a}}-1\right)N_{\text{PD}}^{-Z}}\cdot {\left(\frac{1}{\Theta_{a}}-1\right)}^{N_{\text{PD}}^{-Z}}\right]}^{-1},$$

where $Q=\frac{3}{2\pi }F\left(3\right)\approx 12.6$ and $Z=\Delta D_{n}/D_{n,a}$ (*SI Appendix*). This simplified equation closely fit the experimental data (dashed line, Fig. 4*G*; RMSE = 0.18), revealing that $\Theta_{b}$ is primarily governed by $D_{n,a}$, $\Delta D_{n}$, and $\beta_{a}$. These findings reinforce the conclusion that TPRs potentiate chemotherapy by modulating chromatin packing, offering a path to more targeted therapeutic strategies.

### TPRs Improve Chemotherapeutic Efficacy and Mitigate Tumor Adaptation In Vivo.

**Fig. 5.**
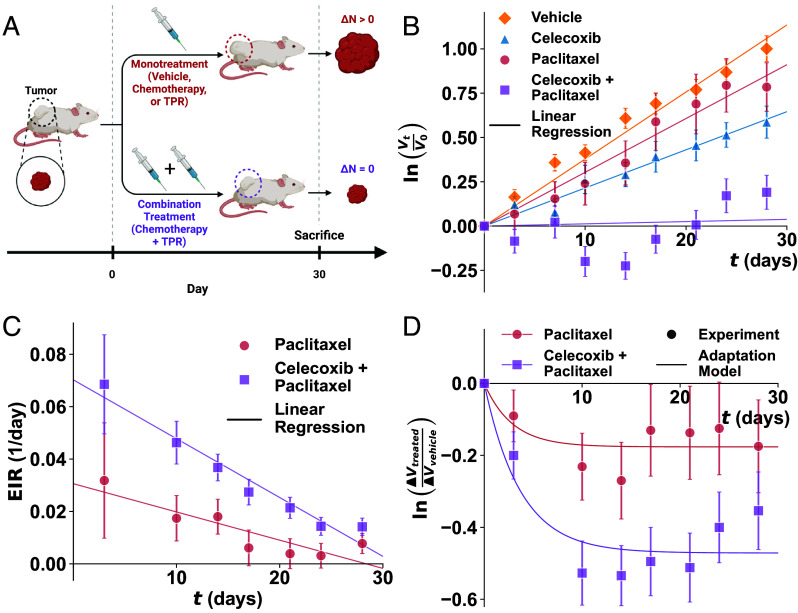
TPR cotreatment enhances chemotherapy efficacy in vivo in an ovarian cancer PDX model. (*A*) Schematic of treatment regimen and expected outcomes. Tumors treated with monotherapy (vehicle, chemotherapy, or TPR alone; red) are expected to continue growing, whereas combination therapy (chemotherapy + TPR; purple) is predicted to suppress tumor growth. (*B*) Normalized tumor volume over 30 d across treatment groups. Points represent mean volume ± SEM; lines indicate linear regression. Cotreatment with celecoxib (25 mg/kg) and paclitaxel (1.7 mg/kg) minimized growth compared to monotherapies or vehicle (DMSO). (*C*) EIR over time for paclitaxel alone (normalized to vehicle) and celecoxib + paclitaxel (normalized to celecoxib). Points show mean ± SEM; lines indicate linear regression fits. (*D*) Normalized tumor growth rate over time. Points represent experimental data (mean ± SEM); solid lines show model fits using the adaptive inhibition equation (Eq. **14**).

To assess whether TPRs can inhibit adaptation to chemotherapy beyond our initial in vitro results, we conducted an in vivo study using a PDX model of ovarian cancer. Celecoxib, the strongest TPR from our screen, was selected for further evaluation. To minimize systemic toxicity while preserving the ability to detect treatment effects, we administered a low dose of paclitaxel. At this dose, we anticipated continued tumor growth in chemotherapy-treated mice. We also expected that vehicle or celecoxib alone would result in progressive tumor growth (Fig. 5*A*, red), consistent with our earlier observations that TPRs have limited antitumor activity alone (*SI Appendix*, Fig. S8*A*). In contrast, our CDA model and in vitro data predicted that combining chemotherapy with celecoxib would significantly reduce tumor growth relative to chemotherapy alone (Fig. 5*A*, purple).

Tumors in the three single-treatment groups—vehicle, celecoxib (25 mg/kg), and paclitaxel (1.7 mg/kg) alone—continued to grow, with the most growth observed in the vehicle group, followed by paclitaxel and then celecoxib (Fig. 5*B*). Celecoxib-treated tumors grew more slowly than those treated with paclitaxel alone, as shown by the blue lines in Fig. 5*B*, likely reflecting cell cycle arrest. Over the 30-d study, tumor volume doubled in both the vehicle and paclitaxel groups. In contrast, the celecoxib-paclitaxel combination restricted tumor growth to just a 20% increase in volume.

As an in vivo PDX model has a more complex systemic environment, we next asked whether the therapeutic benefit of celecoxib arose from its anti-inflammatory activity rather than its modulation of packing domains. Unlike our in vitro experiments, which isolated cancer cell effects, PDX tumors also include stromal and immune cells that shape the tumor microenvironment (TME). Using hematoxylin and eosin (H&E) stained tissue sections, tumor-infiltrating lymphocytes (TILs) were scored on a 0 to 3 scale by two independent pathologists, with higher scores indicating greater immune cell presence in the TME (51–53). Across all treatment groups, most tumors exhibited low TIL scores (0 or 1), indicating minimal immune infiltration (*SI Appendix*, Fig. S12 *A* and *B*, leftmost column). Although celecoxib alone slightly reduced TIL scores relative to control, its combination with paclitaxel did not significantly differ from paclitaxel alone. These findings suggest that reduced lymphocyte recruitment is unlikely to explain the enhanced therapeutic effect of the combination treatment.

To further evaluate celecoxib’s anti-inflammatory activity via the COX-2 pathway, we performed immunohistochemical (IHC) staining for two cytokines commonly regulated by COX-2 signaling: interleukin-6 (IL-6) (54) and tumor necrosis factor alpha (TNF-α) (55). IL-6 is known to be upregulated after paclitaxel treatment and contributes to chemoresistance through the JAK/STAT pathway (56, 57), while TNF-α modulates NF-κB activity. Quantitative analysis of IHC staining showed no significant changes in IL-6 or TNF-α expression with celecoxib treatment, either alone or in combination with paclitaxel (*SI Appendix*, Fig. S12*B*, *Middle* and rightmost columns; and *SI Appendix*, Fig. S12 *C* and *D*). These results suggest that celecoxib’s ability to enhance chemotherapy efficacy in this PDX model is not primarily mediated through modulation of inflammatory cytokines in the TME.

To link in vitro chromatin structure to in vivo outcomes, we used $D_{n}$ distributions from A2780 cells to predict the effective inhibition rate (EIR) per day (*SI Appendix*). This approach allowed us to quantify cumulative cancer cell death over time and assess how TPRs influence chemotherapy efficacy. Based on our CDA model predictions, two trends emerged: 1) EIR would decline over time for both treatment groups, and 2) combination therapy would maintain a consistently higher EIR than chemotherapy alone (*SI Appendix*, Fig. S13*A*). We calculated the experimental EIR using the equation $\text{EIR}=[\ln\left(V_{t,\text{control}}/V_{0,\text{control}}\right)-\ln\left(V_{t,\text{treated}}/V_{0,\text{treated}}\right)]/t$. As predicted by the CDA model, EIR declined over time in both groups, reflecting reduced efficacy at later stages; however, the combination treatment exhibited a higher EIR throughout the experiment (Fig. 5*C*). These findings indicate that TPRs enhance the overall impact of chemotherapy by sustaining its inhibitory effect over time.

To better understand transcriptional plasticity in vivo, we developed a simplified adaptation model that integrates the predictions of the CDA framework while remaining applicable to tumor growth data. This model distinguishes two subpopulations: “unadaptable” cells with lower $D_{n}$, and “adaptable” cells with higher $D_{n}$, which are more likely to survive under treatment. We hypothesize that the rate of tumor adaptation to chemotherapy depends on the initial $D_{n}$ distribution, with higher $D_{n}$ cells adapting more rapidly. As a result, a fraction of the tumor is expected to transition into an adapted state within a critical time window. To model tumor growth without adaptation, we define the relative growth rate as ln(V(t)/V(0))=(p−c)t, where p is the proliferation rate and c is the inhibition rate from chemotherapy. We then extend this to account for adaptation over time:

[14] $$\ln\frac{V(t)}{V(0)}=\left(p-u\right)t-\frac{c-u}{a}[1-\text{exp}\left(-at\right)],$$

where u is the inhibition rate for unadaptable cells, a is the adaptation rate, and t is time since treatment initiation.

As an initial step, we generated tumor volume predictions with the CDA model using $D_{n}$ distributions from A2780 cells. We then fit Eq. **14** to these predictions to extract the chemotherapy inhibition rate (c) and adaptation rate (a). We treated c and a as free parameters, assumed a constant unadaptable inhibition rate (u) across all treatments, and set p=0 since the data were normalized. An initial fit allowing u to vary produced a value of u=0.15, likely reflecting the strong inhibition seen under high-dose in vitro conditions. For chemotherapy alone, the predictions yielded c=1.13 per day and a=2.20 per day (RMSE = 0.009 per day, $R^{2}\;=\;0.99$; *SI Appendix*, Fig. S13*B*). With combination therapy, c is predicted to increase by 60% to 1.81 per day, while a should decrease by 31% to 1.52 per day (RMSE = 0.017 per day, $R^{2}\;=\;0.99$; *SI Appendix*, Fig. S13*B*). These values suggest that TPRs enhance chemotherapeutic efficacy by reducing $D_{n}$ and thereby slowing cellular adaptation.

We next applied the adaptation model to PDX data to test the model’s in vivo predictions. Normalized tumor volumes from paclitaxel-treated (vs. vehicle) and combination-treated mice (vs. celecoxib) were fit using Eq. **14**, with u, c, and a as free parameters. We fixed the proliferation rate at $p=1\times 10^{-10}$ per day due to normalization. Fitting yielded near-zero u values and lower c values than predicted from in vitro data, suggesting the low-dose paclitaxel was weakly inhibitory and readily adapted. Paclitaxel alone had rates of c=0.062 per day and a=0.35 per day (RMSE = 0.049 per day, $R^{2}\;=\;0.60$); with celecoxib cotreatment, c increased to 0.13 per day and a decreased to 0.27 per day (RMSE = 0.069 per day, $R^{2}\;=\;0.85$; Fig. 5*D*). This reflects a 108% increase in inhibition and a 28% drop in adaptation with combination therapy. Together, these results validate that targeted chromatin modulation improves chemotherapy efficacy in vivo by limiting tumor adaptation.

## Discussion

In this study, we introduce the CDA model, a biophysical framework that links chromatin organization to cellular adaptability and chemoevasion via transcriptional plasticity. By incorporating macromolecular crowding from chromatin density within packing domains, the model predicts that chemotherapy-induced cell death depends on the nuclear chromatin scaling parameter, $D_{n}$, at treatment onset (Fig. 1). Consistent with this prediction, we observed treatment-induced increases in $D_{n}$ across multiple cancer types and chemotherapies (Fig. 2), with higher $D_{n}$ associated with cell survival (Fig. 3). Targeting packing domains with TPR agents that lower $D_{n}$ enhanced chemotherapeutic efficacy in vitro (Fig. 4) and celecoxib—the most potent candidate—improved tumor suppression in vivo in an ovarian PDX model (Fig. 5). Our adaptation model further illustrated that cotreatment with celecoxib reduced cellular adaptation and strengthened chemotherapy response compared to paclitaxel alone.

Our probabilistic framework aligns with evidence that cancer cells rapidly adapt, often within timescales shorter than cell division, by activating diverse transcriptional programs (24, 58). Additionally, our results support other findings demonstrating that transient transcriptional states promote chemoresistance (59). Moreover, our emphasis on the average and variability of the transcriptional response influencing cell fate decisions is consistent with studies linking cancer plasticity to transcriptional malleability and heterogeneity (60, 61).

Although our findings provide insight into chromatin-mediated cellular adaptation, several limitations of the CDA model warrant consideration. First, predicting cell fate based on average nuclear $D_{n}$ does not account for variation between individual packing domains. Domains containing critical survival genes may deviate from the nuclear average. Additionally, the spatial positioning of these genes within their domains affects their exposure to crowding. Second, our model simplifies cellular decision-making by categorizing outcomes as either survival or death at fixed checkpoints. In reality, cellular responses are often more nuanced. For instance, chemotherapy can prolong mitotic intervals and alter cell cycle dynamics (62). Furthermore, some cancer cells evade treatment by exiting the cell cycle (63), while others enter a quiescent state in response to damage (64, 65). TPR compounds such as celecoxib can also influence the cell cycle independently of chemotherapy (66). Future versions of the CDA model could incorporate these complex behaviors to improve outcome prediction. Third, our current model focuses on transcriptional responses and does not include the role of translated proteins in cell fate. Protein levels may not correlate directly with gene expression due to posttranscriptional, translational, and posttranslational regulation. Incorporating protein expression rates could help link chromatin structure more accurately to phenotypic outcomes.

Experimentally, we observe that average nuclear chromatin packing scaling ($D_{n}$) increases over time following chemotherapy treatment. This increase may result from two processes: selection for cells with initially higher $D_{n}$, or a direct, time-dependent increase in $D_{n}$ induced by treatment. Given the extent of observed cell death, we predict that chemotherapy does not increase $D_{n}$ rapidly enough to promote survival in most cells. Regardless, in both scenarios, the CDA model predicts that cells with higher $D_{n}$ have a greater likelihood of survival. In addition, we assume the distribution $\text{PDF}(D_{n})$ remains static until cell-fate decisions induce selective survival of high-$D_{n}$ cells, occurring at each interval τ. In future work, single-cell monitoring of $D_{n}$ using PWS could help define a functional form for how chemotherapy dynamically alters $\text{PDF}(D_{n})$. This relationship could then be integrated into the CDA model to more accurately predict cell death probability by accounting for real-time $D_{n}$ changes during treatment.

While our in vitro and in vivo experiments suggest that celecoxib primarily acts through a chromatin-modifying mechanism rather than COX-2 inhibition, we recognize the potential for off-target effects. We emphasize that the goal of this study is not to position celecoxib as the optimal TPR but to motivate future screening efforts. Additionally, we focused on two mechanisms in this study that regulate the formation and maintenance of packing domains: compounds that modulate epigenetics and nuclear ionic balance. Moving forward, we aim to conduct a broader, unbiased screen to expand the pool of effective TPR candidates. This approach will help determine whether TPRs act through shared or distinct biochemical pathways. Such information could guide the development of new therapeutics or repurposing of existing drugs targeting convergent mechanisms. A more diverse TPR toolkit could also support personalized strategies by identifying the most effective compounds for individual patients. Future studies will also evaluate the promising TPRs in immune-competent models to evaluate whether chromatin-modifying mechanisms persist in physiologically relevant settings and to determine how TME interactions influence TPR synergy with chemotherapy.

Discrete biophysical processes, such as loop extrusion and protein-mediated crosslinking regulated by epigenetic factors, drive the formation and maturation of packing domains (20). We propose that TPRs disrupt these processes and rearrange packing domains to create suboptimal crowding conditions within the nucleus, thereby reducing transcriptional upregulation. Celecoxib may destabilize domain cores and make chromatin density more diffuse by altering nuclear ionic composition to increase electrostatic repulsion. In contrast, chemotherapy treatment may promote transcriptional plasticity by altering domain size. Our SMLM data show that chemotherapy leads to the formation of smaller domains, which have greater transcriptional accessibility and may enhance gene expression. However, further studies are needed to identify which molecular mechanisms of packing domain regulation specifically contribute to chemoevasion.

Mapping molecular pathways governing domain dynamics requires high-resolution, single-cell imaging. Fluorescent tagging of key regulatory proteins (e.g., HP1, RNA polymerase II) with time-lapse imaging could directly visualize domain formation, stabilization, and degradation in response to drug treatment. Tracking chemoresistance-associated genes relative to chromatin density in live cells could elucidate how domain architecture influences transcription. Collectively, such experiments could reveal whether TPRs destabilize domain cores through shared mechanisms and whether chemotherapy enhances domain stability via similar pathways.

Ultimately, our insights into chromatin organization pave the way for new cancer therapies that target adaptive resistance mechanisms, offering a promising strategy to improve patient outcomes. Expanding these studies to a wider range of cancer types and treatments, including immunotherapies, could further elucidate the role of chromatin-mediated adaptation in resistance to cancer treatment.

## Materials and Methods

### Cell Culture and Treatments.

Leiomyosarcoma (MES-SA, MES-SA/MX2), breast (MDA-MB-231), and colon (HCT116) cancer cell lines were obtained from ATCC, while ovarian cancer lines (A2780 and A2780.m248) were provided by Dr. Chia-Peng Huang Yang. All lines were cultured in ATCC-recommended media with 10% FBS and routinely tested for *mycoplasma*. Cells were plated on glass-bottom dishes and allowed at least 24 h for adherence. Chemotherapy treatments included paclitaxel (5nM), oxaliplatin (15 µM), 5-fluorouracil (500nM), docetaxel (5nM), and gemcitabine (50nM) for 48 h. TPR compounds, including celecoxib (75 µM), digoxin (150nM), and others, were administered for 30 min. Untreated controls were included for each cell type, and cells were imaged under physiological conditions (5% CO_2_, 37 ^°^C).

### PWS Microscopy.

PWS microscopy was conducted on an inverted microscope (Leica DMIRB) equipped with a Hamamatsu CCD camera and a liquid crystal tunable filter, capturing spectrally resolved images from 500 to 700nm (1nm intervals) (22). Interference spectra were normalized using a reference image and a Butterworth filter was applied to reduce noise. The nuclear average packing domain scaling $D_{n}$ was calculated from spectral variance ($\Sigma^{2}$) through custom MATLAB scripts (32). For additional methodological details, please refer to *SI Appendix*.

### PDX Tumor Models.

The following research protocol was approved by the Institutional Review Board at Northwestern University. High-grade serous ovarian cancer (HGSOC) tissue samples were obtained from chemotherapy-naive patients who underwent surgical resection at Prentice Women’s Hospital between September 2013 and June 2014, with informed consent for tissue acquisition. A cryopreserved, deidentified patient-derived tissue sample (designated OVCA10) was used in its fourth generation (passage 4). Tumor fragments measuring 2 × 2 mm were subcutaneously implanted into the right flank of nonobese diabetic/severe combined immunodeficient gamma (NSG) mice (Jackson Laboratory). Once engrafted tumors reached a volume of 150 to 200 mm^3^, mice were randomized into five treatment groups: celecoxib vehicle control, paclitaxel vehicle control, 25 mg/kg celecoxib, 1.7 mg/kg paclitaxel, and a combination treatment (25 mg/kg celecoxib plus 1.7 mg/kg paclitaxel). Celecoxib or vehicle was administered daily via oral gavage, and paclitaxel or vehicle was administered intraperitoneally twice per week (Mondays and Thursdays). The treatment period lasted 4 wk, during which tumor size and body weight were monitored biweekly. Tumor dimensions were measured using digital calipers, recording the longest axis (length, l) and the perpendicular axis (width, w). Tumor volume (V) was calculated using the formula $V=l\cdot w^{2}/2$. At the end of the 4-wk treatment period, mice were euthanized in accordance with the approved institutional animal care and use protocol (IACUC no. IS00000556), and PDX tumors were harvested and fixed in 10% formalin for downstream analysis.

### Statistical Analysis.

All statistical analyses were performed in Python with a Welch’s *t* test employed for pairwise comparisons between a reference condition and other conditions across groups, including assessments of $D_{n}$, cell viability, and PDX tumor volumes. Statistical significance was denoted as ^∗^*P* < 0.05, ^∗∗^*P* < 0.01, and ^∗∗∗^*P* < 0.001.

## Supplementary Material

Appendix 01 (PDF)

Dataset S01 (CSV)

Dataset S02 (CSV)

Dataset S03 (CSV)

Dataset S04 (CSV)

Dataset S05 (CSV)

Dataset S06 (CSV)

Dataset S07 (CSV)

Dataset S08 (CSV)

## Data Availability

Custom scripts for data analysis and visualization are available at: https://github.com/BackmanLab/CDA_Paper (67). All other data are included in the article and/or supporting information.
