# Supplementary material for: Leveraging chromatin packing domains to target chemoevasion in vivo
Source: Proc Natl Acad Sci U S A. 2025 Jul 22;122(30):e2425319122. doi: 10.1073/pnas.2425319122 (PMC12318189; doi:10.1073/pnas.2425319122)
Supplement: Supplementary file 1 — Appendix 01 (PDF) [file pnas.2425319122.sapp.pdf]

Supporting Information for

Leveraging chromatin packing domains to target chemoevasion *in vivo*

Frederick et al.

Vadim Backman.
E-mail: v-backman@northwestern.edu

This PDF file includes:

Supporting text
Figs. S1 to S13
Tables S1 to S4
SI References

Supporting Information Text

Characterization of Nuclear Chromatin Structure. To examine the relationship between chromatin structure and transcriptional regulation, we employ a polymer physics framework. Chromatin is conceptualized as a complex heteropolymer, where its spatial organization critically influences gene expression. This organization follows scaling laws that relate the spatial extent (r) to the number of DNA base pairs (N) within that extent as $N \sim r^D$, where D is the scaling exponent. Due to its inherent complexity, chromatin exhibits distinct scaling behaviors across different spatial length scales. At the nanoscale level ($\sim$ 80 nm), chromatin organizes into discrete, densely packed regions called packing domains (PDs), which are mass fractal structures associated with transcriptional activity (1). The scaling exponent D_{PD} quantifies the relationship between chromatin mass and volume within individual PDs, while the ensemble average $\langle D_{\mathrm{PD}} \rangle = \frac{1}{T} \sum_{i=1}^{T} D_{\mathrm{PD},i}$, where T represents the total number of packing domains in a nucleus, provides a measure of global domain organization at the nuclear level. Importantly, D_{PD} is distinct from the polymeric Flory exponent, as packing domains exhibit finite scaling behavior from the 10 nm chromatin fiber up to their outer 80 nm radial boundary (2). Experimentally, D_{PD} can be determined for specific nuclear regions using high-resolution techniques such as chromatin scanning transmission electron microscopy (ChromSTEM). To relate chromatin organization to phenotypic outputs at the population level, however, a more global nuclear parameter is required. The nuclear-level packing domain organization can be described by $D_n \approx \langle D_{\mathrm{PD}} \rangle \cdot \mathrm{VF}$, where VF denotes the nuclear volume fraction occupied by domains. Thus, D_n represents the average packing domain scaling exponent weighted by their fractional occupancy within the nucleus. This parameter provides a comprehensive measure of chromatin organization that links structure to transcriptional activity.

The parameter D_n can be experimentally determined by leveraging the power-law form of the spatial autocorrelation function (ACF) for fractal media, $\mathrm{ACF}(r) \propto r^{D_n - 3}$ (3). This relationship yields $D_n \approx D_{\mathrm{ACF}} + \frac{\ln(\mathrm{VF})}{b}$, where D_{ACF} characterizes the shape of the ACF and b is a constant related to the optical and sample properties (4). The parameter D_{ACF} captures interactions between densely packed regions (packing domains) and less dense inter-domain spaces, determining the decay of the ACF. Higher D_{ACF} values indicate slower decay and a more uniform chromatin distribution (2). For live-cell, high-throughput experiments, a proxy for D_{ACF} can be derived using Partial Wave Spectroscopic (PWS) microscopy, which measures spectral variance (Σ^2) related to refractive index fluctuations within a coherence volume of approximately 1 µm^3 (5). The spectral variance Σ^2 is proportional to the Fourier transform of the ACF integrated over a defined Fourier space region ($\Sigma^2 \propto \int \mathcal{F}\{r^{D_{\mathrm{ACF}}-3}\}$) (6). Assuming uncorrelated densities between domains and inter-domain spaces, their Fourier transforms can be summed, resulting in $\Sigma^2 = \Sigma^2_{\mathrm{PD}}\mathrm{VF} + \Sigma^2_{\mathrm{inter}}(1 - \mathrm{VF})$. When $\mathrm{VF} = 1$, chromatin is nearly uniformly packed, resulting in a single ACF. The PWS microscopy signal predominantly reflects contributions from low-frequency components, corresponding to packing domains, since high-frequency components fall outside its detection range (7). Therefore, we assume domains dominate the PWS signal ($\mathrm{VF} \approx 1$), allowing $D_n \approx D_{\mathrm{ACF}}$. This assumption introduces potential inaccuracies when significant inter-domain contributions exist but enables D_n to serve as an experimentally derived proxy for nuclear chromatin organization in subsequent analyses.

Chromatin Packing Macromolecular Crowding (CPMC) Model. The Chromatin Packing Macromolecular Crowding (CPMC) model, previously described in detail (8, 9), examines how chromatin organization influences gene expression through the mechanism of macromolecular crowding. We propose that chromatin regions near the surfaces of packing domains exhibit elevated transcriptional activity due to optimal molecular crowding conditions. The fraction of nuclear chromatin positioned on these transcriptionally favorable surfaces is quantified by the exposure ratio (ER). This ratio follows a fractal scaling relationship $\mathrm{ER} \approx (N_{\mathrm{PD}}/A_v)^{-1/D_{\mathrm{PD}}}$, where N_{PD} represents the genomic size of a packing domain (in base pairs), and A_v denotes the volume packing efficiency within the domain (2). The parameter A_v measures how efficiently the 10 nm chromatin fiber is packed in a domain, with a value of 1 indicating fully fractal packing behavior.

The CPMC model aims to predict transcription patterns on a genome-wide scale by linking local gene expression rates to broader nuclear organization. The local expression rate, ε, reflects the mRNA production rate within the interaction volume around a single gene, shaped by the local chromatin environment. In contrast, the ensemble expression rate, E, reflects the transcriptional output of a group of genes subjected to similar local and nuclear-scale conditions. This rate is calculated by weighting the average local expression rate, $\bar{\varepsilon}$, by the exposure ratio, ER: $E = \bar{\varepsilon} \cdot \mathrm{ER}$. To approximate ε, we employ the Macromolecular Crowding (MC) model described by Matsuda et al. (10) and Shim et al. (11).

Impact of Crowding on Transcription. The MC model simulates mRNA synthesis to investigate the impact of crowding on the diffusion and binding of transcriptional reactants, such as transcription factors (TFs) and RNA polymerase II (Pol II). It focuses on the transcriptional interaction volume surrounding a single gene, with the fraction of space occupied by crowders denoted as ϕ_{in}. The synthesis of mRNA is modeled using differential equations with inputs comprising protein concentrations, DNA association/dissociation rates, and binding affinities, collectively represented as $\vec{m}$ (the molecular regulators of transcription). For this study, $\vec{m}$ values remain constant except for the concentrations of transcriptional reactants, where high concentrations simulate genes with high expression (associated with frontloading prior to stress) and low concentrations represent genes relying on plasticity during stress responses. Detailed methodologies are outlined in the "Macromolecular Crowding Model" section, with input parameters summarized in Table S1. Results demonstrate that within the interaction volume, mRNA production responds non-monotonically to crowding, balancing enhanced reactant binding to DNA with reduced diffusive mobility (10, 11). Transcription is maximized at an intermediate crowding level, $\phi_{\mathrm{in,max}}$, where efficiency peaks and is determined by $\vec{m}$ (Fig. 1B). This non-monotonic relationship, characterized by a distinct optimal peak, informs the analytical approximation of the local gene expression rate ε, capturing both the peak and its curvature in a computationally efficient manner.

Approximation of Average Gene Expression Rate. The exact form of the average gene expression rate is determined by:

$$\bar{\varepsilon}(\vec{m}) = \int \varepsilon(\vec{m}, \phi_{\text{in}}) f(\phi_{\text{in}}) d\phi_{\text{in}} \quad [1]$$

To calculate this, we first use our previously published model of transcription to determine the relationship between crowding ϕ_{in} and transcription for a given molecular environment (10, 11). This provides us with a functional relationship as well as $\phi_{\text{in,max}}$, the crowding value at which transcription is maximized.

Next, we use a Taylor expansion around $\phi_{\text{in,max}}$ to estimate the gene expression rate (8, 9):

$$\bar{\varepsilon}(\vec{m}) \approx \varepsilon(\vec{m}, \phi_{\text{in,max}}) + \frac{1}{2}\sigma^2_{\phi_{\text{in}}} \frac{\partial^2 \varepsilon(\vec{m}, \phi_{\text{in}})}{\partial \phi^2_{\text{in}}} (\phi_{\text{in}} - \phi_{\text{in,max}})^2 + \ldots \quad [2]$$

Since the first derivative goes to zero at $\phi_{\text{in,max}}$, the second derivative dominates near the peak.

To calculate $\sigma^2_{\phi_{\text{in}}}$ (the spatial variation in crowding within packing domains), we use the ACF of ϕ_{in} (8, 9):

$$\sigma^2_{\phi_{\text{in}}} \approx \left(\frac{3}{D_{\text{ACF}}}\right) \left(\frac{r_{\text{min}}}{r_{\text{in}}}\right)^{3-D_{\text{ACF}}} \sigma^2_o \quad [3]$$

$$\sigma^2_o = \langle \phi_c \rangle (1 - \langle \phi_c \rangle)(1 - \langle \phi_m \rangle)^2 \quad [4]$$

where $D_{\text{ACF}} \approx D_n$ (see SI section "Characterization of Nuclear Chromatin Structure"), $r_{\text{min}} = 1$ nm is the radius of a DNA base pair, r_{in} is the interaction volume radius, σ^2_o is the overall variance in crowding, $\langle \phi_c \rangle$ and $\langle \phi_m \rangle$ represent average crowding contributions from chromatin and other mobile crowders, respectively. The radius of the interaction volume is given by $r_{\text{in}} = r_{\text{min,in}} + r_{\text{min}} \left(\frac{L}{\langle A_v \rangle}\right)^{1/D_n}$, where $r_{\text{min,in}} = 15$ nm represents the base pair interaction volume, and $L = 6$ kbp is the gene length.

We estimate the second derivative as $\frac{\partial^2 \varepsilon(\vec{m}, \phi_{\text{in}})}{\partial \phi^2_{\text{in}}}|_{\phi_{\text{in,max}}} \approx -\sqrt{\kappa / \bar{\varepsilon}(\vec{m})}$. Here, $\kappa \approx 67.5$ nM/s is determined by fitting the curve that results from plotting $\varepsilon(\vec{m}, \phi_{\text{in,max}})$ against $\frac{\partial^2 \varepsilon(\vec{m}, \phi_{\text{in}})}{\partial \phi^2_{\text{in}}}|_{\phi_{\text{in,max}}}$, as shown in Fig. S1A. Additionally, averaging $\bar{\varepsilon}(\vec{m})$ over $\vec{m}$ results in $\langle \bar{\varepsilon} \rangle \approx 14.6$ nM/s (Fig. S1B), which we use to create a vector of normalized expression rates $-6 < \ln(E/\overline{E}) < 3$ (corresponding to 0.036 nM/s $< \bar{\varepsilon} <$ 292.8 nM/s) in further calculations.

Analysis of Gene Expression Sensitivity. Here, we conduct a comparative analysis of gene expression sensitivities to key properties of packing domains. For simplicity, D_n, N_n, and $A_{v,n}$ refer to the average nuclear packing domain properties: packing scaling coefficient, genomic domain size, and packing efficiency, respectively, averaged over all domains in the nucleus. We define the sensitivity of gene expression, Se, as a combination of sensitivities to the average local expression rate ($\bar{\varepsilon}$) and the exposure ratio (ER), denoted $Se_{\bar{\varepsilon}}$ and Se_{ER}, respectively. As in the previous section, these definitions assume a Taylor expansion of the gene expression rate to derive closed-form sensitivities.

For D_n, the sensitivity is expressed as $Se(D_n) = Se_{\bar{\varepsilon}}(D_n) + Se_{\text{ER}}(D_n)$, where:

$$Se_{\bar{\varepsilon}}(D_n) = \frac{\partial \ln \bar{\varepsilon}}{\partial \ln D_n} \approx -G(\bar{\varepsilon}) \left[D_n \ln\left(\frac{r_{\text{in}}}{r_{\text{min}}}\right) + \frac{3 - D_n}{D_n} \frac{r_{\text{min}}}{r_{\text{in}}} \left(\frac{L}{\langle A_v \rangle}\right)^{1/D_n} \ln\left(\frac{L}{\langle A_v \rangle}\right) \right] + \frac{3}{D_n} \ln\left(\frac{\langle N_{\text{PD}} \rangle}{\langle A_v \rangle}\right) \left(\frac{1 - 2\phi_c}{1 - \phi_c}\right) \quad [5]$$

$$Se_{\text{ER}}(D_n) = \frac{\partial \ln \text{ER}}{\partial \ln D_n} \approx \frac{1}{D_n} \ln\left(\frac{\langle N_{\text{PD}} \rangle}{\langle A_v \rangle}\right) \quad [6]$$

Here, $\phi_c = \langle A_v \rangle (\langle N_{\text{PD}} \rangle / \langle A_v \rangle)^{1-3/D_n}$, and $G(\bar{\varepsilon})$ is the dimensionless function $G(\bar{\varepsilon}) = \frac{\kappa}{8\bar{\varepsilon}} (\sigma^2_{\phi_{\text{in}}})^2 \left(1 + \sqrt{1 + \frac{16}{(\sigma^2_{\phi_{\text{in}}})^2} \frac{\bar{\varepsilon}}{\kappa}}\right)$ For most physiological scenarios, where $16/\sigma^2_{\phi_{\text{in}}} \gg 1$, $G(\bar{\varepsilon})$ simplifies to $G(\bar{\varepsilon}) \approx \frac{1}{2}\sigma^2_{\phi_{\text{in}}} \sqrt{\kappa/\bar{\varepsilon}}$.

For the average genomic domain size, $\langle N_{\text{PD}} \rangle$, the sensitivity is $Se(\langle N_{\text{PD}} \rangle) = Se_{\bar{\varepsilon}}(\langle N_{\text{PD}} \rangle) + Se_{\text{ER}}(\langle N_{\text{PD}} \rangle)$, with:

$$Se_{\bar{\varepsilon}}(\langle N_{\text{PD}} \rangle) = \frac{\partial \ln \bar{\varepsilon}}{\partial \ln \langle N_{\text{PD}} \rangle} \approx -G(\bar{\varepsilon}) \left(1 - \frac{3}{D_n}\right) \left(\frac{1 - 2\phi_c}{1 - \phi_c}\right) \quad [7]$$

$$Se_{\text{ER}}(\langle N_{\text{PD}} \rangle) = \frac{\partial \ln \text{ER}}{\partial \ln \langle N_{\text{PD}} \rangle} \approx -\frac{1}{D_n} \quad [8]$$

For the average packing efficiency, $\langle A_v \rangle$, the sensitivity is $Se(\langle A_v \rangle) = Se_{\bar{\varepsilon}}(\langle A_v \rangle) + Se_{\text{ER}}(\langle A_v \rangle)$:

$$Se_{\bar{\varepsilon}}(\langle A_v \rangle) = \frac{\partial \ln \bar{\varepsilon}}{\partial \ln \langle A_v \rangle} \approx -G(\bar{\varepsilon}) \left(\frac{3}{D_n} - 1\right) \left(\frac{1 - 2\phi_c}{1 - \phi_c} + \frac{\langle A_v \rangle}{L} \frac{r_{\text{min}}}{r_{\text{in}}}\right) \quad [9]$$

$$Se_{\text{ER}}(\langle A_v \rangle) = \frac{\partial \ln \text{ER}}{\partial \ln \langle A_v \rangle} \approx \frac{1}{D_n} \quad [10]$$

Using this analytical framework, we evaluated the influence of packing domain properties (D_n, N_n, and $A_{v,n}$) on gene expression. Specifically, we determined the sensitivity of gene expression to each parameter across different initial relative expression rates, defined by molecular factors as $\ln(E/\overline{E})$. Negative values of $\ln(E/\overline{E})$ indicate genes with initially low pre-stimulus expression, while positive values correspond to genes with initially high expression levels and optimal molecular conditions.

Sensitivities for the 25th, 50th, and 75th percentiles of each parameter were determined using ChromSTEM data from HCT116 cells, providing biologically realistic parameter ranges. Overall, we found that gene expression sensitivity to packing domain scaling ($Se(D_n)$) was significantly greater than sensitivity to N_n or $A_{v,n}$, with differences spanning 1–2 orders of magnitude (Fig. S2).

These results highlight the critical role of D_n in regulating cellular adaptability and stress response, providing a strong rationale for focusing on this parameter in subsequent analyses. In subsequent analyses, we adopt average values of $N_{\text{PD}} = 380$ kbp and $A_v = 0.6$.

Chromatin-Dependent Adaptability (CDA) Model. The Chromatin-Dependent Adaptability (CDA) model builds upon established links between gene expression and chromatin organization to predict cell survival probability under stress. This model proposes that a population's survival likelihood in the face of cytotoxic stress depends on the average nuclear packing domain organization, D_n, which governs transcriptional responses. Crucially, the CDA model does not imply that individual cells universally survive all stressors through targeted gene regulation. Instead, it evaluates survival statistically, emphasizing how the average pre-stress chromatin organization influences the crowding conditions of all genes within the cell.

Elaboration of Θ. The change in transcript levels for the gene(s) of interest is given by the ratio $x = N_2/N_1$, where N_1 and N_2 represent the number of mRNA transcripts before and after stress exposure, respectively. We hypothesize that the probability density function (PDF) of the transcript quantity follows a log-normal distribution:

$$\text{PDF}(x) \approx \frac{1}{s\sqrt{2\pi x}} \mathrm{e}^{-\frac{\ln(x/m)^2}{2s^2}} \quad [11]$$

where m is the median of the distribution, and s is the shape parameter. The parameter s can also be interpreted as the standard deviation of $\ln(x)$, representing the spread of transcript number. When $s \ll 1$, $\ln(m) \approx \mu$, where μ is the mean, and s approximates the coefficient of variation (COV) of transcript upregulation.

We postulate that a cell's survival after exposure to a cytotoxic stressor, within a critical time window, depends on the upregulation of specific stress-response genes exceeding a threshold, x_{crit}. This leads to the assumption that the probability of survival, as a function of x, can be approximated by a step function: the cell survives if $x > x_{\text{crit}}$, and it dies if $x < x_{\text{crit}}$. The probability of cell death, Θ, is thus defined by the cumulative distribution function (CDF) at the threshold x_{crit}:

$$\Theta(x_{\text{crit}}) = \text{CDF}(x_{\text{crit}}) = \frac{1}{2}\text{erfc}\left(\frac{\ln(m/x_{\text{crit}})}{\sqrt{2}s}\right) \quad [12]$$

where the complementary error function is defined as $\text{erfc}(u) = 1 - \text{erf}(u) = \frac{2}{\sqrt{\pi}}\int_u^\infty e^{-v^2} dv$. The use of erfc naturally arises from the log-normal distribution, representing the tail probability beyond x_{crit}.

To facilitate both numerical and analytical computation, we approximate the log-normal CDF with a Hill equation, providing a more tractable form:

$$\Theta(x_{\text{crit}}) \approx \frac{1}{1 + (k/x_{\text{crit}})^h} \quad [13]$$

where $k \approx m$ is the malleability of the response, representing the median level of transcript change required for survival, and $h \approx 3/(s\sqrt{\pi})$ is the Hill coefficient, inversely related to the COV or heterogeneity of the response. This approximation captures the sigmoidal nature of the cell survival curve as a function of x_{crit}, facilitating computation while retaining the essential features of the original log-normal distribution.

Next, we generalize this formulation to compare the survival probabilities of two cells, labeled a and b. As depicted in the main text (see Fig. 1), we compare the upregulation of cell a with that of cell b, and calculate the death probability of cell b as:

$$\Theta_b(x_{\text{crit}}) \approx \frac{1}{1 + (k_a\gamma_k/x_{\text{crit}})^{\frac{h_a}{\gamma_h}}} \quad [14]$$

where $\gamma_k = k_b/k_a$ and $\gamma_h = h_a/h_b$. Here, γ_k represents the ratio of transcript upregulation between cells b and a, and γ_h represents the ratio of the COVs of transcript upregulation between the two cells.

Determining the exact threshold x_{crit} experimentally for all relevant pro-survival genes in cell b can be challenging. An alternative approach is to calculate the death probability of cell b by using the known death probability for cell a to solve for x_{crit}. Rewriting Eq. 13, we obtain:

$$\Theta_b(\Theta_a) = \frac{1}{1 + \gamma_k^{h_b}(\frac{1}{\Theta_a} - 1)^{\frac{1}{\gamma_h}}} \quad [15]$$

This formulation directly links Θ_b to Θ_a, allowing for the calculation of the death probability of cell b based on the characteristics of cell a. This equation simplifies comparative analyses between different cell populations.

Incorporating Temporal Dynamics. The CPMC model's versatility allows application to both steady-state and non-steady-state conditions. For steady-state, the number of mRNA transcripts (N) is not time-dependent, i.e., $\frac{\partial N}{\partial t} = 0$. In this scenario, CPMC predicts N by determining the expression rate E and using the relation $E = N\nu$ for the mRNA degradation rate ν (10). However, many biological processes, including responses to chemotherapeutic agents, occur under non-steady-state conditions.

To address these conditions, we developed an extended formalism. Let a cell population be exposed to a stressor at time $t = 0$. The number of transcripts at time t is determined by:

$$\frac{\partial N}{\partial t} = \frac{\partial E}{\partial t}(1 - \mathrm{e}^{-t/\tau}) \qquad [16]$$

where τ is the mRNA elimination time constant, calculated from the half-life of mRNA, $\tau_{1/2}$, as $\tau = \frac{1}{\ln 2}\tau_{1/2}$. Based on existing literature, we adopt $\tau_{1/2} = 10$ hours (12). This differential equation captures the delay in transcript accumulation as the system transitions from initial exposure to a new steady state.

We approximate $\frac{\partial N}{\partial t} \approx \frac{N_2(t)-N_1}{N_1}$ as the relative change in transcripts and $\frac{\partial E}{\partial t} \approx \frac{E_2(t)-E_1}{E_1}$ as the relative change in mRNA production rate. Both $N_2(t)$ and $E_2(t)$ are time-dependent after stress exposure, reflecting the dynamic nature of the cellular response. Defining $\beta = \frac{E_2(t)}{E_1}$ and $k = \frac{N_2(t)}{N_1}$, we describe transcriptional malleability with:

$$k(t) = 1 + (\beta - 1)\,(1 - \mathrm{e}^{-t/\tau}) \qquad [17]$$

such that $k(t)$ evolves over time, approaching β as $t \to \infty$, indicating eventual transcript upregulation.

If we know β_a, the average upregulation of the expression rate for cell a, we can predict β_b using:

$$\delta = \frac{\beta_b}{\beta_a} = \exp\left[\int_{D_{n,a}}^{D_{n,b}} \frac{Se_2(D'_n) - Se_1(D'_n)}{D'_n} dD'_n\right] \qquad [18]$$

with $E_{2,a} = \beta_a E_{1,a}$ to link differences in gene expression to chromatin structure via D_n. We compute $k_b = k_a\gamma_k$ by substituting $\beta_b = \delta\beta_a$ into Eq. 17, yielding the transcriptional malleability for cell b.

To account for evolving heterogeneity in gene expression over time, we developed an equation integrating both initial variability and stress-induced changes:

$$s(t) = COV\left[x = \frac{N_2(t)}{N_1}\right] = \beta\frac{1 - \mathrm{e}^{-t/\tau}}{k(t)}\sqrt{COV\left[E_1\right]^2 + COV\left[E_2\right]^2} \qquad [19]$$

where $COV[E_1] = \sqrt{2}G(\bar{\varepsilon}_i)$ and $\bar{\varepsilon}_i$ is the expression rate of a given gene per unit of DNA pre- ($i = 1$) and post- ($i = 2$) stimulus. Assuming transcriptional heterogeneity is negligible prior to treatment ($COV[E_2] \gg COV[E_1]$), we express the time-dependent heterogeneity for cells a and b as:

$$s_a(t) = \beta_a\frac{1 - \mathrm{e}^{-t/\tau}}{k_a(t)}\sqrt{2}G\left(\bar{\varepsilon}_{2,a}\right) \qquad [20]$$

$$s_b(t) = \beta_b\frac{1 - \mathrm{e}^{-t/\tau}}{k_b(t)}\sqrt{2}G\left(\bar{\varepsilon}_{2,b}\right) \qquad [21]$$

where $\bar{\varepsilon}_{2,a} = \beta_a\bar{\varepsilon}_{1,a}$ and $\bar{\varepsilon}_{2,b} = \beta_b\bar{\varepsilon}_{1,b}$. These expressions indicate how heterogeneity evolves in each cell over time, modulated by changes in gene expression rates and transcript numbers.

If $\bar{\varepsilon}_{1,a}$ is known, the expression rate $\bar{\varepsilon}_{1,b}$ can be found using the relation $\gamma_{\bar{\varepsilon}_i} = \bar{\varepsilon}_{i,b}/\bar{\varepsilon}_{i,a}$. Here, $\gamma\bar{\varepsilon}_i$ represents the average change in expression rate for cell a compared to cell b before ($i = 1$) or after ($i = 2$) treatment with a cytotoxic stressor, calculated using $Se_{\bar{\varepsilon}}(D_n)$:

$$\gamma_{\bar{\varepsilon}} = \frac{\bar{\varepsilon}_b}{\bar{\varepsilon}_a} = \frac{E_b}{E_a}\frac{ER\left(D_{n,a}\right)}{ER\left(D_{n,b}\right)} = \left(\frac{N_{\mathrm{PD}}}{A_v}\right)^{\frac{1}{D_{n,b}} - \frac{1}{D_{n,a}}} \exp\left(\int_{D_{n,a}}^{D_{n,b}} Se_{\bar{\varepsilon}_a}(D'_n)\frac{dD'_n}{D'_n}\right) \qquad [22]$$

Thus, we can determine the expression rate in cell b after treatment, $\bar{\varepsilon}_{2,b}$, using:

$$\bar{\varepsilon}_{2,b} = \bar{\varepsilon}_{1,a}\beta_a\gamma_{\bar{\varepsilon}_2} = \bar{\varepsilon}_{1,a}\delta\beta_a\frac{\mathrm{ER}_a}{\mathrm{ER}_b} = \bar{\varepsilon}_{1,a}\delta\beta_a\left(\frac{N_{\mathrm{PD}}}{A_v}\right)^{\frac{1}{D_{n,b}} - \frac{1}{D_{n,a}}} \qquad [23]$$

where ER_a and ER_b represent the exposure ratio in cells a and b, respectively, inversely related to chromatin packing density. The ER ratio $\frac{\mathrm{ER}_a}{\mathrm{ER}_b}$ is expressed as a function of N_{PD}, A_v, and D_n. Substituting Eqs. 20, 21, 22, and 23 into Eq. 19, we calculate the heterogeneity ratio, $h_b = 3/(s_b\sqrt{\pi}) = h_a/\gamma_h$, necessary for computing the death probability $\Theta(x_{\mathrm{crit}})$ in Eq. 14.

Evaluating Model Parameters. To predict the cell death probability Θ, the CDA model requires estimation of several key parameters: (1) the gene upregulation factor β; (2) the survival threshold x_{crit}; (3) the relative initial expression of the upregulated gene(s) $\ln(E_1/\overline{E}_1)$; (4) the critical decision time point t_{crit}. A list of values for the other relevant parameters can be found in Table S2. As a function of D_n, Θ follows a sigmoidal curve where cells with low D_n have a close to 100% probability of death when they encounter a stressor and cells with high D_n having close to 0% probability (Figs. 1D-E and S3) The two main parameters that are key to the trends for Θ are β and x_{crit}, which shift the probability of death for high D_n cells and the inflection point of the sigmoidal curve, respectively (Figs. 1D-E). Two other parameters that have less of an impact on model predictions are $\ln(E_1/\overline{E}_1)$ and t_{crit}.

The critical timepoint for the cell death decision, t_{crit}, is an important parameter in the CDA model. Previous studies have shown that cells typically exhibit signs of apoptosis 5-10 hours post-chemotherapy treatment, with apoptosis induction varying significantly depending on the dosage (13–15). Here, we use $t_{\text{crit}} = 7$ hours. Increasing t_{crit} changes the sigmoidal curve so that cells with lower D_n have a lower probability of death, as illustrated in Fig. S3, which shows the relationship between chromatin packing scaling (D_n) and cell death probability for different t_{crit} values.

Incorporating Population Distribution of D_n. Our experimental results reveal that cell populations exhibit heterogeneous responses to chemotherapy, characterized by a distribution of D_n. Cells with extreme values of D_n are particularly sensitive to chemotherapy, necessitating a model that incorporates this variability. To represent this distribution, we define a probability distribution function (PDF) that spans a range of D_n values, allowing us to capture the full spectrum of cell responses.

We introduce n_τ as the number of cell doubling intervals since exposure to the stressor, with τ_2 representing the characteristic cell doubling time. Cells decide whether to undergo apoptosis or division based on a comparison between the critical time for apoptosis, T_{crit}, and the doubling time, τ_2. Specifically, when $T_{\text{crit}}<\tau_2$, the decision time after n_τ doublings is given by $t_{n_\tau} = n_\tau \cdot \tau_2$.

The probability of cell survival, P_s, is inversely related to the probability of cell death, Θ, through the relationship $P_s = 1-\Theta$. For a population with a distribution of D_n values, the overall survival probability is determined by integrating over the population distribution:

$$P_s(n_\tau) = \int \text{PDF}(D_n)\big[1 - \Theta(D_n)\big]^{n_\tau}\, dD_n \qquad [24]$$

This formulation highlights how the survival probability declines with successive cell divisions, with the rate of decline modulated by each cell's specific D_n value.

To determine the population-averaged D_n, $\langle D_n \rangle$, we compute a weighted average of D_n, where the weighting is determined by the survival probability:

$$\langle D_n(t_{n_\tau}) \rangle = \frac{\int D_n \cdot \text{PDF}(D_n)[1 - \Theta(D_n)]^{n_\tau}\, dD_n}{\int \text{PDF}(D_n)[1 - \Theta(D_n)]^{n_\tau}\, dD_n} \qquad [25]$$

This expression provides a time-dependent measure of $\langle D_n(t_{n_\tau}) \rangle$, accounting for how cell survival evolves through repeated divisions under stress.

Next, we introduce the effective inhibition rate (EIR), which quantifies the cumulative effect of chemotherapy on the cell population. The EIR describes the rate at which the survival probability decays over time, modeled as an exponential decay with $P_s = \exp(-t_{n_\tau} \cdot \text{EIR})$. For a homogeneous population where all cells have the same D_n value (i.e., $\text{PDF}(D_n) = \delta(D_n - D_{n,a})$), we derive the following expression for the EIR:

$$\text{EIR}(t_{n_\tau}) = \frac{1}{t_{n_\tau}} \ln\left(\frac{1}{\big[1 - \Theta(D_{n,a})\big]^{n_\tau}}\right) \qquad [26]$$

To assess the rate of inhibition per doubling interval, we define the incremental inhibition rate (IR):

$$\text{IR}(n_\tau) = \frac{1}{t_1} \ln\left(\frac{P_s(n_\tau)}{P_s(n_\tau + 1)}\right) \qquad [27]$$

where $t_1 = \tau_2$. In the case of a homogeneous population, where $\text{PDF}(D_n) = \delta(D_n - D_{n,a})$, we find that $\text{IR}(n_\tau) = \text{EIR}(n_\tau)$.

Assuming that cell proliferation rates are independent of D_n, the total number of cells at time t without chemotherapy follows an exponential growth law such that $N(t) = N(0)\exp(t\ln(2)/\tau_2)$. When chemotherapy is applied, the growth rate is modified by the inhibition rate, yielding:

$$\frac{N(t)}{N(0)} = \exp\left(\frac{t\ln(2)}{\tau_2} - t \cdot \text{IR}(t)\right) \qquad [28]$$

Experimental measurements of P_s must therefore be normalized by the expected number of cells at time t in the absence of stress, $\exp(t\ln(2)/\tau_2)$, to account for natural cell proliferation.

Finally, we express the full inhibition rate as a function of time:

$$\mathrm{IR}(t) = \frac{1}{P_s(t)}\frac{\partial P_s(t)}{\partial t} = \frac{1}{t_1}\int \mathrm{PDF}(D_n)\big[1-\Theta(D_n)\big]^{t/t_1}\ln\big[1-\Theta(D_n)\big]dD_n \qquad [29]$$

This equation captures the cumulative effect of stress on the population, incorporating the heterogeneity in D_n. By integrating the survival probabilities across the distribution of D_n values, this model provides a more comprehensive understanding of how chemotherapy influences the population dynamics over time.

Estimation of Cell Division Induced Drift in Population D_n. To accurately predict cell death probability as a function of D_n, we developed a comprehensive approach that models the effects of both time and cell division on chromatin packing domains. In theory, if D_n remained the same in daughter cells after cell division, Θ could be directly estimated simply by looking at the difference in the PDFs of D_n at two time points, as long as the growth rate is known. However, we have previously seen that there is some drift in D_n when daughter cells are compared to the parent cell (1), indicating the need to take cell division into account when estimating experimental Θ. Therefore, we can define the change in the number of cells using the equation:

$$\frac{dN(D_n,t)}{dt} = \frac{\ln 2}{\tau_2}\int N(D_n',t)\cdot f(D_n-D_n')\;dD_n' - N(D_n,t)\cdot \mathrm{IR}(D_n,t) \qquad [30]$$

where N is the cell number as a function of D_n and time t, τ_2 is the doubling time, $f(D_n - D_n')$ is the probability of a daughter cell having D_n if the mother cell had D_n', and IR is the inhibition rate for a cell with D_n at time t. While N, D_n, and IR are relatively simple to determine experimentally, $f(D_n - D_n')$ requires more complex estimation.

Definition of Drift in D_n. To isolate the effect of cell division on D_n, we first determined all of the separate factors that impact population drift, including (1) intrinsic variation within a cluster, (2) normal temporal changes, and (3) the impact of cell division. We start by representing the overall drift as a normally distributed PDF $f(\Delta D_n)$ which is the change in D_n between two time points (before and after cell division) with a mean of 0 and a variance of $\mathrm{Var}[\Delta D_n]$. To find $\mathrm{Var}[\Delta D_n]$, we first start by defining the D_n of a cluster of cells before $(D_{n,1})$ and after $(D_{n,2})$ cell division as:

$$D_{n,1} = \frac{1}{N_{\mathrm{non}}+N_{\mathrm{div}}}\left(\sum_{i=1}^{N_{\mathrm{non}}} D_{n,1i} + \sum_{i=N_{\mathrm{non}}+1}^{N_{\mathrm{non}}+N_{\mathrm{div}}} D_{n,1i}\right) \qquad [31]$$

$$D_{n,2} = \frac{1}{N_{\mathrm{non}}+2N_{\mathrm{div}}}\left(\sum_{i=1}^{N_{\mathrm{non}}} D_{n,2i} + \sum_{i=N_{\mathrm{non}}+1}^{N_{\mathrm{non}}+N_{\mathrm{div}}} D_{n,2i} + \sum_{i=N_{\mathrm{non}}+N_{\mathrm{div}}+1}^{N_{\mathrm{non}}+2N_{\mathrm{div}}} D_{n,2i}\right) \qquad [32]$$

where N_{non} is the number of non-dividing cells, N_{div} is the number of dividing cells, $D_{n,1i}$ is the D_n of cell i in a cluster before division, $D_{n,2i}$ is the D_n of cell i in the same cluster after division.

The D_n after cell division $(D_{n,2i})$ for dividing cells within a cluster $(i{>}N_{\mathrm{non}})$ is:

$$D_{n,2i} = \overline{D}_{n,1i} + \delta D_{n,i} + \delta D^t_{n,2i} \qquad [33]$$

where $\overline{D}_{n,1i}$ represents the time-averaged D_n of the parent cell, $\delta D_{n,i}$ is the spread in D_n due to division, and $\delta D^t_{n,2i}$ denotes the temporal fluctuation of D_n. Additionally, $\forall ij$, $D_{n,ji} = \overline{D}_{n,ji} + \delta D^t_{n,ji}$, as we can find the D_n of any cell within the cluster before or after cell division given the parent cell D_n and the temporal drift.

Combining Eqs. 31, 32, and 33, we can calculate $\Delta D_n = D_{n,2} - D_{n,1}$ as:

$$\Delta D_n = \sum_{i=1}^{N_{\mathrm{non}}} \overline{D}_{n,1i}\left(\frac{1}{N_2}-\frac{1}{N_1}\right) + \sum_{i=1}^{N_{\mathrm{non}}} \delta D^t_{n,2i}\frac{1}{N_2} - \sum_{i=1}^{N_{\mathrm{non}}} \delta D^t_{n,1i}\frac{1}{N_1} + 2\sum_{i=N_{\mathrm{non}}+1}^{N_{\mathrm{non}}+N_{\mathrm{div}}} \overline{D}_{n,1i}\left(\frac{1}{N_2}-\frac{1}{2N_1}\right) \qquad [34]$$

$$+ \sum_{i=N_{\mathrm{non}}+1}^{N_2} \delta D_{n,i}\frac{1}{N_2} + \sum_{i=N_{\mathrm{non}}+1}^{N_2} \delta D^t_{n,i}\frac{1}{N_2} - \sum_{i=N_{\mathrm{non}}+1}^{N_1} \delta D^t_{n,i}\frac{1}{N_1} \qquad [35]$$

where $N_1 = N_{\mathrm{non}} + N_{\mathrm{div}}$ is the number of cells before cell division occurs and $N_2 = N_{\mathrm{non}} + 2N_{\mathrm{div}}$ is the number after.

From Eq. 34, we can now determine $\mathrm{Var}[\Delta D_n]$:

$$\mathrm{Var}[\Delta D_n] = \mathrm{Var}[\overline{D}_n]\frac{N_{\mathrm{non}}N_{\mathrm{div}}}{N_1 N_2^2} + \mathrm{Var}[\delta D^t_n]\left(\frac{1}{N_2}+\frac{1}{N_1}\right) + \mathrm{Var}[\delta D_n]\frac{2N_{\mathrm{div}}}{N_2^2} \qquad [36]$$

where $\mathrm{Var}[\overline{D}_n]$ is the variance within a cluster, $\mathrm{Var}[\delta D^t_n]$ is the spread from the same cell over several time points, and $\mathrm{Var}[\delta D_n]$ is the variance induced by cell division. Each of these variances can be determined from untreated cell clusters that are tracked through cell division. Specfically, the variance in D_n across all cells within a cluster is $\mathrm{Var}[D_{n,i}] = \mathrm{Var}[\overline{D}_n] + \mathrm{Var}[\delta D^t_n]$. Therefore, if we isolate clusters of cells that do not divide in our experiment, we can extract the values and use them to determine the remaining variance in D_n that is induced by cell division $\mathrm{Var}[\delta D_n]$.

Identifying Temporal Variance in Non-Dividing Clusters. Clusters were classified as dividing or non-dividing based on their growth ratios between two consecutive time points. Clusters that exhibited less than a 25% increase in cell count between the two time points were labeled as non-dividing. We performed a linear regression analysis to find the amount of variance in $D_{n,2}$ (the value after cell division) that is explained by $D_{n,1}$, which can be determined through the correlation coefficient. For each cluster, we calculated the mean and standard deviation of D_n values, normalizing these values by subtracting the mean D_n for all clusters at each time point $D_{n,\text{norm}} = D_n - \langle D_n \rangle$. The coefficient of determination (N^2_{non}) for this regression was calculated as 0.4081, indicating that 40.81% of the variance in the second time point's D_n values could be explained by the values from the first time point. The rest of the variance in the second time point can therefore be estimated to be due to intrinsic cluster variations.

Determining Variance from Cell Division Using Dividing Clusters. As we determined the amount of variance in a cluster that occurs due to natural temporal effects (N^2_{non}), we used this to find the temporal variance $\text{Var}[\delta D^t_n]$ with the variance of the population of dividing cells before cell division:

$$\text{Var}[\delta D^t_n] = \left(N^2_{\text{non}}\right) \text{Var}[D_{n,1,\text{div}}] \quad [37]$$

Additionally, given that the remaining variance is intrinsic cluster variations ($\text{Var}[D_{n,i}] = \text{Var}[\overline{D}_n] + \text{Var}[\delta D^t_n]$), we can determine the variance within a cluster using:

$$\text{Var}[\overline{D}_n] = \left(1 - N^2_{\text{non}}\right) \text{Var}[D_{n,1,\text{div}}]. \quad [38]$$

We can now use Eq. 36 to determine the drift that occurs due to cell division if we find $\text{Var}[\Delta D_n]$, the overall variance that occurs at after cell division due to all three variances. Given that $\Delta D_n = D_{n,2} - D_{n,1}$, we can find the population variance with:

$$\text{Var}[\Delta D_n] = \frac{1}{N_{\text{div}}} \sum_{i=1}^{N_{\text{div}}} (\Delta D_{n,i} - \overline{\Delta D}_{n,i})^2 \quad [39]$$

We then used the values from Eqs. 37, 38, and 39 in Eq. 36 to determine $\text{Var}[\delta D_n]$, given that $N_{\text{non}} = 2.27$, $N_{\text{div}} = 4.45$, $N_1 = 6.09$, and $N_2 = 11.18$. We found that the PDF describing the drift in D_n due to cell division $f(D_n - D'_n)$ can be modeled as being centered around zero with a standard deviation of 0.0685 ± 0.250. For the CDA model, we perform a convolution of the input $\text{PDF}(D_n)$ with $f(D_n - D'_n)$ to arrive at more accurate predictions of D_n after exposure to a stressor.

Closed-Form Approximation of Cell Death Probability. The non-steady-state CPMC model assumes an exponential decay in mRNA transcript numbers over time (Eq. 16). As a result, transcriptional malleability k and heterogeneity s asymptotically approach a stable plateau when $t \gg \tau$. This plateau simplifies the CPMC-derived inputs used to calculate Θ, leading to an approximate steady-state expression for the probability of cell death.

Steady-State Malleability and Heterogeneity. At steady state, the malleability ratio γ_k can be calculated via Eq. 18. If the dependence of $Se(D_n)$ on D_n is negligible - i.e., $Se_{\overline{\varepsilon}_{i,a}}(D_{n,b}) - Se_{\overline{\varepsilon}_{i,a}}(D_{n,a}) \ll Se_{\overline{\varepsilon}_{2,a}}(D_n) - Se_{\overline{\varepsilon}_{1,a}}(D_n)$ for all $D_{n,a} < D_n < D_{n,b}$ and $i = 1, 2$ - the steady-state equation simplifies to:

$$\gamma_k \approx \frac{\beta_b}{\beta_a} \approx \left(\frac{D_{n,b}}{D_{n,a}}\right)^{Se_{\overline{\varepsilon}_{2,a}}(D_{n,a}) - Se_{\overline{\varepsilon}_{1,a}}(D_{n,a})} \quad [40]$$

with $\overline{\varepsilon}_{2,a} = \beta_a \overline{\varepsilon}_{1,a}$. By combining Eqs. 5 and 6, the sensitivity equation is approximated as $Se_{\overline{\varepsilon}_{1,a}}(D_{n,a}) \approx \frac{1}{D_{n,a}} \ln N_{\text{PD}} - G(\overline{\varepsilon}_{1,a}) F(D_{n,a})$, where $G(\overline{\varepsilon}) \approx \frac{1}{2}\sigma^2_{\phi_{\text{in}}} \sqrt{\kappa/\overline{\varepsilon}}$ and $F(D_n) = D_n \ln \frac{r_{\text{in}}}{r_{\text{min}}} + \frac{3-D_n}{D_n} \frac{r_{\text{min}}}{r_{\text{in}}} L^{1/D_n} \ln L$. Substituting this into Eq. 40 and assuming κ is the same for both $\overline{\varepsilon}_{1,a}$ and $\overline{\varepsilon}_{1,b}$, we derive:

$$\gamma_k \approx \left(\frac{D_{n,b}}{D_{n,a}}\right)^{\frac{1}{2}\sigma^2_{\phi_{\text{in},a}} F(D_{n,a}) \sqrt{\frac{\kappa}{\overline{\varepsilon}_{1,a}}} \left(1 - \frac{1}{\sqrt{\beta_a}}\right)} \quad [41]$$

Given that gene upregulation is typically high ($\beta_a \gg 1$), we can further simplify Eq. 41, as $\frac{1}{\sqrt{\beta_a}}$ becomes negligible compared to 1:

$$\gamma_k \approx \left(\frac{D_{n,b}}{D_{n,a}}\right)^{\frac{1}{2}\sigma^2_{\phi_{\text{in},a}} F(D_{n,a}) \sqrt{\frac{\kappa}{\overline{\varepsilon}_{1,a}}}} \quad [42]$$

This approximation shows that under conditions of high upregulation, the malleability ratio is primarily driven by the geometric ratio of D_n values. Biologically, this implies that for highly responsive genes, the variation in expression is governed more by chromatin structure differences than by initial expression levels.

At steady state, heterogeneity is determined by solving Eq. 19, yielding $s = COV[E_2] = \sqrt{2} G(\overline{\varepsilon}_2)$:

$$s_a \approx \frac{1}{\sqrt{2}} \sigma^2_{\phi_{\text{in},a}} \sqrt{\frac{\kappa}{\beta_a \overline{\varepsilon}_{1,a}}} \quad [43]$$

$$s_b \approx \frac{1}{\sqrt{2}} \sigma^2_{\phi_{\text{in},b}} \sqrt{\frac{\kappa}{\beta_a \overline{\varepsilon}_{1,a} \gamma_{\overline{\varepsilon}_2}}} \quad [44]$$

If $Se(D_n)$ exhibits weak dependence on D_n, specifically when $Se_{\bar{\varepsilon}_a}(D_{n,b}) - Se_{\bar{\varepsilon}_a}(D_{n,a}) \ll D_{n,b} - D_{n,a}$, we approximate $\gamma_{\bar{\varepsilon}}$ as:

$$\gamma_{\bar{\varepsilon}_2} = \frac{\bar{\varepsilon}_{2,b}}{\bar{\varepsilon}_{2,a}} \approx \left(\frac{N_{\mathrm{PD}}}{A_v}\right)^{\frac{1}{D_{n,b}} - \frac{1}{D_{n,a}}} \left(\frac{D_{n,b}}{D_{n,a}}\right)^{Se_{\bar{\varepsilon}_{2,a}}(D_{n,a})} \tag{45}$$

For genes with high upregulation ($\beta_a \gg 1$), the post-stress expression rate $\bar{\varepsilon}_{2,a}$ far exceeds the critical rate κ further simplifying $Se_{\bar{\varepsilon}_{2,a}}(D_{n,a})$. This leads to:

$$\gamma_{\bar{\varepsilon}_2} \approx \left(\frac{N_{\mathrm{PD}}}{A_v}\right)^{\frac{1}{D_{n,b}} - \frac{1}{D_{n,a}}} \left(\frac{D_{n,b}}{D_{n,a}}\right)^{\frac{1}{D_{n,a}} \ln\left(\frac{N_{\mathrm{PD}}}{A_v}\right)} \tag{46}$$

$$\gamma_h \approx \left(\frac{N_{\mathrm{PD}}}{A_v}\right)^{\frac{1}{2}\left(\frac{1}{D_{n,a}} - \frac{1}{D_{n,b}}\right)} \left(\frac{D_{n,a}}{D_{n,b}}\right)^{\frac{1}{2D_{n,a}} \ln\left(\frac{N_{\mathrm{PD}}}{A_v}\right)} \left(\frac{r_{\min}}{r_{\mathrm{in}}}\right)^{D_{n,a} - D_{n,b}} \tag{47}$$

$$h_b \approx \frac{3}{s_b\sqrt{\pi}} \approx 3\sqrt{\frac{2}{\pi}} \frac{1}{\sigma^2_{\phi_{\mathrm{in},b}}} \sqrt{\frac{\bar{\varepsilon}_{1,a}\beta_a}{\kappa}} \left(\frac{N_{\mathrm{PD}}}{A_v}\right)^{\frac{1}{2}\left(\frac{1}{D_{n,b}} - \frac{1}{D_{n,a}}\right)} \left(\frac{D_{n,b}}{D_{n,a}}\right)^{\frac{1}{2D_{n,a}} \ln\left(\frac{N_{\mathrm{PD}}}{A_v}\right)} \tag{48}$$

Linear Approximations Using D_n. We can simplify the complex exponential terms by expressing them as linear functions of $\Delta D_n = D_{n,b} - D_{n,a}$. When $\Delta D_n \ll D_{n,a}$, the following approximations hold:

$$\left(\frac{N_{\mathrm{PD}}}{A_v}\right)^{\frac{1}{2}\left(\frac{1}{D_{n,b}} - \frac{1}{D_{n,a}}\right)} \approx 1 + \frac{1}{2}\left(\frac{1}{D_{n,b}} - \frac{1}{D_{n,a}}\right) \ln\left(\frac{N_{\mathrm{PD}}}{A_v}\right) \tag{49}$$

$$\approx 1 - \frac{\Delta D_n}{2D_{n,a}^2} \ln\left(\frac{N_{\mathrm{PD}}}{A_v}\right) \tag{50}$$

$$\left(\frac{D_{n,b}}{D_{n,a}}\right)^{\frac{1}{2D_{n,a}} \ln N_{\mathrm{PD}}} \approx 1 + \frac{\Delta D_n}{2D_{n,a}^2} \ln\left(\frac{N_{\mathrm{PD}}}{A_v}\right) \tag{51}$$

where $\frac{1}{(2D_{n,a})} \ln\left(\frac{N_{\mathrm{PD}}}{A_v}\right) \sim 2$. Using these approximations, we simplify Eqs. 47 and 48:

$$\gamma_h \approx \left(\frac{r_{\min}}{r_{\mathrm{in}}}\right)^{D_{n,a} - D_{n,b}} \left(1 + \frac{\Delta D_n}{D_{n,a}^2} \ln\left(\frac{N_{\mathrm{PD}}}{A_v}\right)\right) \tag{52}$$

$$h_b \approx 3\sqrt{\frac{2}{\pi}} \frac{1}{\sigma^2_{\phi_{\mathrm{in},b}}} \sqrt{\frac{\bar{\varepsilon}_{1,a}\beta_a}{\kappa}} \tag{53}$$

Substituting 53 into 42 gives:

$$\gamma_k^{h_b} \approx \left(\frac{D_{n,b}}{D_{n,a}}\right)^{\frac{3}{2\pi} F(D_{n,a}) \left(\frac{r_{\min}}{r_{\mathrm{in}}}\right)^{D_{n,b} - D_{n,a}} \left(\sqrt{\beta_a} - 1\right)} \tag{54}$$

Here, $\frac{3}{2\pi} F(D_{n,a}) \approx 12.6$. We introduce the constants $Q = \frac{3}{2\pi} F(3) \approx \frac{9}{\sqrt{2\pi}} \log\left(\frac{r_{\mathrm{in}}}{r_{\min}}\right) \approx 12.6$ and $N_{\mathrm{PD}} = A_v \left(\frac{r_{\mathrm{in}}}{r_{\min}}\right)^{D_n}$, where $N_{\mathrm{PD}} \sim 15$ kbp, representing the number of base pairs in a gene's interaction volume for $D_n = 2.5$ and $r_{\min} = 1$ nm. From this, we derive the final approximations:

$$\tilde{\gamma}_h = N_{\mathrm{PD}}^{\Delta D_n / D_{n,a}} \tag{55}$$

$$\tilde{\gamma}_k^{h_b} = \left(1 + \frac{\Delta D_n}{D_{n,a}}\right)^{Q(\sqrt{\beta_a} - 1)/N_{\mathrm{PD}}^{\Delta D_n / D_{n,a}}} \tag{56}$$

Substituting these into Eq. 15, we obtain a closed-form expression for Θ:

$$\tilde{\Theta}_b(\Theta_a) = \left(1 + \left(1 + \frac{\Delta D_n}{D_{n,a}}\right)^{Q(\sqrt{\beta_a} - 1)/N_{\mathrm{PD}}^{\Delta D_n / D_{n,a}}} \left(\frac{1}{\Theta_a} - 1\right)^{\frac{1}{N_{\mathrm{PD}}^{\Delta D_n / D_{n,a}}}}\right)^{-1} \tag{57}$$

This equation shows that $\Theta_b(\Theta_a)$ primarily depends on $D_{n,a}$, ΔD_n, and β_a, linking chromatin structure changes, gene upregulation, and cell survival probability.

Simplified Linear Expressions for Malleability and Heterogeneity. To further simplify the expressions for malleability and heterogeneity, we start with the interaction volume defined as $N_{\mathrm{PD,a}} = \left(\frac{r_{\mathrm{in}}}{r_{\mathrm{min}}}\right)^{D_{n,a}}$. Using this definition, we derive $\tilde{\gamma}_h \approx \frac{N_{\mathrm{PD,a}}}{N_{\mathrm{PD,b}}}$ and $\tilde{\gamma}_k^{h_b} \approx (D_{n,b}/D_{n,a})^{Q(\sqrt{\beta_a}-1)\gamma_h}$. In a first-order approximation, we can express these terms in a more linearized form. When ΔD_n is small, $\tilde{\gamma}_h \approx 1 - \Delta D_n \ln\left(\frac{r_{\mathrm{in}}}{r_{\mathrm{min}}}\right) = 1 - \frac{\Delta D_n}{D_{n,a}} \ln N_{\mathrm{PD}}$ and $\tilde{\gamma}_k^{h_b} \approx 1 + \left(\frac{\Delta D_n}{D_{n,a}}\right) Q(\sqrt{\beta_a} - 1)$, where $N_{\mathrm{PD}} \equiv N_{\mathrm{PD,a}}$. By assuming realistic values for r_{min}, we can further simplify the logarithmic terms. This leads to the approximations $\tilde{\gamma}_h \approx 1 - 3\Delta D_n$ and $\tilde{\gamma}_k^{h_b} \approx 1 + 3\Delta D_n(\beta_a - 1)$. These equations indicate that increasing the DNA base pairs within the transcriptional interaction volume enhances both the malleability and heterogeneity of gene expression. Specifically, the results show that transcriptional sensitivity to changes in chromatin packing is influenced by both gene upregulation (β_a) and structural alterations (ΔD_n). The findings suggest that for highly responsive genes, variations in expression are driven more by differences in chromatin structure than by initial expression levels.

Adaptive Inhibition Model for Tumor Growth. While the equations derived in the previous sections for predicting the number of cells after a treatment are feasible to study experimentally using *in vitro* cultures, it is difficult to produce the same measure for *in vivo* studies. Therefore, we derived an equation to test the CDA model predictions directly in PDX experiments using the volume of a tumor as a proxy for cell number. We define the relative tumor volume (RTV) as $RTV(t) = V_{\mathrm{treatment}}(t)/V_{\mathrm{control}}(t)$ which is roughly equivalent to the probability of cell survival P_s. The CDA model predictions demonstrate that the efficacy of chemotherapy should decrease over time, as indicated by the decrease in EIR (Fig. S13). This similarly results in a rapid decrease of the RTV until it plateaus due to reduced efficacy. This indicates that the speed of acclimation of cancer cells to chemotherapy is in part due to the PDF(D_n). Immediately after treatment (small t), the EIR should decrease linearly due to the shift of the population average D_n. However, at longer time points (larger t), only the tail of the PDF(D_n) is affected by the chemotherapy.

Building on this observation, we posit that tumor growth is controlled by the cell death induced by chemotherapy (u) and the rate of adaptation to the treatment (a). Additionally, within a tumor, there exists an population of low D_n cells that cannot adapt to therapy and one with high D_n cells that can. We define the relative growth rate of tumors, $V(t)$, through $\ln(V(t)/V(0)) = (p - c)t$, where p represents the tumor growth rate in the absence of treatment and c denotes the growth inhibition rate from chemotherapy. To account for adaptation, we introduce a cumulative adaptation term, $P_a(t)$, resulting in the revised equation $\ln(V(t)/V(0)) = (p - c)t + c\int_0^t P_a(t)dt$. The integral term $c\int_0^t P_a(t)dt$ captures the accumulation of the adaptable cell population over time; however, this term does not imply active proliferation among adaptable cells. Instead, it reflects the ability of this population to mitigate the overall inhibitory effects of chemotherapy, thus enhancing tumor survival through time-dependent adaptation. We anticipate that the adaptation term $P_a(t)$ will increase until it plateaus at a maximum value, $P_a^{\max}$, influenced by treatment strength. By assuming $P_a(t) = P_a^{\max}(1 - \exp(-at))$ and defining the unadaptable inhibition rate as $u = c(1 - P_a^{\max})$, we derive the final adaptation model:

$$\ln \frac{V(t)}{V(0)} = (p - u)t - \frac{c - u}{a}(1 - \exp(-at)) \qquad [58]$$

where $V(t)$ is the tumor volume at time t, p is the proliferation rate, u is the unadaptable inhibition rate, c is the initial inhibition rate, and a is the adaptation rate. The parameters p and u are specific to the cell line and chemotherapy, while c and a depend on the treatment modality and the evolution of PDF(D_n) over time. The parameters u, c, and a were estimated from the data, while a fixed proliferation rate of $p = 1 \times 10^{-10}$ was used in all analyses. This dependence on PDF(D_n) encapsulates both the adaptive capacity of cells undergoing treatment and the baseline inhibition imposed on unadaptable cells.

Establishment of TPR and Inhibition Indices. As it is difficult to simultaneously evaluate many potential drug candidates to determine which one has the most significant impact on chromatin and, subsequently, cell death, we sought to derive a quantifiable value to characterize the magnitude of change. To find this, we use Eq. 58, which tells us the change in cancer cell number as a function of multiple inhibition and adaptation rates. Simply plotting the change in D_n against the the cell number $N(t)$ would depict a general trend but not a clear relationship. Therefore, we wanted to determine a value that is a function of N and D_n so that we can better visualize the relationship between the two. However, in order to do that, we need to know all of the rates that are input into Eq. 58. For simplification, we derived an equation to eliminate several terms such that we could use values from the CDA model to predict the trend. Since rates p and u are specific to a particular cell line and chemotherapy, we can compare the effects of two potential TPRs to eliminate the need to know these rates. The rates c and a depend on the PDF(D_n) and the TPR. To eliminate p, we consider three groups of cells (in the same cell line): one treated with a chemotherapy (high D_n), and the others treated with two different candidate TPRs (low D_n).

Here, we consider two groups of cells: one treated with TPR A and another treated with TPR B, both on the same cell line. For $a_A t \gg 1$ and $a_B t \gg 1$, Eq. 58 becomes:

$$\ln \frac{N_A(t)/N_A(0)}{N_B(t)/N_B(0)} \approx \frac{c_B - u}{a_B} - \frac{c_A - u}{a_A} \qquad [59]$$

The rate c can be approximated as $c \approx \Theta/\tau$. We can use the derived approximate equations for $\tilde{\gamma}_h$ and $\tilde{\gamma}_k^{h_b}$ to simplify Eq. 15. First, from Eq. 55, $\tilde{\gamma}_h$ can be approximated using $\tilde{\gamma}_h \approx 1 - (\Delta D_n/D_n)\ln(N_{\mathrm{PD}})$. Then, from Eq. 56, $\tilde{\gamma}_k^{h_b}$ can be simplified to

$\tilde{\gamma}_k^{h_b} \approx 1 + (\Delta D_n/D_n)Q(\sqrt{\beta_a} - 1)$. By plugging these approximations into Eq. 15 and defining $\gamma = \ln(N_{\text{PD}}) + Q(\sqrt{\beta_a} - 1)$ we find

$$\Theta_B = \Theta_A \left(1 - \gamma \frac{D_{n,B} - D_{n,A}}{D_{n,A}}\right) \quad [60]$$

Here, $\gamma \approx 10$ for physiologically relevant N_{PD} and β_a. Therefore, a $\Delta D_n \approx 0.1$ can lead to large changes in Θ. From $c \approx \Theta/\tau$, we find that $c_B \approx c_A \left(1 - \gamma \frac{D_{n,B} - D_{n,A}}{D_{n,A}}\right)$. Therefore, $c_B - c_A \approx -c_A \gamma \frac{D_{n,B} - D_{n,A}}{D_{n,A}}$ and $c_B - u \approx -u\gamma \frac{D_{n,B} - D_{n,u}}{D_{n,u}}$, where $D_{n,u}$ is chosen to correspond to u. Similarly, the rate a can be expanded as $a_A \approx a_B \left(1 - \gamma \frac{D_{n,B} - D_{n,A}}{D_{n,B}}\right)$. Substituting into Eq. 59 gives $\ln(N_A/N_B) \approx (\gamma/a_B)u(D_{n,A} - D_{n,B})$. Let N_C represent the number of cells in a control condition without TPR. Then, taking logs relative to the control:

$$\frac{\ln(N_A/N_C)}{\ln(N_B/N_C)} \approx \frac{D_{n,A} - D_{n,C}}{D_{n,B} - D_{n,C}} \quad [61]$$

This expression allows estimation of relative chromatin states across treatments. In cases where D_n is a distribution (i.e., $\text{PDF}(D_n) \neq \delta(D_n - D_{n,A})$), Eq. 61 can still apply with effective values such as $D_{n,A} \pm \sigma$, where σ is the standard deviation of the D_n distribution.

Substituting Eq. 60 into the expression for relative tumor volume (RTV) difference between two populations $\ln \text{RTV}_A - \ln \text{RTV}_B = \ln\left(\frac{N_A}{N_B}\right) \approx -\frac{c_A \gamma}{a_A}(1 - e^{-a_A t})$. For short timescales $a_A t \ll 1$, this reduces to $\ln\left(\frac{N_A}{N_B}\right) \approx -c_A \frac{\Delta D_n}{D_n} t$. Therefore, the relative reduction in ln(RTV) is proportional to the fractional change in D_n scaled by γ, $\%\Delta \ln \text{RTV} \approx \gamma \%\Delta D_n$. This analysis highlights that even moderate reductions in D_n (e.g., 10%) can result in large decreases in Θ and RTV due to the multiplicative amplification by γ.

Supplementary Materials and Methods

Cell Culture and Treatments. We obtained leiomyosarcoma (MES-SA, #CRL-1976; MES-SA.MX2, #CRL-2274), breast (MDA-MB-231, #HTB-26), colon (HCT-116, #CCL-247; HT-29, #HTB-38), and mouse embryonic fibroblast (MEF) cell lines from ATCC. Cells were maintained in their respective media as per ATCC protocols, supplemented with 10% FBS (#10-082-147, ThermoFisher Scientific). Ovarian cell lines (A2780, A2780.M248, A2780.M273, A2780.M175, and Ovcar8) were provided by Dr. Chia-Peng Huang Yang, originally sourced from Dr. Elizabeth de Vries's lab at Albert Einstein College of Medicine (16). Human mesenchymal stem cells (hMSCs, #PCS-500-012, ATCC) were cultured in Dulbecco's Modified Eagle Medium (DMEM) with 4.5 g/L glucose (#11965092, ThermoFisher Scientific), supplemented with 10% FBS and 5 mL of 10× penicillin-streptomycin (#151400-122, ThermoFisher Scientific). For differentiation studies, hMSCs were seeded at 1.5×10^4 cells/mL in 24-well glass-bottom plates (#P24-1.5H-N, Cellvis). After 2 days, cells were transitioned to hMSC Osteogenic Differentiation Medium containing β-glycerophosphate, ascorbate, and dexamethasone (#PT-3002, Lonza). Media were changed every other day, and cells were imaged on day 4 post-induction. All cell lines were tested for mycoplasma using Hoechst 33342 (#H3570, ThermoFisher Scientific) and all experiments utilized cells from passages 5 to 20.

For imaging experiments, cells were seeded on 35 mm glass-bottom Petri dishes (#D35-14-1.5-N, Cellvis), allowing at least 24 hours after trypsinization for re-adherence prior to treatment. All pharmaceutical agents were purchased from Sigma Aldrich, unless otherwise noted. We treated cells with chemotherapy agents for at least 48 hours prior to imaging: paclitaxel (48 hours, 5 nM), oxaliplatin (48 hours, 15 µM), 5-fluorouracil (72 hours, 500 nM), docetaxel (48 hours, 5 nM), gemcitabine (48 hours, 50 nM). For TPR treatments, cells were treated for 30 minutes prior to imaging with the following compounds at specified concentrations: celecoxib (75 µM, #SML3031), valproic acid (100 µM), aspirin (1 mM), digoxin (100 nM, #D6003), UNC0638 (1 µM), UNC1999 (1 µM), EGCG (25 nM), ginsenoside RB2 (1 µM), curcumin (25 µM), 4-phenylbutyrate (100 mM), simvastatin (1 µM), mevastatin (1 µM), resveratrol (1 µM), valinomycin (1 µM, #V3639).

Partial Wave Spectroscopic (PWS) Microscopy. We performed Partial Wave Spectroscopic (PWS) microscopy using a commercial inverted microscope (Leica DMIRB) equipped with a Hamamatsu Image-EM CCD camera (C9100-13) and a liquid crystal tunable filter (LCTF; CRi). The PWS microscope setup used in this study is described in detail by Almassalha et al. (5). All cells were maintained and imaged under physiological conditions (5% CO_2 and 37°C) throughout imaging. Monochromatic spectrally resolved images were acquired from 500 to 700 nm at 1 nm intervals, with illumination provided by an Xcite-120 LED Lamp (Excelitas). The images were normalized by the incident light reflectance from the glass-media interface, using an independent reference image from a cell-free field of view to scale the reflectance. A low-pass Butterworth filter with an order of 2 and a cut off frequency of 0.2 nm^{-1} was applied to reduce spectral noise prior to calculating the variance of interference spectra (Σ^2) at each pixel (7). The wavelengths of the captured data were cropped to retain those between 510-690 nm, as the edge wavelengths are often noisy.

To determine D_n for individual cells, we averaged Σ^2 over all pixels across cell nuclei, then converted $\langle \Sigma^2 \rangle$ to D_n using custom MATLAB scripts (4). In brief, the average nuclear spectral variance $\langle \Sigma^2 \rangle$ is multiplied by a correction factor of 2.43 to account for differences in internal reflection between microscopes. The resulting $\langle \Sigma^2 \rangle$ is then converted to D_n using our previously published computational model (4) using input values. Typically, 100 to 200 cells were analyzed per condition to ensure robust statistical comparisons. Each treated cell population measured by PWS microscopy was compared to an untreated control population of the same cell type, plated on the same day with identical seeding density.

Additionally, pseudo-colored live cell PWS images were generated using Python, first by converting the Σ^2 for each image pixel-wise to D_{pixel}, then mapping D_{pixel} values to a red colormap. D_{pixel} values ranging from 2 to 3 were visually represented, with higher values indicated by brighter red, providing a spatial map of chromatin packing domain variations across the nucleus.

Cell Viability Assay. We performed cell viability assays using fluorescence measurements with a BioTek Synergy Neo2 Reader at the Northwestern University HTAL Core. HCT116 cells were plated on 96-well flat, clear-bottom plates at a seeding density of 1,500 cells per well. 48 hours after plating, we treated the cells with oxaliplatin (15 µM) and evaluated them at 0, 2, 6, 12, 24, and 48-hour time points using the ApoTox-Glo triplex assay (#G6320, Promega). We added 20 µL of the assay viability reagent to each well, incubated at 37°C for 30 minutes, and measured fluorescence intensity (EX 400/20, EM 505/20, gain 87).

Single Molecule Localization Microscopy (SMLM) Staining, Imaging, and Analysis. Components of the EdU staining kit (Thermo Fisher Scientific) were stored according to the manufacturer's instructions following reconstitution. Cells were seeded at 12,500–25,000 per well on No. 1 borosilicate-bottom, eight-well Lab-Tek chambered coverglass and allowed to adhere for 48 hours. EdU was added and incubated overnight to label cells throughout one complete cell cycle. Cells were then treated with oxalipplatin for 48 hours. Samples were then fixed for 10 minutes at room temperature in 4% paraformaldehyde in PBS, followed by three 5-minute washes in PBS. Incorporation of EdU was visualized via click-chemistry–based secondary staining with AF488 fluorophore, performed exactly as outlined by the kit manufacturer.

The STORM optical setup was built around a Nikon Eclipse Ti-U inverted microscope equipped with Nikon's Perfect Focus System. For STORM acquisition, a 488 nm laser (Coherent Obis laser box) was focused through a 100x, 1.49 NA TIRF objective (Nikon SR APO), delivering 3–10 kW/cm^2 at the sample. Emitted photons were collected by the same 100× lens and recorded on an Andor iXon Ultra 888 EMCCD. Typically, 10,000 frames were acquired per field of view with 30 ms exposure times.

The raw image sequence was first pre-processed in ImageJ by subtracting its minimum-intensity projection and then applying a 5-pixel rolling-ball background subtraction to every frame. The preprocessed stack was imported into the ThunderSTORM plugin, where the camera was configured to match the acquisition settings: the pixel size of 130 nm (sensor pixel dimension divided by objective magnification), 18.6 photoelectrons per A/D count, a zero-count base level, and an EM gain of 240. For each cell, the nuclear ROI was drawn by Nuclei Outline in GDSC plugin in ImageJ. Within each region, localizations were clustered using DBSCAN ($\epsilon = 50$ nm, minPts = 3). Cluster size was then measured as the area of the convex hull around each cluster of points, normalized by a factor of $\pi \cdot (80\text{ nm})^2$, and filtered to include only values between 0.1 and 100.

Calculation of Θ from Predicted D_n Distributions. Given that $1 - \Theta = e^{-\text{IR}(\Delta t)\cdot\Delta t}$, we can use data from cell cluster tracking experiments to determine the probability of cell death as long as we have the initial distribution of D_n prior to the addition of chemotherapy. To find experimental Θ values from PWS microscopy data on PDFs of D_n at various timepoints, we employed an iterative solution to solve Eq. 30, as the integral-differential equation is difficult to solve analytically:

$$\begin{cases} N(D_n, \Delta t) & \approx 2^{\Delta t/\tau_2} \int N(D'_n, 0) \cdot f(D_n - D'_n)\ dD'_n\ e^{-\text{IR}(D_n,\Delta t)\cdot\Delta t} \dots \\ N(D_n, n_t\Delta t) & \approx 2^{n_t\Delta t/\tau_2} \int N(D'_n, (n_t-1)\Delta t) \cdot f(D_n - D'_n)\ dD'_n\ e^{-\text{IR}(n_t\Delta t)\cdot n_t\Delta t} \end{cases} \quad [62]$$

where Δt is any small interval (i.e., one day) and n_t is the number of time intervals. We analyzed confluence data for HCT116 cells to calculate the mean and standard deviation of confluence across experimental groups. The overall inhibition rate for all D_n at a single time point, $\text{IR}(n_t\Delta t)$, is calculated using the ratio of confluence in treated groups to control groups at time t. The drift due to cell division $f(D_n - D'_n)$ was determined to be normally distributed around a mean value of 0 with a standard deviation of 0.057 using untreated control cluster tracking experiments, as described in the section "Estimation of Cell Division Induced Drift in Population D_n". When $\Delta t \to 0$ and $n_t \to \inf$, the iterative solution should converge to the exact solution.

The number of cells as a function of D_n and time, $N(D_n, \Delta t)$, is essentially the $\text{PDF}(D_n)$ at a specific time point. Therefore, to solve Eq. 62, we used the PDF at $t = 0$ from the experiment to produce an estimate of what the PDF would be at 48 hours using an initial guess of the $\Theta(D_n)$. PDFs of D_n values were generated from experimental data using histogram methods with a bin width of 0.1. For each doubling time (τ_2), we convolved the current PDF with the PDF incorporating drift in D_n due to one division, $f(D_n - D'_n)$, as $\text{PDF}(D_{n,2}) = \text{PDF}(D_{n,1}) * (\sqrt{n_{\text{div}}} \cdot \sigma_{\delta D_n})$, where $\text{PDF}(D_{n,1})$ is the PDF at the first time point, n_{div} is the number of divisions, and $\sigma_{\delta D_n} = 0.057$ is the standard deviation of the division-induced drift. The number of divisions over 48 hours was calculated as $n_{\text{div}} = 48/\tau_2 = 2.71$ cell divisions. We estimated τ_2 using the total cell number across clusters at 0 and 24 hours with $\tau_2 = 24/\log_2[N(24h)/N(0h)] = 17.71$ hours. The PDF that resulted from the convolution was then multiplied by 2 to account for cell population doubling and normalized. Subsequently, we multiplied the PDF by the probability of cell survival (1-Θ) for each D_n value. To account for incomplete cell cycles, we calculated the residual doubling time (remainder of $48/\tau_2$) using an appropriate correction factor and expected cell population increase for the remaining time.

To find the experimental Θ in the cluster tracking PWS data of HCT116 cells treated with oxaliplatin, we used a two-step sum of squared errors (SSE) minimization process. We first calculated an initial prediction for Θ for each D_n value at the second time point as $\Theta(D_n) = 1 - [\text{PDF}(D_{n,1})/\text{PDF}(D_{n,2}) \cdot \text{IR}(t)]^{\tau_2/t}$. The vector of Θ values was then used as the argument of the objective function for SSE minimization. We minimized the PDF shape-related error between the actual and predicted D_n distributions at 48 hours, defined as $\text{SSE}_{\text{shape}} = \sum_i (\text{PDF}_{\text{actual}}(D_{n,i}) - \text{PDF}_{\text{predicted}}(D_{n,i}))^2$. We then refined the fit by minimizing a combined error that includes both shape and area under the curve, given by $\text{SSE}_{\text{total}} = \text{SSE}_{\text{shape}} \cdot \left(\sum \text{PDF}_{\text{predicted}} - 1\right)^2$, where $\sum \text{PDF}_{\text{predicted}}$ is the sum of all values in the predicted PDF multiplied by the bin width used in the PDF calculation.

Cell Confluence Measurements. To calculate drug inhibition rates, we measured cell confluence by assessing cell density on dishes using transmission microscopy, quantifying the relative inhibition of cells in response to chemotherapeutic agents alone or in combination with potential TPR agents. We measured cell plate density for an area spanning 600,000 to 2,500,000 μm^2 using either a 40× or 20× air objective prior to PWS microscopy measurements. These measurements were obtained for three independent dishes for each condition group. We quantified cell density from transmission microscopy images using ImageJ (version 1.53c), which employs automatic thresholding and particle analysis to determine the area occupied by cells. We calculated the inhibition rate (IR) for each treatment condition using the following equation:

$$\mathrm{IR} = \frac{1}{t_{n+1} - t_n} \cdot \ln\left(\frac{C(t_{n+1})}{C(t_n)}\right) \tag{63}$$

where t_n represents the time in days. For measurements looking at the amount of cell death, we calculated normalized inhibition (I_{norm}) as the ratio of confluence in treated groups to that in control groups:

$$I_{\mathrm{norm}} = \frac{C_{\mathrm{treatment}}}{C_{\mathrm{control}}} \tag{64}$$

Inhibition and TPR Index Calculation. The relative inhibition between the control and drug-treated conditions was determined using the following formula:

$$\text{Inhibition Index} = \ln\left(\frac{1 - I_{\mathrm{Chemo}}}{1 - I_{\mathrm{TPR1}}}\right) / \ln\left(\frac{1 - I_{\mathrm{Chemo}}}{1 - I_{\mathrm{TPR2}}}\right) \tag{65}$$

where I represents the mean inhibition due to the indicated treatment. This measure quantifies the effect of the drug treatment relative to the control, taking into account the inhibition levels in both conditions. The TPR index, reflecting relative chromatin packing domain modulation between two drug treatments, was calculated across cell lines by comparing their normalized responses. To compare two drugs (denoted as TPR1 and TPR2), the following formula was used to compute the TPR Index:

$$\text{TPR Index} = \frac{D_{n,\mathrm{TPR1}} + \sigma_{D_{n,\mathrm{TPR1}}}}{D_{n,\mathrm{Ctrl}} + \sigma_{D_{n,\mathrm{Ctrl}}}} / \frac{D_{n,\mathrm{TPR2}} + \sigma_{D_{n,\mathrm{TPR2}}}}{D_{n,\mathrm{Ctrl}} + \sigma_{D_{n,\mathrm{Ctrl}}}} \tag{66}$$

where D_n is the mean normalized value for each drug and σ_{D_n} is the standard deviation of the PDF(D_n). This index accounts for differences in chromatin packing domain modulation and treatment efficacy between two drugs, adjusted for population heterogeneity in response.

Calculation of Θ from Population-Level Cell Confluence Data. To estimate the cell death probability (Θ) for chemo-treated cells to look at the influence of TPRs, we developed an approach based on cell confluence data. This method compares expected growth rates (without drug inhibition) to actual growth rates (with drug inhibition) following the logic of Eq. 28, ultimately providing the inhibition rate (IR) for each treatment. The confluence at some time interval Δt is:

$$C_{n_t+1} \equiv C(\Delta t + n_t \Delta t) = C(n_t \Delta t) \exp\left[g(n_t \Delta t) - \mathrm{IR}(n_t \Delta t)\right] \tag{67}$$

For our calculations, we use $\Delta t = 1$ day.

Growth rate was calculated by solving Eq. 67 for g in an untreated control population such that $g(n_t) = \ln\left[C(n_t+1)/C(n_t)\right]$ for $\Delta t = 1$ day. We removed negative growth rates, implying cell death or loss, from subsequent analyses as such observations were not expected in the control conditions. We then used a linear regression to model the relationship between confluence and growth rate with $g = m \cdot C + b$, where m is the slope and b is the intercept. The regression yielded an $R^2 = 0.78$, indicating that it accounted for a significant portion of the variability in the data.

The IR during the early treatment phase (days 1 to 3) was determined for all conditions using two complementary methods. The primary method utilized confluence ratios (Eq. 63), quantified changes in confluence over time for all treatments. For comparative analysis, particularly when evaluating drug treatments against the control, the difference between expected and actual growth rates was used $\mathrm{IR}_i = g_{\mathrm{expected},i} - g_{\mathrm{actual},i}$, where $g_{\mathrm{expected},i}$ represents the expected growth rate (from the control model) and $g_{\mathrm{actual},i}$ is the growth rate under drug treatment i. This was especially useful in dose-response analyses and for calculating the average IR for treatments like celecoxib.

We additionally calculated the overall growth rate using $g_2 = \ln(C_2/C_0)/2$ for $\Delta t = \tau_2 = 2$ days, where C_2 is the confluence on day 3 and C_0 is the confluence on day 1. An initial cell confluence C_0 of 33% was chosen as the baseline confluence for all conditions, as this was the average value determined by the cell confluence algorithm for day 0. The cell doubling time τ_2 was set to 2 days. These constants were applied across the control, paclitaxel, and celecoxib treatment groups.

The mean growth suppression by celecoxib was calculated using data from treatment-specific time points. For celecoxib-treated samples, we calculated normalized growth inhibition using Eq. 64. Averaged values across treatment dates provided an estimate of celecoxib's efficacy in inhibiting cell proliferation. Growth rates for celecoxib-treated cells were adjusted based on the mean growth suppression. The average IR for celecoxib was used to modify the growth rates, and a two-parameter exponential model was fitted to the data $\mathrm{IR}_{\mathrm{Celecoxib}} = a \times \mathrm{e}^{b \times \mathrm{Confluence}}$, where a and b are fitting parameters, capturing the non-linear relationship between celecoxib concentration and its inhibitory effect.

For cells treated with paclitaxel alone, Θ was calculated by fitting an exponential growth model to the observed data $\Theta = 1 - \mathrm{e}^{-\mathrm{IR} \times t}$, where IR is the inhibition rate and t is the treatment duration in days. Θ values on day 1 were calculated directly

from the IR data. For combination treatments (TPR + chemotherapy), the effective cell death probability was calculated by subtracting the IR values of the combination-treated group from the celecoxib-treated group $\Theta_{\text{Combination-Celecoxib}} = 1 - e^{-(\text{IR}_{\text{Combination}} - \text{IR}_{\text{Celecoxib}})t}$, where $\text{IR}_{\text{Combination}}$ is the inhibition rate of the combination treatment and $\text{IR}_{\text{Celecoxib}}$ is the inhibition rate of celecoxib alone. On day 2, for samples treated with both celecoxib and paclitaxel, inhibition was assessed by matching confluence values from the combination treatment group to the closest paclitaxel-only group. Adjustments were made for initial confluence and the duration cells had been plated prior to treatment.

Final Θ values were calculated using $\Theta = 1 - (1 - \Theta)^{(\ln(2)/(b + m \times \text{Confluence}))}$, where b and m are the intercept and slope from the control growth rate regression. This equation adjusts the cell death probability based on the growth rate's dependence on confluence. To ensure data quality, we applied a filter to exclude samples with standard deviation values greater than 0.2, ensuring only reliable data were used in the final analysis.

Macromolecular Crowding Model. We simulated mRNA synthesis dynamics under macromolecular crowding using the kinetic models described by Matsuda et al. (10) and Shim et al. (11). This approach quantifies how variations in transcription factor and RNA polymerase concentrations affect mRNA production in crowded environments. The simulations, conducted in Python, compute mRNA expression profiles across different crowding conditions, denoted as $\phi_{\text{in,model}}$, which represent the degree of macromolecular crowding. Matrix operations were handled using NumPy, while symbolic solutions were obtained with SymPy. Parameter values were initialized according to Matsuda et al. (10), unless otherwise specified. The model employed a fixed mRNA synthesis rate, k_m, and dissociation constants for transcription factors (K_D^{TF}) and RNA polymerase ($K_D^{\text{Pol II}}$). The optimal k_m was determined iteratively by aligning the model's maximum mRNA production at $\phi_{\text{in,model}}$ with the experimentally observed $\phi_{\text{in,experiment}}$ (within a margin of error of 0.005).

We simulated mRNA synthesis across a range of transcription factor and RNA polymerase concentrations (denoted TR), spanning from 1×10^{-7} to 1×10^{-2} mM. mRNA production was modeled using differential equations that describe the binding and dissociation interactions between transcription factors, RNA polymerase, and DNA, while accounting for crowding effects. The dissociation rates for RNA polymerase and transcription factors were calculated based on total DNA concentration and their respective dissociation constants, adjusted according to the crowding factor $\phi_{\text{in,model}}$, which ranged from 0 to 0.505, reflecting physiological crowding conditions. Crowding effects on transcription kinetics were incorporated by modifying rate constants based on empirical coefficients derived from previous Brownian Dynamics simulations (10). The system of nonlinear equations governing transcription was solved symbolically to obtain mRNA synthesis rates across different crowding levels.

For each simulation condition, we computed the steady-state mRNA concentration, defined as the maximum of the mRNA expression curve, and plotted the relationship between $\phi_{\text{in,model}}$, transcriptional reactant (TR) concentrations, and mRNA output. To evaluate the dynamics of mRNA synthesis, we calculated the peak mRNA concentration and its corresponding $\phi_{\text{in,model}}$, as well as the second derivatives of the mRNA expression curves. The outputs that were then used to approximate $\overline{\varepsilon}$ included the maximum steady-state mRNA concentrations, the $\phi_{\text{in,model}}$ values at which they occurred, and the curvature of the mRNA response, assessed via second derivative analysis.

The MC model outputs mRNA concentration ([mRNA]), which can be converted to the number of mRNA transcripts (N) using the cell volume $V_{\text{cell}} = 500\ \mu\text{m}^3$ (typical for HeLa cells): $N = [\text{mRNA}] \times N_A \times V_{cell}$, where N_A is Avogadro's number. The steady-state expression rate ε is then calculated as $\varepsilon = N \times \nu$ where $\nu = 3 \times 10^{-4}\ \text{s}^{-1}$ is the degradation rate (10).

Chromatin-Dependent Adaptability (CDA) Model Implementation and Optimization. We implemented the CDA model using Python with NumPy and SciPy libraries. First, to investigate the impact of the key parameters on model predictions, we performed parameter scans. The key parameters scanned were $D_{n,0}$ (initial chromatin packing domain behavior), $\ln(E/\overline{E})$ (initial gene expression), $\beta_a = E_2/E_1$ (transcriptional amplification factor), x_{crit} (critical upregulation threshold), and t_{crit} (critical decision time). We employed a grid search approach, systematically varying these parameters within physiologically relevant ranges.

For comparisons between model predictions and experimental data, we employed the `scipy.optimize.minimize` function to minimize the SSE. We used the L-BFGS-B algorithm for minimization, allowing for bounded optimization of parameters within physiologically relevant ranges. For the optimization for Θ vs. D_n, the objective function for minimization was $f(\vec{p}) = \sum_{i=1}^{n} [\Theta_{\text{model}}(D_{n,i}, \vec{p}) - \Theta_{\text{exp}}(D_{n,i})]^2$, where $\vec{p}$ is the vector of model parameters, $D_{n,i}$ are the experimental D_n values, and $\Theta_{\text{exp},i}$ are the experimental Θ values. The optimization was constrained with bounds of $[0, 1]$ for all Θ values to ensure biological plausibility. To determine the optimal values for β_a, x_{crit}, and $\ln(E/\overline{E})$, we performed a grid search over physiologically relevant ranges: β_a from 1 to 30, x_{crit} from 1 to 30, and $\ln(E/\overline{E})$ from -6 to 4. For each parameter combination, we calculated the SSE between the model-predicted and experimental Θ vs. D_n curves, selecting the parameter set yielding the lowest SSE as optimal. Optimization yielded parameters consistent with key survival genes exhibiting higher initial expression ($\ln(E/\overline{E}) \approx 2$) and significant upregulation ($\beta_a \approx 7$) above a critical threshold ($x_{\text{crit}} \approx 3$). The β_a/x_{crit} ratio primarily determined fit quality, with the final model achieving an root mean square error (RMSE) of 0.035 for HCT116 cells treated with oxaliplatin.

To determine the threshold value for assessing TPR candidate efficacy, we first calculated the mean D_n value for the control group. We then employed an iterative adjustment approach to identify the threshold value using the Θ function. The initial parameters for the model were set as follows: $x_{\text{crit}} \approx 3$, $\beta_a \approx 7$, and $\ln(E/\overline{E}) \approx 2$. Initially, the D_n threshold value ($D_{n,crit}$) was set to the mean D_n value of the control group. Using the Θ function, we calculated $D_{n,crit}$ incorporating these model parameters. $D_{n,crit}$ was then decreased incrementally by 0.001 until Θ approached a value just below 0.99. The final $D_{n,crit}$ threshold value obtained was approximately 2.33, with a corresponding Θ value of 0.99.

For the optimization for Θ_b vs. Θ_a, we used cell confluence data of A2780 cells treated with the combination of TPR (celecoxib) and chemotherapy (paclitaxel) rather than PWS microscopy data. We utilized the experimental data of cell death probability for paclitaxel alone (Θ_{pac}) and the combination treatment (Θ_{combo}) to fit the model parameters. The optimization function minimized the error between the predicted and experimental Θ_{combo} values $f(\vec{p}) = \sum_{i=1}^{n} [\Theta_b(\Theta_{\text{pac},i}, \vec{p}) - \Theta_{\text{combo}}(\Theta_{\text{pac},i})]^2$, where Θ_b is the model-predicted cell death probability for the combination treatment, $\vec{p}$ are the input model parameters $D_{\text{control}}, D_{\text{TPR}}, \beta_a, \ln(E/\overline{E})$, and D_{control} and D_{TPR} are the initial D_n values for control and TPR-treated cells, respectively. We employed the minimization with bounds of 1 to 30 for β_a and -6 to 4 for $\ln(E/\overline{E})$. This optimization yielded $\beta_a \approx 3$ and $\ln(E/\overline{E}) \approx 2$ for the A2780 cells, with an RMSE of 0.16. The fit was primarily dependent on β_a, with very little impact of $\ln(E/\overline{E})$. These optimized parameters were then used to generate both exact and approximate Θ_b curves for comparison with experimental data.

Patient-Derived Xenograft (PDX) Tumor Models. The following research protocol was approved by Northwestern University's institutional review board. High Grade Serous Ovarian Cancer (HGSOC) patient-derived tissue samples were obtained from chemotherapy-naive ovarian cancer patients following surgical resection at Prentice Women's Hospital of Northwestern University, from September 2013 to June 2014, with patient consent for tissue acquisition. For this study, we utilized a cryopreserved de-identified patient-derived tissue sample (OVCA10) at its fourth generation (passage 4). Tumor fragments measuring 2 × 2 mm were subcutaneously engrafted into the right flank of non-obese diabetic/severe combined immunodeficient (NOD/SCID) gamma (NSG) mice (Jackson Laboratory). Once the engrafted PDX tumors reached a volume of 150–200 mm^3, the mice were randomized into five experimental groups: celecoxib vehicle control, paclitaxel vehicle control, 25 mg/kg celecoxib, 1.7 mg/kg paclitaxel, and a combination group (25 mg/kg celecoxib and 1.7 mg/kg paclitaxel). Celecoxib or vehicle was administered daily via oral gavage, while paclitaxel or vehicle was administered twice weekly (Mondays and Thursdays) via intraperitoneal (IP) injection. The experiment spanned 4 weeks, with tumor size and body weight monitored biweekly. Tumor dimensions were measured using digital calipers, recording the longest diameter (length, l) and the diameter perpendicular to it (width, w). Tumor volume (V) was calculated using the formula $V = l \cdot w^2/2$. At the end of the 4-week treatment period, the mice were euthanized, and PDX tumors were collected and preserved in 10% formalin. For fitting the adaptation model to experimental data, non-linear least squares optimization was performed using `scipy.optimize.curve_fit`.

Tumor Processing and Histopathology. PDX tumors were fixed in 10% formalin and embedded in paraffin and delivered to the Northwestern Pathology Core in Chicago, IL. The tumors were sliced in 4 um thickness and picked up onto microscope slides. The slides were stained using hematoxylin and eosin (H&E) and immunohistochemistry (IHC) for TNF-a (ab6671) 1:300 and Il-6 (ab6672) 1:400. Immunohistochemical studies were performed on 5-micron FFPE sections on charged slides by using protocol F, Leica Bond-Max Autostainer. The following steps were employed by using Leica Bond Polymer Refine Detection Kit (DS9800) with first Peroxide Block for 5 minutes, followed by use of antibodies for 15 minutes. Post Primary and Polymer steps were applied for 8 minutes, DAB Refine mixes for 10 minutes, followed by Hematoxylin for 5 minutes. All slides were rehydrated through alcohol and xylene, mounted, and cover slipped. Stained slides were scanned using digital slide scanner, Nanozoomer 2.0-HT by Hamamatsu, which mainly converts stained slides at high speed into high-definition digital images. Standard-size (26 mm × 76 mm) slides, with fully automatic operation mode, were scanned with 20× (0.46 µm/pixel). Scanned images were then viewed with NDP.view2 next-generation viewer software for further pathologic review and quantitative analysis. Two pathologists individually assessed immune cell infiltration in five different regions of each H&E slide. Semi-quantitative analysis was performed for IHC images using ImageJ adapted from an existing protocol.

Flow Cytometry. Flow cytometry on A2780 cells was performed at the Northwestern University Flow Cytometry Core using a BD LSRII instrument, and data were analyzed using FlowCytometryTools 0.4.5, an open-source Python software package. Flow cytometry for HCT116 cells was performed on BD FACSDiscover S8 Cell Sorter located at the Single Cell Genomics Core at Northwestern University in Evanston, IL and analyzed using FlowJo software.

Apoptotic induction was measured using CellEvent Caspase-3/7 Green Detection Reagent and Hoechst 33342 (#C10423 and #H3570; ThermoFisher Scientific, Waltham, MA). Cells were trypsinized, stained with 2 µM Caspase-3/7 and 4 µM Hoechst 33342 for 30 minutes, then processed for flow cytometry. Following staining, cells were centrifuged for 5 minutes at 500×g, washed with PBS, and resuspended in 1 mL of fresh media.

For cell death analysis with flow cytometry, cell culture media and PBS wash (Thermo Fisher Scientific, #14190–144) were collected to account for floating cells. Cells were then trypsinized (Gibco, #25200–056) to collect remaining adherent cells. This collection of floating and trypsinized cells were centrifuged at 500 g for 5 minutes. Pellets were re-suspended in 5 mL of PBS before centrifugation at 500 g for 5 minutes. After removing the supernatant, cells were resuspended in 1 mL of PBS. Cells were passed through a cell strainer before propidium iodine staining (ThermoFisher Scientific, P3566) for 15 minutes on ice.

Mock-stained cells were collected under the same preparation conditions. We collected data from 20,000 cells by forward and side scattering channels for each group, setting illumination intensities to minimize autofluorescence from unstained cells. Gates were established to minimize false positives from unstained cells (less than 0.1% of total). The percentage of apoptotic cells was assessed as the ratio of Caspase-3/7 positive cells to the population of Hoechst 33342 positive cells. Error bars represent uncertainty based on a ±10% change in gating thresholds.

Chromatin Scanning Transmission Electron Microscopy (ChromSTEM). Chromatin Scanning Transmission Electron Microscopy (ChromSTEM) was employed to assess the characteristics of packing domains in HCT116 cells, adhering to established protocols

(2). For electron microscopy preparation, HCT116 cells underwent standard fixation and staining procedures. Briefly, cells were fixed in 2.5% glutaraldehyde in 0.1 M sodium cacodylate buffer, post-fixed with 1% osmium tetroxide, dehydrated through a graded ethanol series, and embedded in Epon resin. Ultrathin sections (70–90 nm) were cut using a diamond knife on an ultramicrotome and subsequently mounted on copper grids. The sections were stained with uranyl acetate and lead citrate prior to imaging. ChromSTEM imaging was performed using a Thermo Fisher Talos F200X G2 transmission electron microscope operating at an accelerating voltage of 200 kV in STEM mode. Images were acquired with a pixel size of 1 nm and a dwell time of 20 µs. The chromatin packing domain parameters, including the packing domain scaling exponent (D_{PD}), the average packing domain chromatin volume concentration ($\mathrm{CVC}_{\mathrm{PD}}$), the average packing domain volume packing efficiency (A_v), the radius of the domain (r_{PD}), and lower bound where the domain deviates from the scaling exponent (r_{fiber}), were quantified using custom MATLAB scripts (2).

The genomic size of a packing domain, N_{PD}, was calculated by determining the number of voxels within the packing domain and multiplying by the amount of chromatin contained within a single voxel, expressed in base pairs (bp). The genomic size of chromatin within individual ChromSTEM voxels was estimated based on an assumed DNA density and voxel volume. The DNA density (ρ_{DNA}) was assumed to be 2 g/cm^3, corresponding to unhydrated DNA, which reflects the condition that the highest intensity observed in tomograms represents 100% unhydrated DNA. The voxel mass, measured in base pairs (N_{voxel}), was computed using $N_{\mathrm{voxel}} = (V_{\mathrm{voxel}} \cdot \rho_{\mathrm{DNA}})/(2 \cdot M_{\mathrm{nucleotide}} \cdot 1.660 \times 10^{-24})$ (1). Here, the voxel volume (V_{voxel}) was based on an assumed ChromSTEM voxel size (l_{voxel}) of 2 nm, converted to cm^3. The molecular weight of a nucleotide ($M_{\mathrm{nucleotide}}$) was assumed to be 325 Daltons, and the atomic mass unit conversion factor was 1.660×10^{-24} g/AMU. To calculate N_{PD}, the genomic size of the packing domain, we applied the fractal scaling equation $N_{\mathrm{PD}} = (r_{\mathrm{PD}}/l_{\mathrm{voxel}} \cdot A_v)^{D_{\mathrm{PD}}} \cdot N_{\mathrm{voxel}}$ (2). Sensitivity analyses were conducted using the 25th, 50th, and 75th percentiles for D_{PD}, N_{PD}, and A_v to evaluate parameter variability. For additional analyses, mean values from the HCT116 cell population were employed: $D_{\mathrm{PD}} = 2.6$, $\mathrm{CVC}_{\mathrm{PD}} = 0.275$, $A_v = 0.6$, and $N_{\mathrm{PD}} = 380$ kbp. Median values were used for $r_{\mathrm{PD}} = 110$ nm and $r_{\mathrm{fiber}} = 10$ nm.

Statistical Analysis. All statistical analyses were conducted in Python using SciPy. To assess statistical significance across multiple conditions, pairwise comparisons using Welch's t-test between a reference (control) condition and all other conditions were performed within each group. This approach was applied in several contexts, including comparisons of nuclear D_n values between treated and control cell populations, assessments of cell viability between different treatment conditions, and analyses of tumor volumes in PDX models across treatment groups. Mann-Whitney test was employed for PDX histology data. We considered P-values less than 0.05 as statistically significant, and significance levels were denoted as follows: $^{*}P<0.05$, $^{**}P<0.01$, and $^{***}P<0.001$.

In visualizing D_n distributions, violin plots were used, with dashed lines indicating the 25th, 50th (median), and 75th percentiles. The width of each violin reflects the frequency distribution of D_n values. Unless otherwise specified, error bars in figures represent the standard error of the mean (SEM). For error propagation in calculations, the uncertainties package was used to automatically propagate standard deviations through all relevant steps.

Computational Resources and Code Availability. All computational analyses were conducted using MATLAB and Python 3.7.1, with NumPy 1.15.4, SciPy 1.1.0, and pandas 0.23.4. Data visualization was performed using Matplotlib 3.3.2 and Seaborn 0.11.0. Custom scripts developed for data analysis and visualization are available at https://github.com/BackmanLab/CDA_Paper.

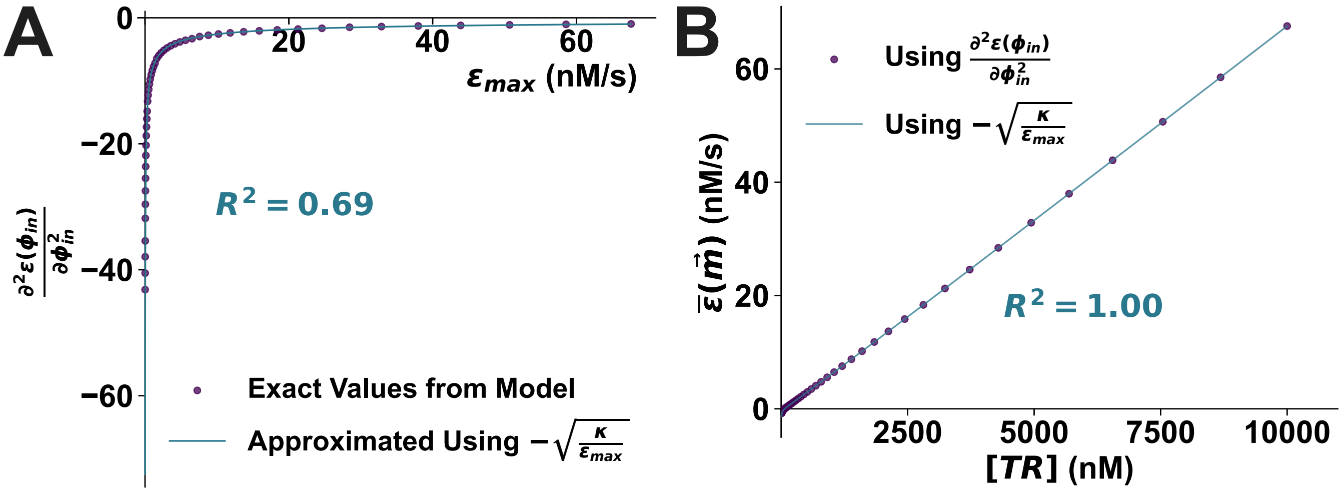

Fig. S1. Analytical approximation for $\bar{\varepsilon}$ and its second derivative in the Taylor expansion using κ closely matches exact values. (A) Plot of the second derivative $\left(\frac{\partial^2 \varepsilon(\phi_{\text{in}})}{\partial \phi_{\text{in}}^2}\right)$ as a function of the maximum expression (ε_{max}) for the input vector of molecular factors ($\vec{m}$). The curvature parameter κ is obtained by fitting to the exact second derivative values. (B) Comparison of the average expression rate $\bar{\varepsilon}$ computed directly using the second derivative versus the κ-based analytical approximation.

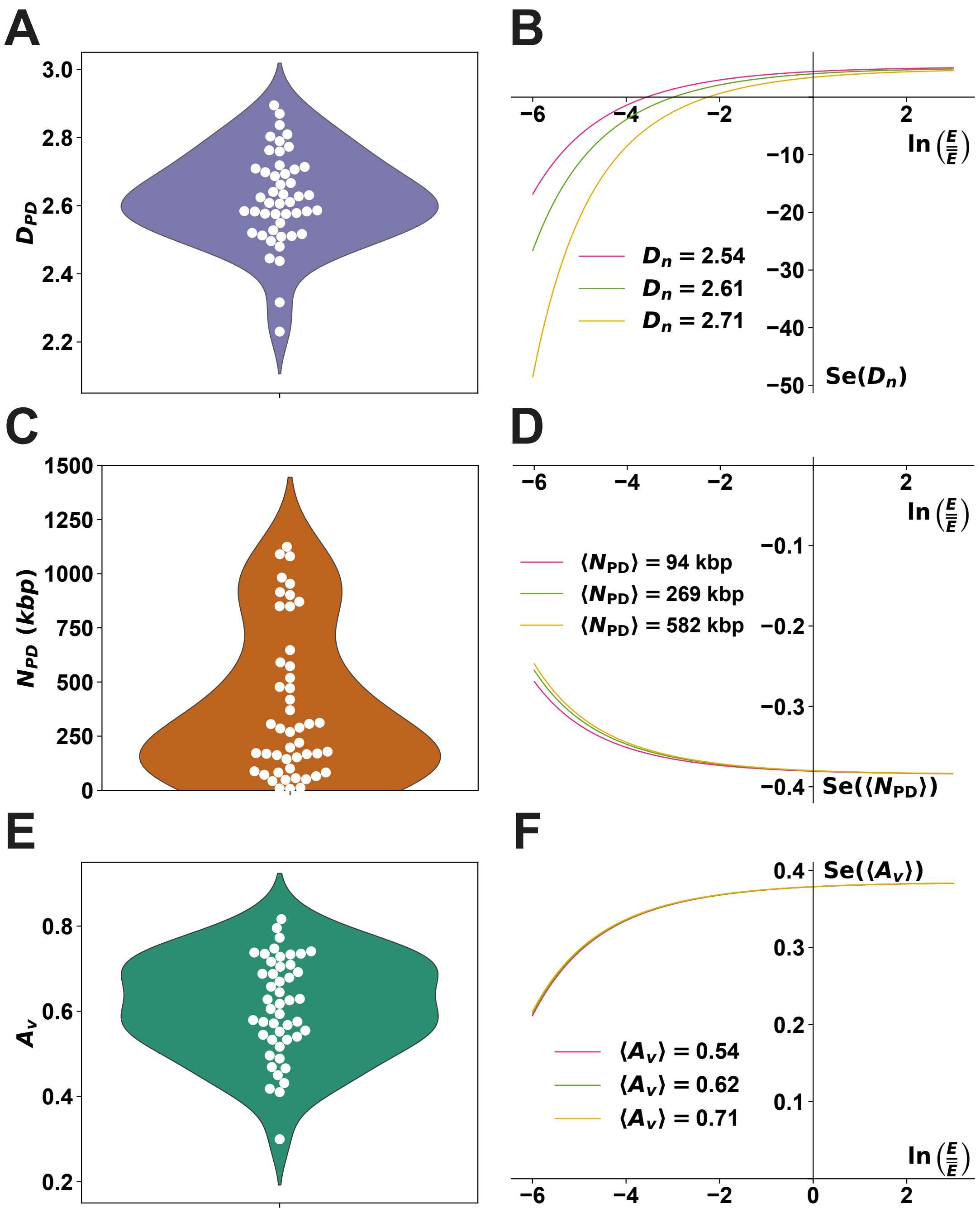

Fig. S2. CPMC parameters and their effects on gene expression sensitivity. (A) Distribution of scaling within packing domains (D_{PD}) in HCT116 cells. (B) Sensitivity of gene expression to changes in packing domain scaling ($Se(D_{\text{PD}})$) as a function of relative gene expression ($\ln(E/\overline{E})$) for different D_{PD} values. (C) Distribution of packing domain sizes (N_{PD}) in HCT116 cells. (D) Sensitivity of gene expression to changes in packing domain size ($Se(N_{\text{PD}})$) as a function of relative gene expression for different N_{PD} values. (E) Distribution of packing domain packing efficiency factors (A_v) in HCT116 cells. (F) Sensitivity of gene expression to changes in average nuclear crowding ($Se(A_v)$) as a function of relative gene expression for different A_v values. Sensitivities were calculated using the CPMC model with parameters derived from ChromSTEM analysis of HCT116 cells. Note the different y-axis scales, indicating that $Se(D_{\text{PD}})$ has the largest magnitude among the three parameters.

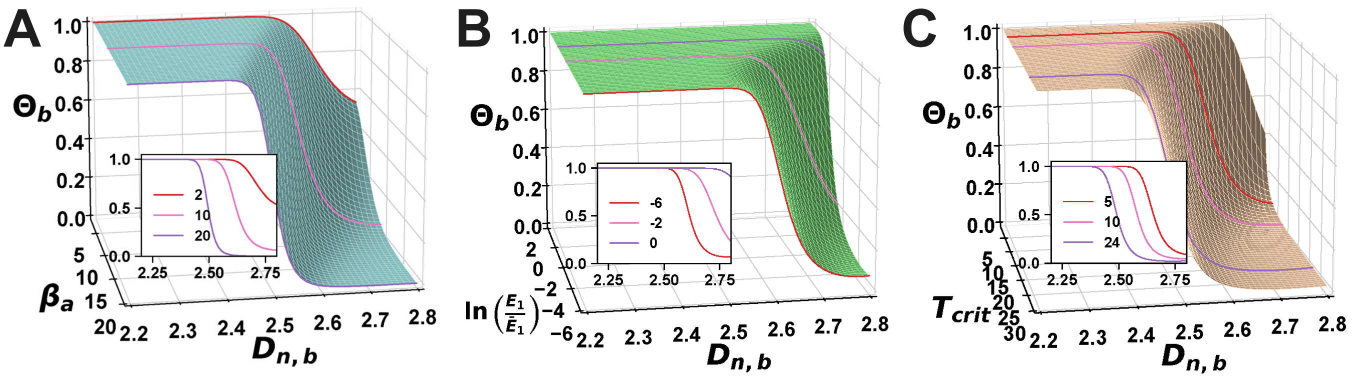

Fig. S3. Parameter scans for selected free parameters in the CDA model. (A) Cell death probability (Θ) as a function of chromatin packing scaling ($D_{n,b}$) and upregulation (β_a). Inset shows individual curves for select β_a values. Fixed parameters: $x_{\text{crit}} = 5$, $\ln(E_1/\overline{E}_1) = -5.5$, $t_{\text{crit}} = 7$ hours. (B) Θ as a function of chromatin packing scaling ($D_{n,b}$) and relative initial gene expression ($\ln(E_1/\overline{E}_1)$). Inset shows individual curves for select $\ln(E_1/\overline{E}_1)$ values. Fixed parameters: $\beta_a = 10$, $x_{\text{crit}} = 5$, $t_{\text{crit}} = 7$ hours. (C) Θ as a function of $D_{n,b}$ and critical decision time (t_{crit}). Inset displays curves for select t_{crit} values. Fixed parameters: $\ln(E_1/\overline{E}_1) = -5.5$, $\beta_a = 10$, $x_{\text{crit}} = 5$. All plots were generated with $D_{n,a} = 2.6$ and $D_{n,b}$ ranging between 2.2 and 2.8.

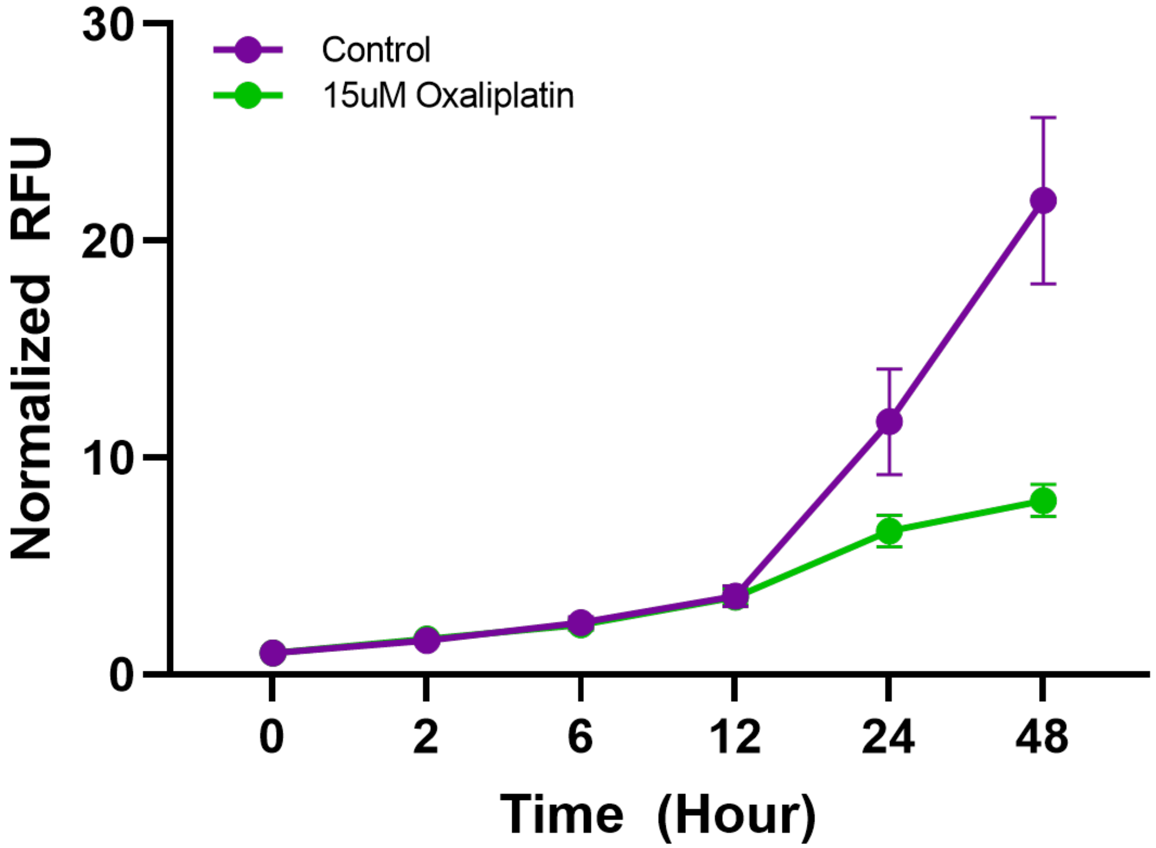

Fig. S4. Cell viability analysis of HCT116 cells treated with oxaliplatin. The line plot shows the time course of normalized relative fluorescence units (RFU) measuring cell viability in control (purple) and 15 µM oxaliplatin-treated (green) HCT116 cells over 48 hours. Fluorescence intensity was measured using a viability assay at 0, 2, 6, 12, 24, and 48 hours post-treatment. Error bars represent standard error of the mean from three independent experiments.

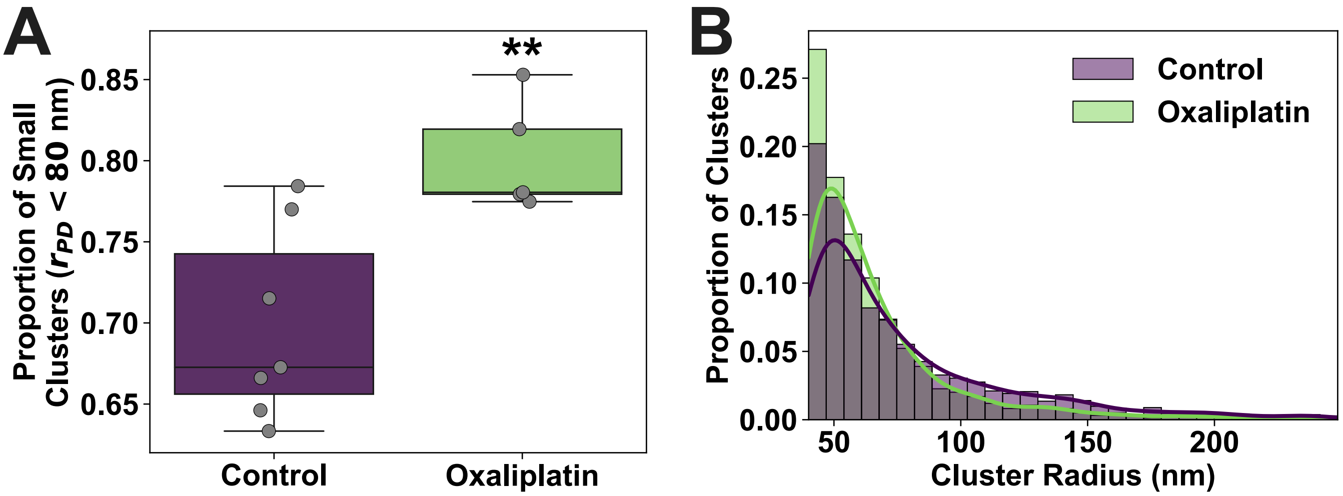

Fig. S5. Colon cancer cells that survive oxaliplatin treatment have a larger proportion of small domains compared to untreated cells. (A) The proportion of small clusters (<80 nm in radius) for control and 48 hour oxaliplatin treated HCT116 cells. Significance level: $^{**}P<0.01$ (unpaired two-tailed t-test with unequal variance, relative to control). (B) PDF of cluster sizes in control and oxaliplatin-treated cells.

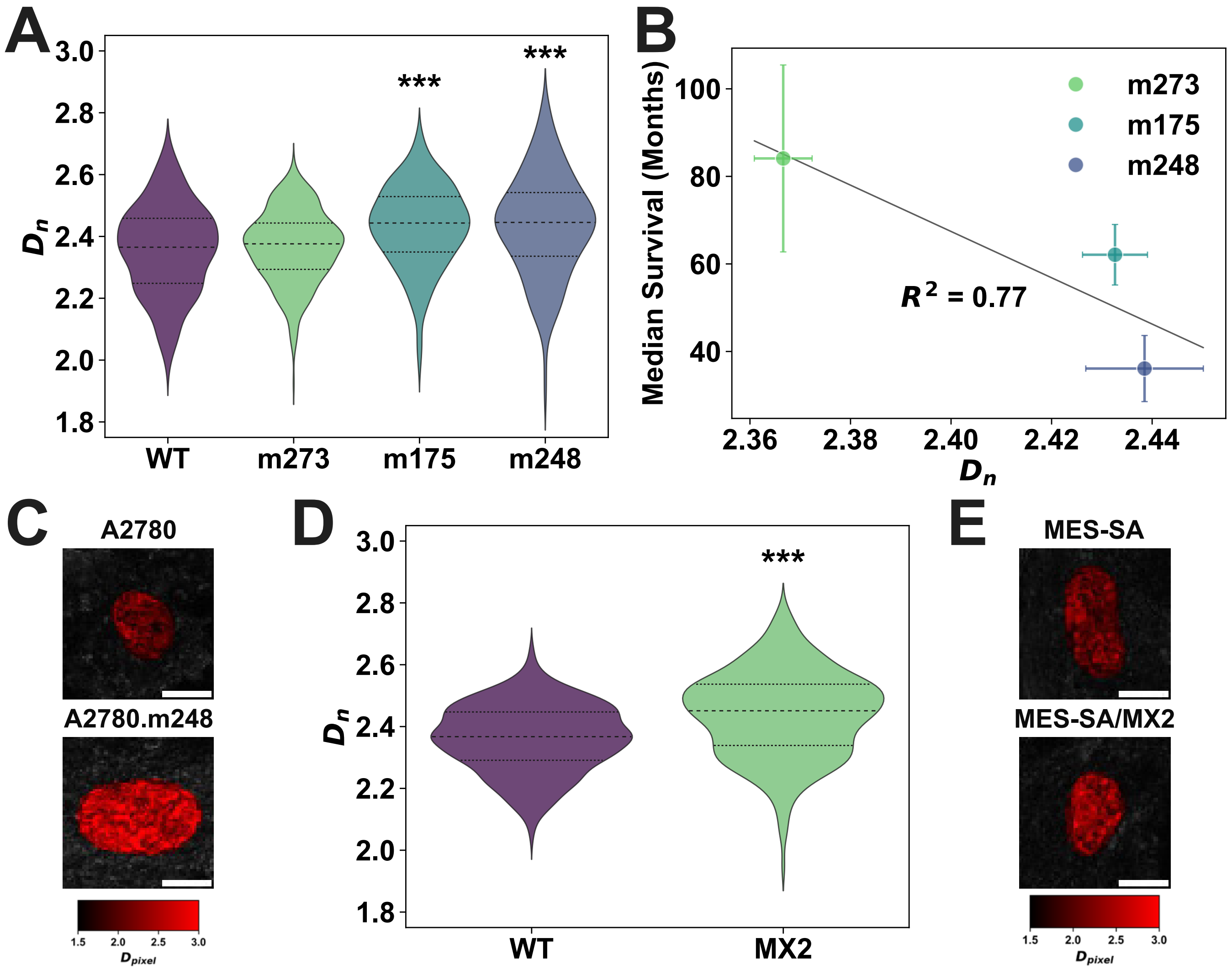

Fig. S6. Cancer cells with selective resistance to chemotherapy have higher D_n. (A) Violin plots showing D_n distributions for A2780 wild-type (WT) and TP53 mutant subclones (m273, m175, m248) under normal growth conditions. Significant increases in D_n are observed in m175 and m248 compared to WT ($P<0.001$). (B) Correlation between median survival of high-grade serous epithelial ovarian carcinoma patients (TCGA data) and D_n for cells with matching TP53 mutations. A strong negative correlation is observed ($R^2 = 0.77$). (C) Representative PWS microscopy images of A2780 WT and TP53 mutant A2780.m248 cells. (D) Violin plots showing increased D_n in chemoresistant MES-SA/MX2 subclone compared to chemosensitive MES-SA WT cells ($P<0.001$). (E) Representative PWS images of MES-SA WT and MES-SA/MX2 cells. For A and D, dashed lines in violins represent 75th percentile, median, and 25th percentile. For C and E, pseudocolor represents D_n values, with brighter red indicating higher D_n. Significance level: $^{***}P<0.001$ (unpaired two-tailed t-test with unequal variance, relative to control). Scale bars: 5 µm.

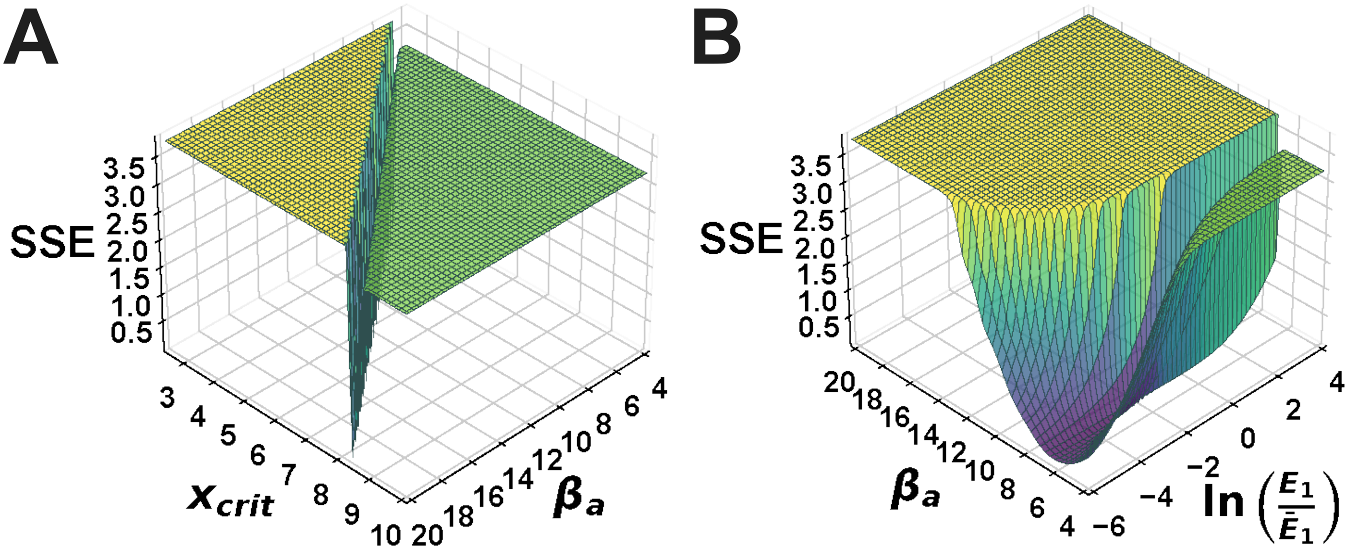

Fig. S7. The relationship between death probability Θ and D_n depends on the critical threshold x_{crit} and level of upregulation β_a. (A) 3D plot showing sum of squared errors (SSE) between the CDA model and experimental data from HCT116 cells as a function of β_a and x_{crit}. (B) 3D plot showing SSE between the CDA model and experimental data from HCT116 cells as a function of β_a and $\ln(E_1/\overline{E}_1)$.

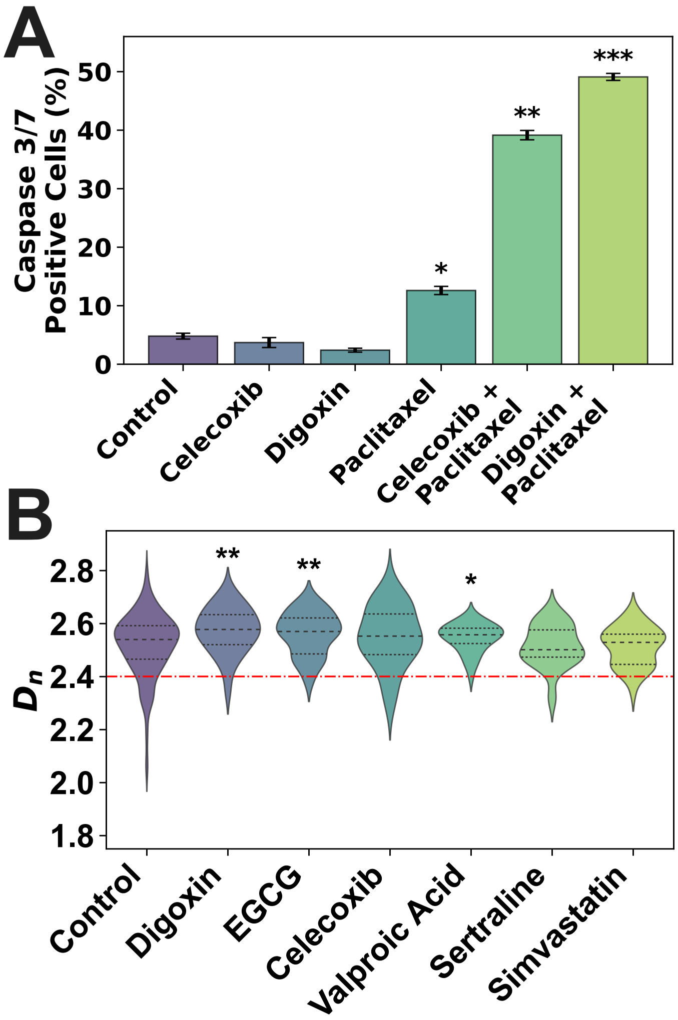

Fig. S8. TPRs enhance chemotherapy-induced apoptosis without inducing significant cell death on their own and have minimal effects on non-cancerous cells. (A) Bar graph shows the percentage of Caspase 3/7 positive A2780 cells after 48 hours of treatment with control, celecoxib alone, digoxin alone, paclitaxel alone, and combinations of celecoxib or digoxin with paclitaxel. Error bars represent standard error of the mean from three independent experiments. Significance levels compared to paclitaxel alone: $^{*}P<0.05$, $^{**}P<0.01$, $^{***}P<0.001$ (determined using unpaired t-test with unequal variance). (B) Violin plots show D_n distributions for various treatments, including control and potential TPRs (digoxin, EGCG, celecoxib, valproic acid, sertraline, and simvastatin). Dashed lines within the violins represent the interquartile range. Significance levels compared to control: $^{*}P<0.05$, $^{**}P<0.01$ (determined using unpaired t-test with unequal variance).

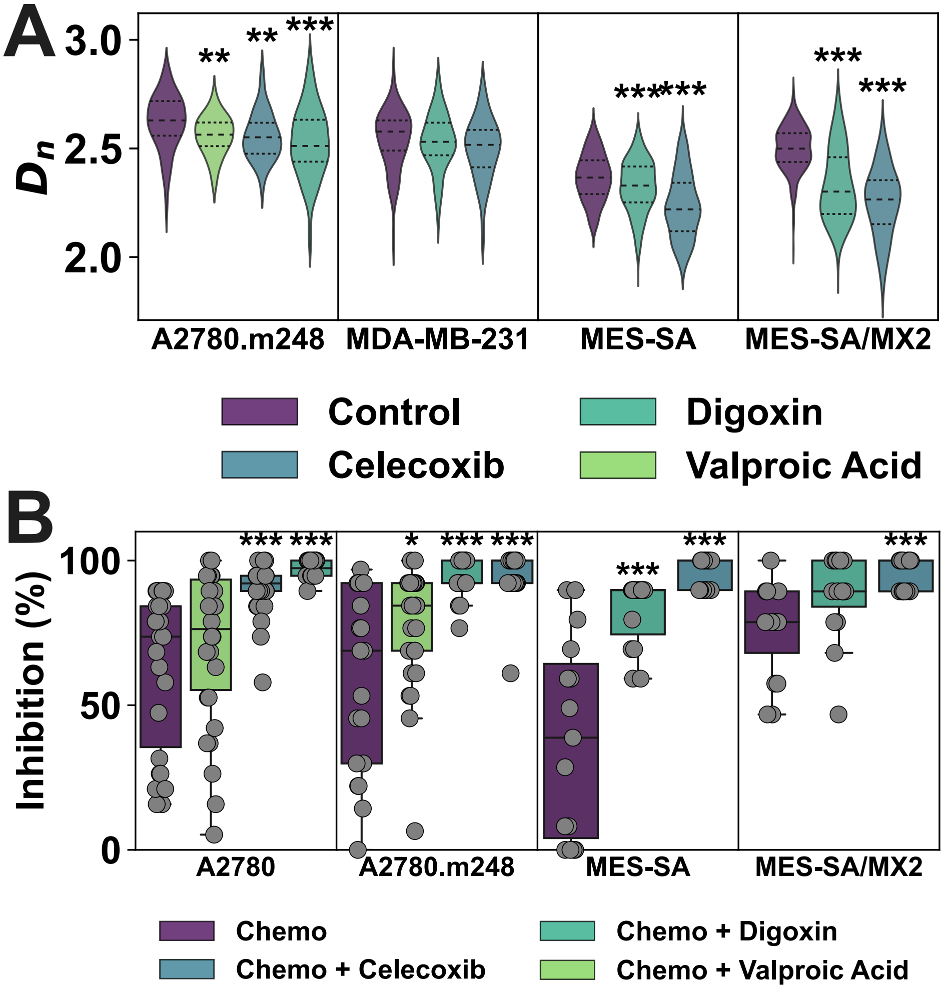

Fig. S9. Transcriptional Plasticity Regulators (TPRs) reduce D_n and increase chemotherapy-induced cell death in multiple cancer cell lines. (A) Violin plots show the distribution of D_n values for different cancer cell lines (A2780.m248, MDA-MB-231, MES-SA, and MES-SA.MX2) treated with various TPRs (celecoxib, valproic acid, and digoxin) compared to untreated controls. Cells were treated with TPRs for 30 minutes prior to PWS imaging. The width of each violin represents the frequency of D_n values, while the internal lines indicate the quartiles. Asterisks denote statistical significance compared to the control group ($^{**}P<0.01$, $^{***}P<0.001$; unpaired t-test with unequal variance). (B) Box plots show percent inhibition of cell growth for A2780, A2780.m248, MES-SA, and MES-SA.MX2 cells treated with chemotherapy alone or in combination with TPRs. Treatments include paclitaxel, oxaliplatin, or docetaxel alone, and combinations with valproic acid, celecoxib, digoxin, or aspirin. Each data point represents an independent experiment. The box extends from the 25th to 75th percentiles, with the line in the middle representing the median. Whiskers show the minimum and maximum values. Significance levels compared to chemotherapy alone: $^{*}P<0.05$, $^{**}P<0.01$, $^{***}P<0.001$ (determined using unpaired t-test with unequal variance).

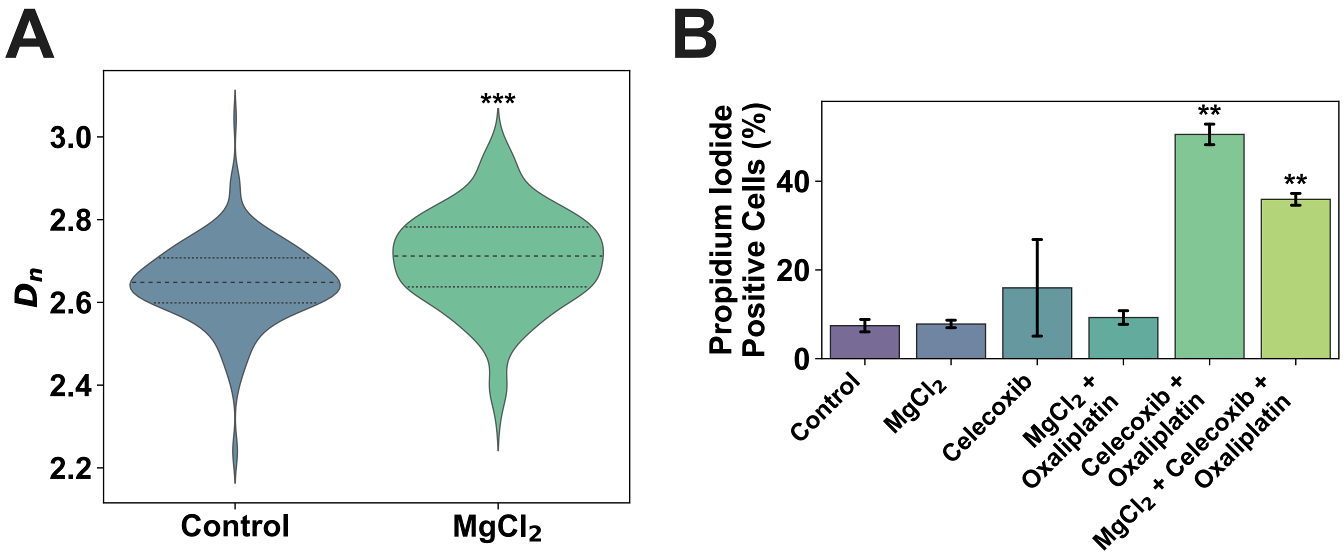

Fig. S10. Magnesium chloride ($MgCl_2$) treatment increases D_n and reduces synergy between D_n-lowering celecoxib and oxaliplatin. (A) Violin plots show D_n distributions of cells treated with 10 µM $MgCl_2$ for one hour compared to control. Dashed lines within the violins represent the interquartile range. Significance level compared to control: $^{***}P<0.001$ (determined using unpaired t-test with unequal variance). (B) Bar graph shows the percentage of propidium iodide positive HCT116 cells after 48 hours of treatment with control, $MgCl_2$ alone, celecoxib alone, and combinations of $MgCl_2$ or celecoxib with oxaliplatin. Error bars represent standard error of the mean from three independent experiments. Significance level compared to control: $^{**}P<0.01$ (determined using unpaired t-test with unequal variance).

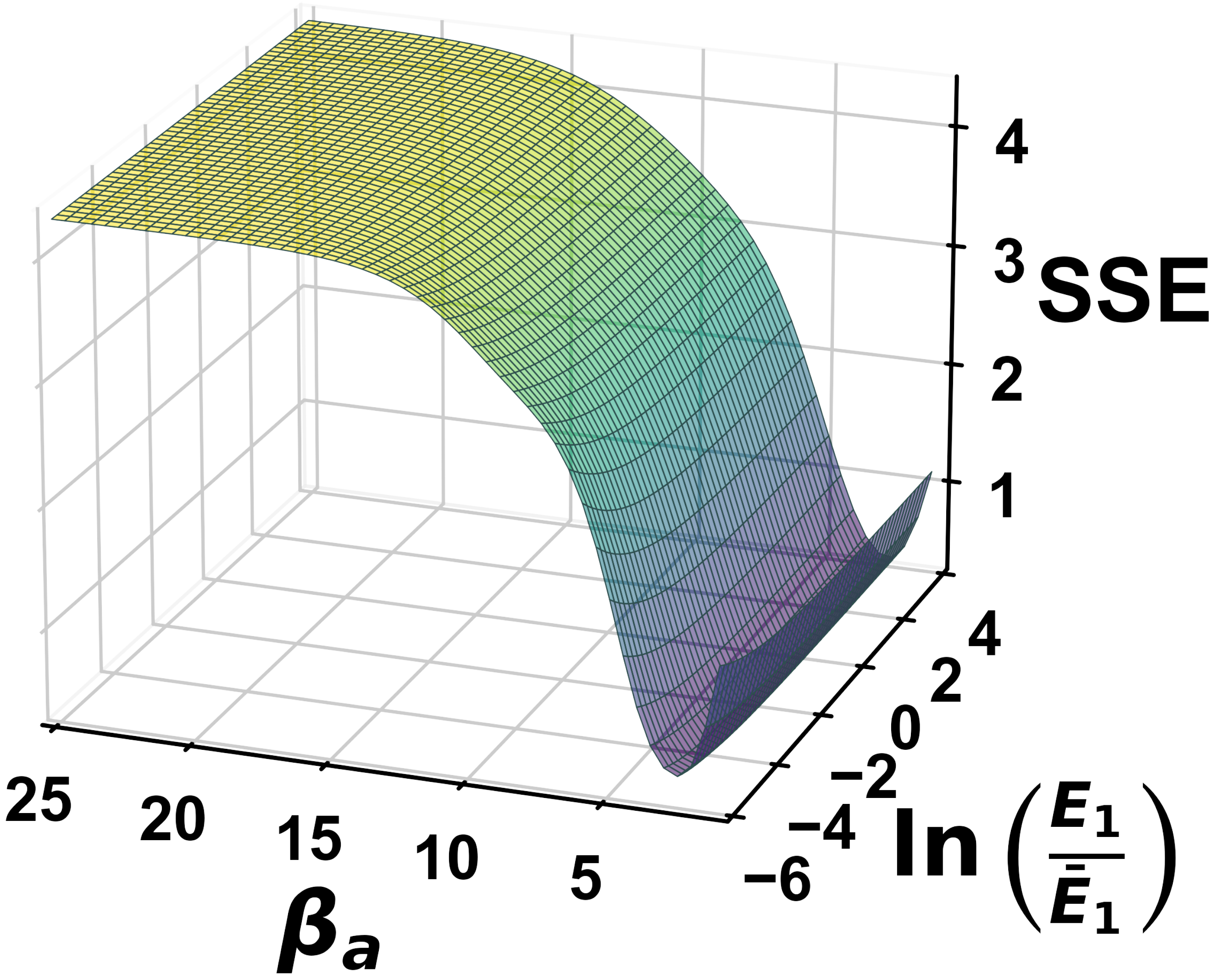

Fig. S11. Optimization of CDA model parameters for $\Theta_b(\Theta_a)$. Heat map showing the sum of squared errors (SSE) of the CDA model fit as a function of gene upregulation β_a and initial relative expression rate $\ln(E_1/\overline{E}_1)$. The optimization was performed using experimental data from A2780 cells treated with paclitaxel and celecoxib. The plot demonstrates that changes in β_a most strongly influence the quality of fit to experimental data, with negligible effects of the initial relative expression rates.

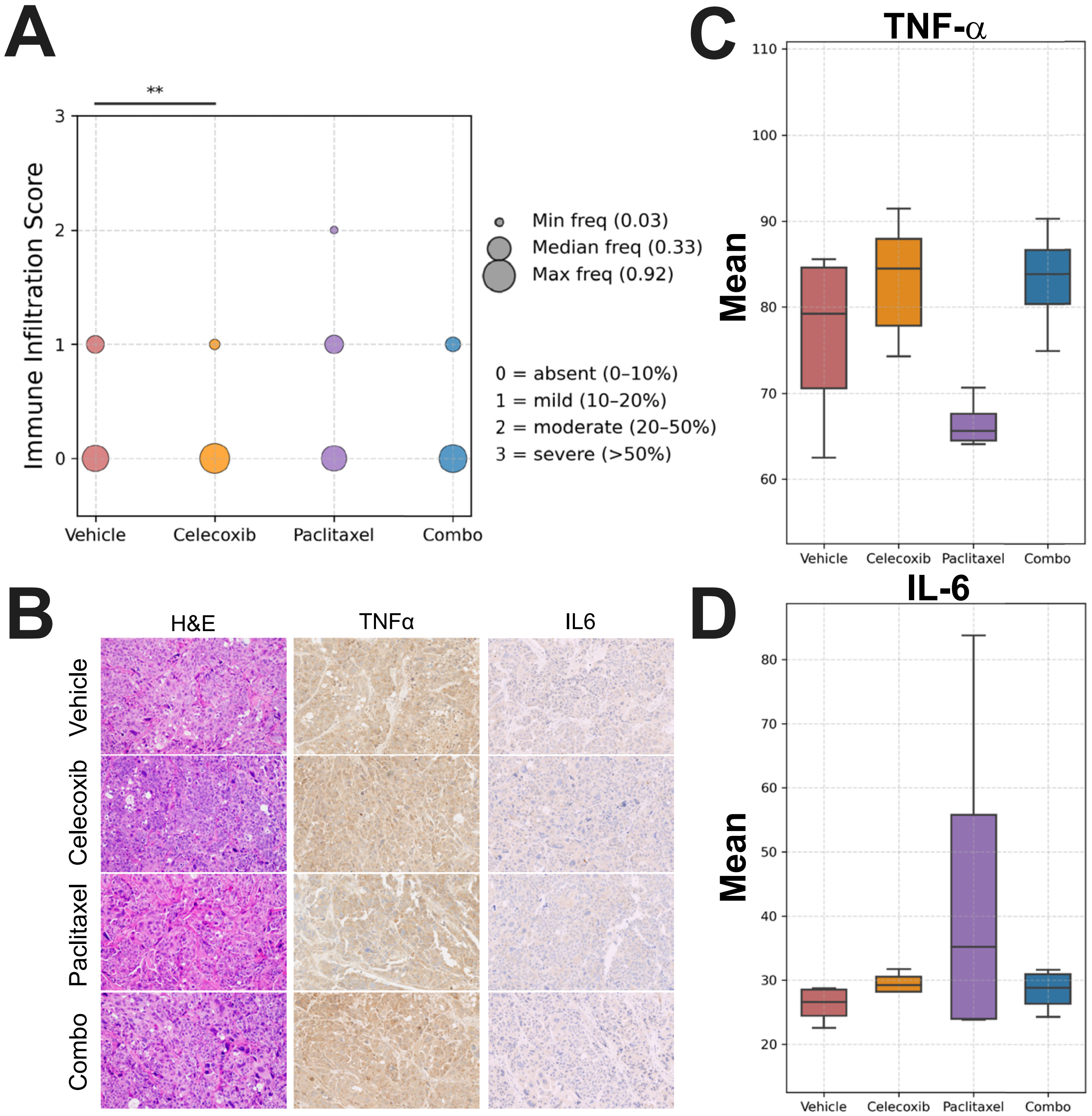

Fig. S12. Analysis of PDX tumor sections reveals minimal effects of celecoxib on immune infiltration and COX-2 dependent inflammatory markers. (A) Dot plots showing Tumor infiltrating lymphocyte (TIL) scoring between treatment groups. All treatment groups show absent-to-mild scoring. Significance level: $^{**}P<0.01$ (Mann-Whitney test). (B) Representative images of H&E, TNF-α, and IL-6 stained PDX tumor sections at 20× magnification. (C) Box plot depicting mean intensity per pixel of IHC staining for TNF-α (no statistical differences as determined by a Mann-Whitney test). (D) Box plot depicting mean intensity per pixel of IHC staining for IL-6 (no statistical differences as determined by a Mann-Whitney test).

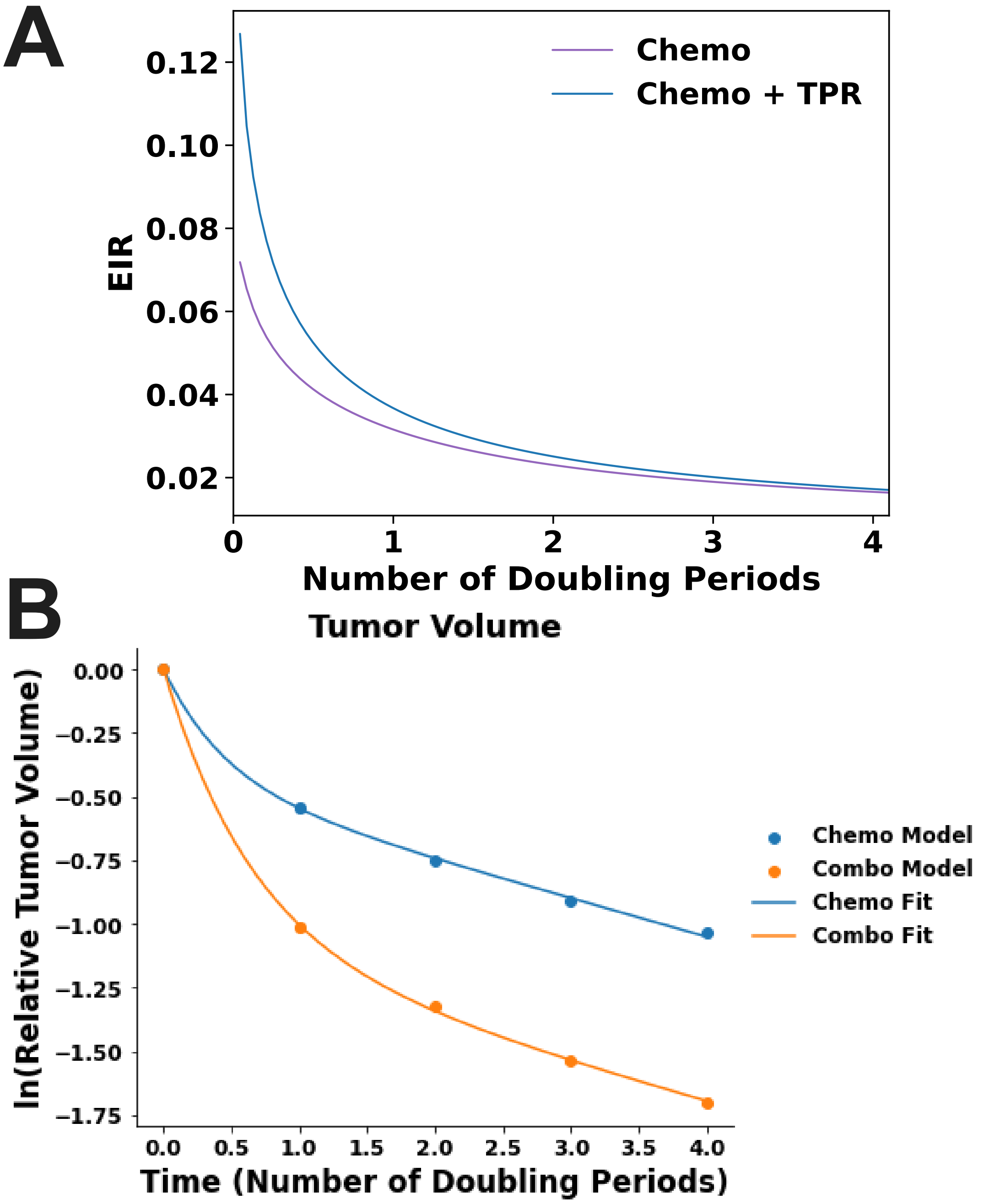

Fig. S13. CDA model predictions of Effective Inhibition Rate (EIR) and change in tumor volume using *in vitro* experiment results. (A) The graph shows the predicted EIR as a function of the number of cell doubling periods for chemotherapy treatment alone (purple line) and chemotherapy combined with TPR (blue line). EIR represents the cumulative cancer cell death at a given time point. (B) The relative tumor volume was predicted based on a changed in D_n for cells treated with chemotherapy alone (blue) vs. combination treatment of chemotherapy with a TPR (orange). CDA model predictions (dots) were fit with the adaptation model (Eq. 58; lines).

Table S1. Numerical values of Macromolecular Crowding (MC) model parameters.

Parameter	Description	Value for $\phi = 0$
V_{cell}	Volume of a typical HeLa cell	500 µm^3
L_{DNA}	Number of DNA base pairs in a diploid human cell	6 × 10^9 bp
$L_{DNA,half}$	One-half of the total length of genomic DNA	1 m
l_{bp}	Length of one base pair	0.34 nm
$N_{bp/turn}$	Number of base pairs per turn of DNA	10
r_{DNA}	Radius of the DNA molecule	1 nm
r_{TF}	Radius of TF (spherical approximation)	4.0 nm
$r_{Pol\ II}$	Radius of Pol II (spherical approximation)	5.4 nm
$r_{crowder}$	Radius of nuclear crowding agents	3.0 nm
D_{TF}	Diffusion coefficient of TF	3 µm^2/s
$D_{Pol\ II}$	Diffusion coefficient of Pol II	2 µm^2/s
$D_{1,TF}$	One-dimensional diffusion coefficient of TF on DNA	0.046 µm^2/s
$D_{1,Pol\ II}$	One-dimensional diffusion coefficient of Pol II on DNA	0.03 µm^2/s
k_t^{ns}	Association rate constant for nonspecific TF-DNA binding	4.9×10^4 µM^{-1}s^{-1}
k_f^{ns}	Association rate constant for nonspecific Pol II-DNA binding	3.6×10^4 µM^{-1}s^{-1}
k_o^{ns}	TF-DNA nonspecific dissociation rate	4.9×10^4 s^{-1}
k_b^{ns}	Pol II-DNA nonspecific dissociation rate	3.6×10^4 s^{-1}
$K_{D,TF}^{ns}$	Dissociation constant for nonspecific TF-DNA binding	1 µM
$K_{D,Pol\ II}^{ns}$	Dissociation constant for nonspecific Pol II-DNA binding	1 µM
k_t	Association rate constant for TF-promoter (O) binding	0.05 nM^{-1}s^{-1}
k_f	Association rate constant for Pol II-Complex I binding	0.03 nM^{-1}s^{-1}
k_o	TF-promoter (O) dissociation rate	1.0 s^{-1}
k_b	Pol II-Complex I dissociation rate	0.6 s^{-1}
$K_{D,TF}$	Dissociation constant for TF-O (promoter) binding	1 nM
$K_{D,Pol\ II}$	Dissociation constant for Pol II-O (promoter) binding	1 nM
k_m	Rate of pre-mRNA production	0.007 s^{-1}
γ	Nuclear export rate of mRNA	8×10^{-4} s^{-1}
ν	mRNA degradation rate	3×10^{-4} s^{-1}
$[TF]_{tot}$	Total concentration of TF	30 nM
$[Pol\ II]_{tot}$	Total concentration of Pol II	30 nM
$[O]_{tot}$	Total concentration of O (promoters)	30 nM
$[D]_{tot}$	Total concentration of DNA basepairs	20 µM
**Coefficients for cubic fit from Brownian Dynamics simulations (10)**		
$\alpha_{TF}, \beta_{TF}, \gamma_{TF}$	Coefficients for TF	-2.83, 3.87, -4.11
$\alpha_{Pol\ II}, \beta_{Pol\ II}, \gamma_{Pol\ II}$	Coefficients for Pol II	-3.89, 7.72, -7.72
**Coefficients for ϕ-influenced free energies from Monte Carlo simulations (10)**		
$f_{cro,TF}(\phi)$	TF crowding free energy	$-3.2\phi - 2.0\phi^2$
$f_{cro,Pol\ II}(\phi)$	Pol II crowding free energy	$-3.7\phi - 2.7\phi^2$
$f_{cro,Pol\ II,s}(\phi)$	Pol II sliding crowding free energy	$-2.6\phi - 4.6\phi^2$
$f_{ba,TF}(\phi)$	TF barrier free energy	$2.5\phi^2$
$f_{ba,Pol\ II}(\phi)$	Pol II barrier free energy	$3.1\phi^2$
$f_{ba,Pol\ II,s}(\phi)$	Pol II sliding barrier free energy	$0.1\phi^2 + 9.2\phi^3$

Table S2. Numerical values of Chromatin-Dependent Adaptability (CDA) model parameters.

Parameter	Description	Value
$\langle D_{\text{PD}} \rangle$	Average packing domain scaling exponent	2.6
$\langle \text{CVC}_{\text{PD}} \rangle$	Average packing domain chromatin volume concentration	0.275
$\langle A_v \rangle$	Average packing domain volume packing efficiency	0.6
$\langle N_{\text{PD}} \rangle$	Average genomic size of a packing domain	380 kbp
r_{fiber}	Median radius of the chromatin fiber	10.0 nm
r_{PD}	Median packing domain radius	110.0 nm
$\langle L_{\text{gene}} \rangle$	Average gene length	6000 bp
$r_{\text{min,in}}$	Radius of the interaction volume of a basepair	15 nm
r_{min}	Radius of a basepair	1 nm
ϕ_m	Mobile crowder volume fraction	0.05
$\langle \bar{\varepsilon} \rangle$	Average expression rate	14.6 nM/s
κ	Critical expression rate below which crowding affects expression	67.5 nM/s
$\tau_{1/2}$	mRNA half-life	10 hours
t_{crit}	Critical time point for committing to cell death	7 hours
τ_2	Cell doubling time	24 hours
$\sigma_{\delta D_n}$	Standard deviation of cell division-induced D_n drift	0.057

Table S3. Summary of effects on chromatin after 48-hour chemotherapy treatments.

Compound	Dose	Cell Line	Number of nuclei	Average D_n	Change in D_n (%)	P value	Mechanism of action
Control	–	A2780	350	2.49	–	–	–
	–	A2780.m248	226	2.63	–	–	–
	–	HCT116	262	2.58	–	–	–
	–	MDA-MB-231	97	2.64	–	–	–
	–	MES-SA	386	2.40	–	–	–
	–	MES-SA.MX2	316	2.51	–	–	–
5-fluorouracil	0.5 µM	A2780	147	2.56	$+2.96$	1.06×10^{-13}	Inhibits thymidylate synthase; disrupts DNA synthesis and repair
	0.5 µM	A2780.m248	100	2.69	$+2.21$	2.21×10^{-5}	
	0.5 µM	MDA-MB-231	81	2.66	$+0.91$	1.14×10^{-1}	
Docetaxel	5 nM	MES-SA	194	2.43	$+0.96$	3.24×10^{-2}	Binds to β-tubulin; stabilizes microtubules; induces mitotic arrest
	5 nM	MES-SA.MX2	82	2.54	$+0.90$	8.16×10^{-2}	
Gemcitabine	50 nM	MES-SA	101	2.50	$+3.91$	6.62×10^{-16}	Inhibits DNA polymerase; terminates DNA chain elongation
	50 nM	MES-SA.MX2	69	2.64	$+5.22$	2.34×10^{-17}	
Oxaliplatin	5 µM	A2780	101	2.65	$+6.60$	7.73×10^{-36}	Forms DNA adducts; induces DNA damage and apoptosis
	5 µM	A2780.m248	85	2.75	$+4.41$	1.48×10^{-15}	
	5 µM	MDA-MB-231	59	2.70	$+2.60$	1.44×10^{-4}	
	15 µM	HCT116	218	2.74	$+6.35$	6.80×10^{-41}	
Paclitaxel	5 nM	A2780	99	2.58	$+3.67$	3.43×10^{-10}	Binds to β-tubulin; stabilizes microtubules; blocks cell cycle progression
	5 nM	A2780.m248	45	2.76	$+4.82$	7.29×10^{-9}	
	5 nM	MDA-MB-231	36	2.73	$+3.54$	9.60×10^{-5}	

Table S4. Summary of effects on chromatin after 30-minute TPR treatments in A2780 cells.

Compound	Dose	Number of nuclei	Average D_n	Change in D_n (%)	P value	Mechanism of action
Control	–	1526	2.42	–	–	–
EGCG	25 nM	276	2.39	-1.46	6.4×10^{-6}	Inhibits DNMT, HDAC1, and HDAC3
Propranolol	200 µM	111	2.39	-1.50	3.9×10^{-3}	Nonselective β-adrenergic receptor antagonist; dephosphorylates histone H3
UNC0638	100 nM	158	2.38	-1.60	2.9×10^{-4}	Inhibits H3K9 methyltransferases G9a and GLP
Resveratrol	35 µM	271	2.38	-1.69	7.7×10^{-7}	Inhibits COX-1/2 and HDAC1-11
Simvastatin	10 µM	261	2.38	-1.69	3.4×10^{-7}	Inhibits HMG-CoA reductase and HDAC1/2
Valproic acid	100 µM	117	2.35	-3.01	2.5×10^{-11}	Blocks voltage-gated ion channels; inhibits HDACs
Sertraline	10 µM	157	2.34	-3.27	1.5×10^{-12}	Inhibits serotonin reuptake
Digoxin	150 nM	130	2.31	-4.74	3.8×10^{-26}	Cardiac glycoside; inhibits Na^+/K^+-ATPase, increasing intracellular Ca^{2+}
Celecoxib	75 µM	132	2.27	-6.08	1.1×10^{-25}	Anti-inflammatory; inhibits voltage-gated Na^+, Ca^{2+}, and K^+ channels; COX-2 inhibitor

References

1. Y Li, et al., Nanoscale chromatin imaging and analysis platform bridges 4D chromatin organization with molecular function. *Sci. Adv.* **7**, eabe4310 (2021).
2. Y Li, et al., Analysis of three-dimensional chromatin packing domains by chromatin scanning transmission electron microscopy (ChromSTEM). *Sci. Reports* **12**, 12198 (2022).
3. EG Iashina, SV Grigoriev, Large-Scale Structure of Chromatin: A Fractal Globule or a Logarithmic Fractal? *J. Exp. Theor. Phys.* **129**, 455–458 (2019).
4. A Eid, et al., Characterizing chromatin packing scaling in whole nuclei using interferometric microscopy. *Opt. Lett.* **45**, 4810–4813 (2020).
5. LM Almassalha, et al., Label-free imaging of the native, living cellular nanoarchitecture using partial-wave spectroscopic microscopy. *Proc. Natl. Acad. Sci.* **113**, E6372–E6381 (2016).
6. L Cherkezyan, et al., Review of interferometric spectroscopy of scattered light for the quantification of subdiffractional structure of biomaterials. *J. Biomed. Opt.* **22**, 030901 (2017).
7. L Cherkezyan, et al., Interferometric Spectroscopy of Scattered Light Can Quantify the Statistics of Subdiffractional Refractive-Index Fluctuations. *Phys. Rev. Lett.* **111**, 033903 (2013).
8. LM Almassalha, et al., Macrogenomic engineering via modulation of the scaling of chromatin packing density. *Nat. Biomed. Eng.* **1**, 902–913 (2017).
9. RKA Virk, et al., Disordered chromatin packing regulates phenotypic plasticity. *Sci. Adv.* **6**, eaax6232 (2020).
10. H Matsuda, GG Putzel, V Backman, I Szleifer, Macromolecular Crowding as a Regulator of Gene Transcription. *Biophys. J.* **106**, 1801–1810 (2014).
11. AR Shim, et al., Dynamic Crowding Regulates Transcription. *Biophys. J.* **118**, 2117–2129 (2020).
12. E Yang, et al., Decay Rates of Human mRNAs: Correlation With Functional Characteristics and Sequence Attributes. *Genome Res.* **13**, 1863–1872 (2003).
13. JC Goldstein, NJ Waterhouse, P Juin, GI Evan, DR Green, The coordinate release of cytochrome c during apoptosis is rapid, complete and kinetically invariant. *Nat. Cell Biol.* **2**, 156–162 (2000).
14. TL Riss, RA Moravec, Use of Multiple Assay Endpoints to Investigate the Effects of Incubation Time, Dose of Toxin, and Plating Density in Cell-Based Cytotoxicity Assays. *ASSAY Drug Dev. Technol.* **2**, 51–62 (2004).
15. R Jessel, S Haertel, C Socaciu, S Tykhonova, HA Diehl, Kinetics of apoptotic markers in exogeneously induced apoptosis of EL4 cells. *J. Cell. Mol. Medicine* **6**, 82–92 (2002).
16. BLL Seagle, et al., TP53 hot spot mutations in ovarian cancer: Selective resistance to microtubule stabilizers in vitro and differential survival outcomes from The Cancer Genome Atlas. *Gynecol. oncology* **138**, 159–164 (2015).
